# Explicit Calculation of Structural Commutation Relations for Stochastic and Dynamical Graph Grammar Rule Operators in Biological Morphodynamics

**DOI:** 10.3389/fsysb.2022.898858

**Published:** 2022-09-09

**Authors:** Eric Mjolsness

**Affiliations:** Departments of Computer Science and Mathematics, University of California, Irvine, CA, United States

**Keywords:** dynamical graph grammar, morphodynamics, operator commutator, cortical microtubule array, actin filament network, synaptic spine head, operator algebra, stochastic graph grammar

## Abstract

Many emergent, non-fundamental models of complex systems can be described naturally by the temporal evolution of spatial structures with some nontrivial discretized topology, such as a graph with suitable parameter vectors labeling its vertices. For example, the cytoskeleton of a single cell, such as the cortical microtubule network in a plant cell or the actin filaments in a synapse, comprises many interconnected polymers whose topology is naturally graph-like and dynamic. The same can be said for cells connected dynamically in a developing tissue. There is a mathematical framework suitable for expressing such emergent dynamics, “stochastic parameterized graph grammars,” composed of a collection of the graph- and parameter-altering rules, each of which has a time-evolution operator that suitably moves probability. These rule-level operators form an operator algebra, much like particle creation/annihilation operators or Lie group generators. Here, we present an explicit and constructive calculation, in terms of elementary basis operators and standard component notation, of what turns out to be a general combinatorial expression for the operator algebra that reduces products and, therefore, commutators of graph grammar rule operators to equivalent integer-weighted sums of such operators. We show how these results extend to “dynamical graph grammars,” which include rules that bear local differential equation dynamics for some continuous-valued parameters. Commutators of such time-evolution operators have analytic uses, including deriving efficient simulation algorithms and approximations and estimating their errors. The resulting formalism is complementary to spatial models in the form of partial differential equations or stochastic reaction-diffusion processes. We discuss the potential application of this framework to the remodeling dynamics of the microtubule cytoskeleton in cortical microtubule networks relevant to plant development and of the actin cytoskeleton in, for example, a growing or shrinking synaptic spine head. Both cytoskeletal systems underlie biological morphodynamics.

## 1 INTRODUCTION

Many emergent, non-fundamental models of complex systems can be described naturally by the temporal evolution of spatial structures with some nontrivial discretized topology, such as a graph with suitable discrete and/or continuous state-determining parameter vectors labeling its vertices. In materials science, there can be dynamic networks of fractures or extended crystal defects. Biological examples include the network of adjacent cells in a tissue or the dynamic polymeric cytoskeleton within a single cell. Such biological examples arise in development, where one has morphodynamics (dynamics of the form) at both the tissue and cellular level, and they are interrelated. In this study, our examples will mainly be taken from the domain of graph-like structural dynamics in the cytoskeleton, in these two domains of biological pattern formation and morphodynamics (Vos et al., 2004; Hotulainen and Hoogenraad, 2010; Sampathkumar et al., 2014; Chakrabortty et al., 2018; Bonilla-Quintana et al., 2020).

In previous work [(Mjolsness, 2019a), Propositions 1 and 2], we showed that the parameterized or labeled graph rewrite rule operator semantics specified there (in two versions, one without and one with hanging edge removal) is contained within a somewhat larger operator algebra closed under addition, scalar multiplication, and operator multiplication (and hence under commutation, as in a Lie algebra).

The purpose of this study is to show explicitly and combinatorially what this operator algebra is: under either semantics (hanging edges removed or not), the vector space spanned by the graph rewrite rule operators previously defined form an operator algebra and a Lie algebra among all such graph rewrite rule operators, under an explicit formula to be presented in Section 2.4. In particular, the product of the state-changing portions of two such operators can be written as a sum of such operators with nonnegative integer weights, and the full product and commutator of two such operators can be written as a sum of such operators with integer weights.

These results arise within a larger scientific scope discussed at length in Mjolsness (2019a), including grammar-like or rule-based structured models of molecular complexes (Blinov et al., 2004) and of tissues with dividing cells (Mjolsness et al., 1991; Prusinkiewicz et al., 1993). Potential applications include cytoskeletal dynamics in cellular and developmental biology, neurobiology, and smart materials, as well as the dynamics of more abstract, non-spatial graphs in a wide variety of fields. We will illustrate with subcellular cortical microtubule biophysical dynamics that are important at the cellular and tissue level of plant development.

Given state-changing operators ${\hat{W}}_{r}$ for the rules in grammar, for example, as outlined in Section 2.2, the Master Equation for the stochastic dynamics is as follows (Mjolsness and Yosiphon, 2006):

(1a) $$\frac{d\;p}{dt}=W\cdot p,\;\;(\text{probability flows according to}\;W),\;\text{where}$$

(1b) $$W=\sum_{r}W_{r},\;\;(\text{rule operators sum up})$$

(1c) $$W_{r}\equiv {\hat{W}}_{r}\text{−}D_{r},\;\;(\text{rules conserve probability})$$

(1d) $$D_{r}\equiv \text{diag}\left(1\cdot {\hat{W}}_{r}\right)\;\;(\text{total probability outflow per state})$$

[generalizing (Doi, 1976a; Doi, 1976b; Mattis and Glasser, 1998) for stochastic chemical reaction networks], where probability is defined over a suitable Fock space for varying numbers of graph nodes (with labels) and graph edges. Supplementary Section SC discusses how this framework can be used to model stochastic chemical reaction networks, using the algebras of elementary and compound *W*_*r*_ operators.

In this study, the goal is to explicitly calculate the key operator algebra identity for such operators ${\hat{W}}_{r}$, as exhibited in Eq. 16 of Section 2.4, with important corollaries in Sections 3.4, 3.5, and proven in Section 3 and Supplementary Material SA, and to extend it to the differential equations case. The exposition will be organized in three successive levels of detail: first a statement of the main results (Section 2), then a sketch of the general computations and theorems, including their corollaries (Section 3), then a collection of examples (Section 3.7), followed by Supplementary Material, which refines the explicit operator semantics and contains the full calculations.

## 2 PROBLEM STATEMENT

We first recapitulate the required operator algebra definitions and then state our problems. In Section 2.1, we will define graphs, labeled graphs, and graph grammars. In Section 2.2, we will use operator algebra to define the semantics of graph grammar rules and graph grammars. Then, in Section 2.3, we will state the operator algebra problems, and in Section 2.4, we will preview the main results of the study. The methods in this study will be purely theoretical: performing operator algebra calculations that establish concise results that solve the stated problems.

### 2.1 Graph Grammar Rule Syntax

The definitions of this section informally summarize the more systematic definitions of Mjolsness (2019a) (Supplementary Material). A graph is an unordered set *V* of “vertices” or “nodes,” together with a set *E* of “edges” or “links,” each of which is, or corresponds to, either 1) an unordered pair of vertices {*u, v*}, for an “undirected edge,” 2) an ordered pair of vertices (*u, v*), for a “directed edge,” or 3) a singleton vertex {*v*} (or equivalently the ordered pair (*v, v*)), for a “self-edge.” An unordered pair of vertices cannot have both directed and undirected edges, except in the sense that a pair of oppositely directed edges can represent an unordered edge. An “undirected graph” has only undirected edges; a “directed graph” has only directed edges; either kind can allow self-edges or not. This notion of a graph encompasses undirected graphs and directed graphs, with or without self-loops, in a way that is compatible with the computational representation of a graph as an adjacency matrix.

A labeled graph adds the extra structure of a mapping from vertices in *V* to labels in label set Λ. Labeled graphs (with node labels as above) can be used to encode and implement many other kinds of graphs, such as multigraphs, edge-labeled graphs mapped to bipartite- (node-) labeled graphs, hypergraphs, and abstract cell complexes.

More technically, a numbered graph is a labeled graph in which the label set is an initial subset Λ′ = {1, …*n*} of the natural numbers, and the assigned node labels are unique (so ∣Λ′∣≥∣*V*∣). In this case, there is an induced total ordering on the vertex set *V*, breaking the prima facie permutation invariance of the vertices of the graph. If all numbers in ∣Λ′∣ are assigned (so ∣Λ′∣ = ∣*V*∣ by 1-1 correspondence), then such a numbered graph can be represented uniquely by a 0/1-valued adjacency matrix recording the presence or absence of directed edges (*i* = *λ*(*u*), *j* = *λ*(*v*)), where *i* and *j* are integer-valued “index” labels, with undirected edges encoded by the presence of two oppositely directed edges and self-edges recorded by diagonal matrix entries. The case ∣Λ′∣ > ∣*V*∣ is required just to define a consistent numbering of several graphs, not all of whose vertices can be identified across graphs.

A labeled graph can be represented (perhaps nonuniquely) by a numbered graph *G* together with a vector of labels ⟪*λ*_1_, …*λ*_*i*_, …*λ*_*n*_⟫ that map vertex indices *i* to vertex labels; the resulting labeled graph combination is denoted *G*⟪*λ*_1_, …*λ*_*i*_, …*λ*_*n*_⟫. Elements *λ* of the label set Λ can themselves take the form of a vector or tuple with *d* components; if *d* = 0, then there is only one label and the labeled graph is equivalent to an unlabeled graph again.

Given these definitions, the “syntax” of a graph grammar rewrite rule takes a form involving two labeled graphs that have been decomposed into two consistently numbered graphs and their label maps:

(2) $$G⟪\lambda_{1},\ldots \lambda_{n}⟫\to G^{\prime }⟪\lambda_{1}^{\prime },\ldots \lambda_{n^{\prime }}^{\prime }⟫\;\;\mathrm{with}\;\;\rho (\lambda_{1},\ldots \lambda_{n},\lambda_{1}^{\prime },\ldots \lambda_{n^{\prime }}^{\prime }).$$

Such an expression represents a discrete local transformation that can act or “fire” anywhere that the left-hand side (LHS) labeled graph *G*⟪*λ*_1_, …*λ*_*i*_, …*λ*_*n*_⟫ matches (occurs as a labeled subgraph, with matching edge structure and labels) within a potentially much larger system graph that comprises the current state of a system model. Of course, many rule firings may be possible for a given rule and system graph; it is up to the semantics outlined below to determine what actually happens with what probability and when. That will depend on the non-negative function *ρ*, the propensity, or rule firing probability per unit time. By making *ρ* a function of the *λ*s, we allow that one syntactic rule, as above, can specify many grounded rules, each of which has all *λ*s replaced with constant values, as in the integration semantics provided in the next section. The integrable measure spaces in which labels *λ*_*i*_ live were outlined in Mjolsness and Yosiphon (2006).

Such a graph rewrite rule is expressed in terms of a single consistent numbering of the vertices of the two numbered graphs. Therefore, vertices in *G* and *G′* that share a vertex number are regarded as “the same” vertex *v*, before and after rewriting, and any graph edges contacting *v* but not mentioned in the rewrite rule are preserved. In this way, graph rewrite rules can operate within a broader graph context. On the other hand, the particular consistent numbering chosen is arbitrary and does not matter. The semantics in the next section will be invariant with respect to permutations of the consistent numbering.

For example, Eq. 3 below specifies a part of the refinement process for 2D triangular meshes. Each graph node bears an integer parameter *l* denoting a local level number for the depth of refinement. This rewrite rule is one of four that suffice to implement a standard triangular mesh refinement scheme. The other three rules handle partially refined triangle edges, an unavoidable consequence of the previous refinement of adjacent triangles. Further details are provided by Mjolsness (2019a). The labeled graph rewrite rule is 
(3)

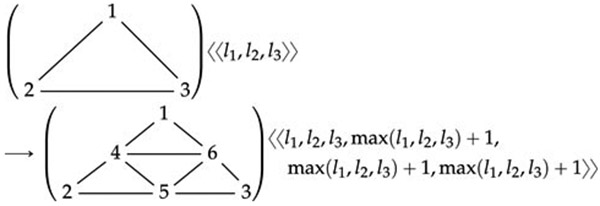

 with some constant propensity *ρ* (omitted). Of course, it is also possible to provide a linear, textual representation of a numbered graph *G*, if only as a list of its edges between ordered pairs of index values.

### 2.2 Graph Grammar Rule Semantics

Let indices *i*_1_, …*i*_*k*_ range over many graph nodes that can each be allocated to model the state of some object in a modeling domain.

In the following, as elaborated in Mjolsness (2010) and Mjolsness (2013), stochastic labeled graph grammar (SLGG) rule semantics with vectors ***λ***, ***λ′*** of incoming and outgoing graph node labels can be thought of as stochastic parameterized graph grammar (SPGG) semantics when the labels are taken to be functions ***λ***(*X*) and ***λ′***(*X*) of some vector of parameters or variables *X*. The rule semantics is obtained by integrating over all possible values of a vector of rule variables *X* that appear in the graph labels *λ*, *λ′*; as a special case, some labels and/or parameters could be constant. Then,

(4) $${\hat{W}}_{r}=\int d\mu_{r}(X){\hat{W}}_{r}\left(\lambda (X),\lambda^{\prime }(X)\right),$$

where *μ*_*r*_(*X*) is a suitable measure that could be discrete in one or several dimensions (so, the integral becomes a sum or multiple sum) or continuous in one or several dimensions (so the integral may be a multiple integral), or a multidimensional combination of discrete and continuous components. However, any continuous measures can be approximated by discrete ones to retain the essentially combinatorial nature of the proofs below. In addition, the label functions ***λ***(*X*) and ***λ′***(*X*) can include extra components, which are constant, for the given rule number *r*. These are not to be integrated over, so they are not part of the variable *X*.

We provided examples of such graph grammar rules for mesh refinement in Mjolsness (2019a) and will exhibit graph grammar rules for coarse-grained models of plant cortical microtubule dynamics in Section 3.7.1.

Consider a graph rewrite rule expressed, in part, as *G*^*r* in^(***λ***(*X*)) → *G*^*r* out^(***λ′***(*X*)), where *G*^*r* in^ and *G*^*r* out^ are graphs with the given vectors of labels and an arbitrary but shared numbering of their nodes. Define “∑_⟨*i*_1_,…*i_k_*⟩__≠_…” to be a sum over indices (*i*_1_, …*i*_*k*_) constrained so that each *i_l_* is unequal to all the others. Then, in the simplest case (but see Supplementary Section SA.4.3), we define the time-evolution operator of a graph rewrite rule:

(5) $${\hat{W}}_{r}=\frac{1}{C_{r}\;(N_{\max\;\text{free}})}\int d\mu_{r}\;(X)\;\rho_{r}\left(\lambda (X),\lambda^{\prime }(X)\right)\;\;\times \sum_{\langle i_{1},\ldots i_{k}\rangle_{\neq }}{\hat{a}}_{i_{1},\ldots i_{k}}\;(G^{r\;\text{out}})a_{i_{1},\ldots i_{k}}\;(G^{r\;\text{in}})$$

where, as explained by Mjolsness (2019a), the graph grammar rule operator first annihilates all the edges and labeled nodes in the incoming “left hand side” graph *G* = *G*^*r* in^ and then, but uninterruptibly and with zero time delay, creates the corresponding elements of the outgoing “right-hand side” graph *G′* = *G*^*r* out^:

(6) $${\hat{a}}_{i_{1},\ldots i_{k}}(G^{\prime })={\hat{a}}_{i_{1},\ldots i_{k}}(G_{\text{links}}^{\prime }){\hat{a}}_{i_{1},\ldots i_{k}}(G_{\text{nodes}}^{\prime })\;\;=\left[\prod_{s^{\prime },t^{\prime }\in \text{rhs}(r)}{\left({\hat{a}}_{i_{s^{\prime }}i_{t^{\prime }}}\right)}^{g_{\;s^{\prime }t^{\prime }}^{\prime }}\right]\left[\prod_{v^{\prime }\in \text{rhs}(r)}{\hat{a}}_{i_{v^{\prime }}\lambda_{\;v^{\prime }}^{\prime }}\right]\;\;=\left[\prod_{\left(s^{\prime },t^{\prime }\right)\in G_{\text{links}}^{\prime }}{\hat{a}}_{i_{s^{\prime }}i_{t^{\prime }}}\right]\left[\prod_{v^{\prime }\in G_{\text{nodes}}^{\prime }}{\hat{a}}_{i_{v^{\prime }}\lambda_{\;v^{\prime }}^{\prime }}\right]\;\;a_{i_{1},\ldots i_{k}}\;(G)=a_{i_{1},\ldots i_{k}}\;(G_{\text{links}})a_{i_{1},\ldots i_{k}}(G_{\text{nodes}})=\left[\prod_{s,t\in \text{lhs}(r)}{\left(a_{i_{s}i_{t}}\right)}^{g_{st}}\right]\left[\prod_{v\in \text{lhs}(r)}a_{i_{v}\lambda_{v}}\right].\;\;=\left[\prod_{(s,t)\in G_{\text{links}}}a_{i_{s}i_{t}}\right]\left[\prod_{v\in G_{\text{nodes}}}a_{i_{v}\lambda_{v}}\right].$$

The sets lhs_*r*_ and rhs_*r*_ comprise the nodes or vertices in the left-hand side and right-hand side graphs, *G* and *G′*, with adjacency matrices *g* and *g′*, of rule *r*. The creation and annihilation operators ${\hat{a}}_{\alpha }$ and *a*_*α*_ are the 2 × 2 {0, 1}-valued matrices that add or remove a “particle” (a graph node or link) if possible and, otherwise, yield a probability vector of zero in a many-particle-type Fock space (Mjolsness and Yosiphon, 2006, Sections 3.2, 3.3), simplified from Reed and Simon (1980). They are closely related to the Doi formulation of chemical reaction networks (Doi, 1976a; Doi, 1976b; Mattis and Glasser, 1998) described in Supplementary Material SC, as discussed by Mjolsness and Yosiphon (2006), except that the maximum number of identical “particles” of each subscript combination is taken to be one rather than countable infinity. Node labels *λ*_*v*_ take values in a discrete set or a continuum well approximable in computational implementations by discrete sets such as the set of floating-point numbers. The “probabilistic Fock spaces” comprising probability distributions over graph nodes and edges, on which all these operators act, apply to discrete and/or continuous node labels, including edge information.

The two matrices *g* and *g′* share the same consistent numbering of graph nodes (i.e., graph vertices) so that a given node number *s* can be directionally connected to other nodes *t* in graph *g* iff *g_s t_* = 1, graph *g′* iff *g′_s t_* = 1, or both; and the corresponding individual links (i.e., graph edges) given by nonzero entries in these two matrices can be independently present or absent. Because of the “∑_⟨*i*_1_,…*i_k_*⟩__≠_…” form of this operator (Eq. 5), each such operator is invariant under any global permutation Σ operating on the object-modeling domain graph nodes indexed by *i_s_*, and it is also invariant under any permutation *σ* operating on the consistent graph numbering of the graph rule nodes indexed by *s, t*. This permutation invariance is essential in making the rewrite rule apply to graphs, which do not have an intrinsic ordering to their vertices. However, the permutation symmetry can be and usually is partially broken by graph labels and/or connectivity. The normalizing factor of 1/*C_r_*(*N*_max free_) in Eq. 5 may be required to account for the numbering degeneracy of possible new graph nodes added by the right-hand side graph, as shown in Supplementary Section SA.2.

The denominator 1/*C_r_*(*N*_max free_) in Eq. 5, like the sum over permutations “∑_⟨*i*_1_,…*i_k_*⟩__≠_…” helps account for the change of representation between abstract graphs with their unordered nodes, and computer-representable nodes that are associated with an arbitrary but ordered integer index *i*_*k*_ such as location in computer memory. In particular, the representation of one or more new graph nodes required by the firing of rule *r* must be drawn from some available pool of one or more available indexed nodes. This is an arbitrary choice. 1/*C_r_*(*N*_max free_) counts the number of ways this choice can be made, weighted equally, and ensures their total propensity adds up to what is required by the rest of the expression in Eq. 5. The actual count depends on the details of memory management as discussed in Supplementary Sections SA.2, SA.4.2; it could be as low as *C_r_* = 1, but that may require a serial implementation of the simulation computation.

Undirected graphs can be encoded as a special case in which matrix *g* is symmetric. Node- and edge-labeled graphs can be encoded as a special case in which node labels come in two colors, the graph is bipartite (alternating node-colored with edge-colored nodes), and all the edge-colored nodes have degree two.

Another useful form of Eq. 5 is to factor out any graph *K* that is completely unchanged. This form is exhibited in Supplementary Section SA.1.

Returning to Eqs 5, 6, we can combine them to write out more explicitly

(7) $${\hat{W}}_{r}=\frac{1}{C_{r}\;(N_{\max\;\text{free}})}\int d\mu_{r}\;(X)\;\rho_{r}\left(\lambda (X),\lambda^{\prime }(X)\right)\;\;\times \sum_{\langle i_{1},\ldots i_{k}\rangle_{\neq }}\left[\prod_{s^{\prime },t^{\prime }\in \text{rhs}(r)}{\left({\hat{a}}_{i_{s^{\prime }}i_{t^{\prime }}}\right)}^{g_{\;s^{\prime }t^{\prime }}^{\prime }}\right]\left[\prod_{v^{\prime }\in \text{rhs}(r)}{\hat{a}}_{i_{v^{\prime }}\lambda_{\;v^{\prime }}^{\prime }}\right]\;\;\times \left[\prod_{s,t\in \text{lhs}(r)}(a_{i_{s}i_{t}}{)}^{g_{s\;t}}\right]\left[\prod_{v\in \text{lhs}(r)}a_{i_{v}\lambda_{v}}\right].$$

Two models defined by the Master Equation (ME) will be “equivalent” if their state variables can be identified so that solutions of the Master Equation are identical in all statistically observable respects: in all moments of all number operators at all choices of observation times. If *α* indexes the observable numbers *n*_*α*_ of objects and relationships and *N*_*α*_ is the corresponding number operator, then we can read out a broad range of joint probabilities with the moments of Kronecker delta functions:

(8) $${\text{Pr}}_{\text{ME}}\left(\left[n_{\alpha \left(q\right)}\left(t_{q}\right)|q\right]\right)={\langle \prod_{q}\delta \left(N_{\alpha \left(q\right)}\left(t_{q}\right)-n_{\alpha \left(q\right)}I_{\alpha \left(q\right)}\right)\rangle }_{\text{ME}}$$

where a collection indexed by *q* of values *n*_*α*(*q*)_ of number operators *N*_*α*(*q*)_ are measured at times *t*_*q*_ and the ensemble average taken. As the operative definition of equivalence, we demand equality of all such moments. Other observables ⟨*f* ([*N*_*α*(*q*)_(*t_q_*)∣*q*])⟩_ME_ (where *f* is applied component-wise to diagonal matrices) follow from Eq. 8 as a linear basis.

#### 2.2.1 Application to ODE Rules

There is a natural application of the foregoing class of operator to incorporate ordinary differential equation (ODE) dynamics on parameters appearing in the graph labels, for example, the positions and other continuous state information of particles denoted by labeled graph nodes. We define a stochastic parameterized graph grammar incorporating differential equation bearing rules as dynamical graph grammar (DGG). Suppose the concatenated vector ***x*** of real-valued node parameters in a local graph neighborhood matching graph *G*^*r*^(***x***), which is otherwise unchanged from the left-hand side to right-hand side of the rule, obeys the coupled differential equation system *d**x**/dt* = ***v***(***x***). As shown by Eq. 21 in Mjolsness (2013), using Dirac delta functions in a physicist’s style of calculation rather than a mathematical analyst’s, it suffices to consider an operator of an especially simple form, with the same graph nodes and edges on the left and right sides, and changes only to node labels:

(9a) $${\hat{W}}_{\text{ODE}\;r}={\hat{W}}_{r}=\int d\mu_{r}\;(x)d\mu_{r}\;(y)\;\rho_{r}\;(y,x)\;\;\sum_{\langle i_{1},\ldots i_{k}\rangle_{\neq }}{\hat{a}}_{i_{1},\ldots i_{k}}\;(G^{r}\;(y))a_{i_{1},\ldots i_{k}}(G^{r}\;(x)),\;\text{where}$$

(9b) $$\;\rho_{r}\;(y,x)=-\nabla_{y}\cdot \left(v(y)\delta (y-x)\right)\;\;=-\sum_{a}\nabla_{y_{a}}\left(v_{a}(y)\prod_{b}\delta (y_{b}-x_{b})\right).$$

It is important that the combined definitions of integration measure *μ*, derivative ∇, and Dirac delta function *δ* should support integration by parts in Eq. 9, as, for example, Lebesgue does with the usual derivative operator and Dirac delta choices. We will assume the same can be said for whatever finite approximation of differential equation solving is to be run on a computer implementation, noting in support of this assumption the extensive literature on summation by parts and its generalization to memetic differential equation solution methods satisfying the identities ofvector differential and integral calculus (Corbino and Castillo, 2020); as further support, we have noted the existence of a DGG simulation algorithm with a running implementation (Yosiphon, 2009; Mjolsness, 2013).

Clearly, the top line of Eq. 9 is a special case of our general algebraic form for ${\hat{W}}_{r}$ if Dirac delta functions are admitted into the expressions for *ρ*_*r*_. Moreover, if not, we can take a suitable *σ* → 0 parametric limit of width-*σ* Gaussians to approach all the Dirac deltas at the end of all other calculations. This expression (Eq. 9) is already flux-balanced, so the corresponding *D*_ODE *r*_ = 0 and $W_{\text{ODE}\;r}={\hat{W}}_{\text{ODE}\;r}$. However, an important mathematical difference is that these *ρ* functions can no longer be guaranteed to be non-negative because velocities *v* in the ODEs have no sign restriction. A second important mathematical difference will be encountered in the commutation relations for creation/annihilation operators parameterized by the below real-valued labels: Kronecker deltas become Dirac deltas. The resulting approach based on Eq. 9 leads to Proposition 1 of Section 3.6.

In this way, the proofs of Theorems 1 and 2 remain essentially unchanged, but their function spaces are reinterpreted to yield a nontrivial generalization in the expressive power of the rules, generalizing from stochastic parameterized graph grammars to dynamical graph grammars. A simulation algorithm for dynamic graph grammars is described in Mjolsness (2013). Mjolsness and Yosiphon (2006) and Mjolsness (2010) also show how to further extend this approach of Eq. 9 to stochastic differential equations (SDEs).

#### 2.2.2 Products and Commutators of Graph Rewrite Operators

From Eqs 5, 6, we can compute the product:

(10) $${\hat{W}}_{r_{2}}{\hat{W}}_{r_{1}}=\frac{1}{C_{r_{1}}\;(N_{\max\;\text{free}})}\frac{1}{C_{2}\;(N_{\max\;\text{free}})}\iint_{}d\mu_{r_{1}}\;(X_{1})d\mu_{r_{2}}\;(X_{2})\;\rho_{r_{1}}\left(\lambda_{1}(X_{1}),\lambda_{1}^{\prime }(X_{1})\right)\;\;\times \rho_{r_{2}}\left(\lambda_{2}(X_{2}),\lambda_{2}^{\prime }(X_{2})\right)\sum_{\langle j_{1},\ldots j_{k_{2}}\rangle_{\neq }}\sum_{\langle i_{1},\ldots i_{k_{1}}\rangle_{\neq }}{\hat{a}}_{j_{1},\ldots j_{k_{2}}}\;(G_{\text{links}}^{r_{2}\;\text{out}})\;\;\times {\hat{a}}_{j_{1},\ldots j_{k_{2}}}\;(G_{\text{nodes}}^{r_{2}\;\text{out}})a_{j_{1},\ldots j_{k_{2}}}\;(G_{\text{links}}^{r_{2}\;\text{in}})a_{j_{1},\ldots j_{k_{2}}}(G_{\text{nodes}}^{r_{2}\;\text{in}})\times {\hat{a}}_{i_{1},\ldots i_{k_{1}}}\;(G_{\text{links}}^{r_{1}\;\text{out}})\;\;\times {\hat{a}}_{i_{1},\ldots i_{k_{1}}}\;(G_{\text{nodes}}^{r_{1}\;\text{out}})a_{i_{1},\ldots i_{k_{1}}}\;(G_{\text{links}}^{r_{1}\;\text{in}})a_{i_{1},\ldots i_{k_{1}}}\;(G_{\text{nodes}}^{r_{1}\;\text{in}}),$$

and consequently,

(11) $${\hat{W}}_{r_{2}}{\hat{W}}_{r_{1}}=\frac{1}{C_{r_{1}}\;(N_{\max\;\text{free}})}\frac{1}{C_{2}\;(N_{\max\;\text{free}})}\iint_{}d\mu_{r_{1}}\;(X_{1})d\mu_{r_{2}}\;(X_{2})\;\rho_{r_{1}}\left(\lambda_{1}(X_{1}),\lambda_{1}^{\prime }(X_{1})\right)\;\;\times \rho_{r_{2}}\left(\lambda_{2}(X_{2}),\lambda_{2}^{\prime }(X_{2})\right)\sum_{\langle j_{1},\ldots j_{k_{2}}\rangle_{\neq }}\sum_{\langle i_{1},\ldots i_{k_{1}}\rangle_{\neq }}{\hat{a}}_{j_{1},\ldots j_{k_{2}}}\;(G_{\text{links}}^{r_{2}\;\text{out}})\;\;\times \left[a_{j_{1},\ldots j_{k_{2}}}\;(G_{\text{nodes}}^{r_{2}\;\text{in}}){\hat{a}}_{i_{1},\ldots i_{k_{1}}}\;(G_{\text{links}}^{r_{1}\;\text{out}})\right]a_{i_{1},\ldots i_{k_{1}}}(G_{\text{links}}^{r_{1}\;\text{in}})\;\;\times {\hat{a}}_{j_{1},\ldots j_{k_{2}}}\;(G_{\text{nodes}}^{r_{2}\;\text{out}})\left[a_{j_{1},\ldots j_{k_{2}}}\;(G_{\text{nodes}}^{r_{2}\;\text{in}}){\hat{a}}_{i_{1},\ldots i_{k_{1}}}\;(G_{\text{nodes}}^{r_{1}\;\text{out}})\right]a_{i_{1},\ldots i_{k_{1}}}\;(G_{\text{nodes}}^{r_{1}\;\text{in}}).$$

We now discuss the emergence of a new combined propensity function *ρ*_*r*_2;1__ (*Y*_2_, *Z*, *X*_1_) for the product of rule operators in Eq. 11, which will arise from delta functions that appear in commutators of elementary operators. The form of *ρ*_*r*_2;1__ is given in Eq. 14.

In general, the commutator of elementary operators will either be zero or proportional to a Kronecker or Dirac delta function, which removes one of the multiple summations or integrations over parameters in the foregoing expression. For example, in the case of continuous parameters *X*, we may have *dμ*(*X*) = Lebesgue measure, encountering Dirac delta functions arising from the operator algebra:

(12) $$\int dy_{2}dx_{2}\int dy_{1}dx_{1}\rho_{r_{2}}(y_{2},x_{2})\rho_{r_{1}}(y_{1},x_{1})\delta^{\text{Dirac}}(x_{2}-y_{1})𝒪(y_{2},x_{2},y_{1},x_{1})\;\;=\int dy_{2}dzdx_{1}\rho_{r_{2;1}}(y_{2},z,x_{1})𝒪(y_{2},z,z,x_{1}).$$

Likewise, for discrete label variables, we will have *dμ*(*X*) = a discrete measure so that the integral is a sum, together with Kronecker deltas arising from the operator algebra:

(13) $$\sum_{\alpha_{2}\;\beta_{2}}\sum_{\alpha_{1}\;\beta_{2}}\rho_{r_{2}}\;(\beta_{2},\alpha_{2})\rho_{r_{1}}\;(\beta_{1},\alpha_{1})\delta^{\text{Kronecker}}(\alpha_{2},\;\beta_{1})𝒪(\beta_{2},\alpha_{2},\beta_{1},\alpha_{1})\;\;=\sum_{\beta_{2}\;\gamma \;\alpha_{1}}\rho_{r_{2;1}}\;(\beta_{2},\gamma ,\alpha_{1})𝒪(\beta_{2},\gamma ,\gamma ,\alpha_{1})$$

where 𝒪 is a suitable operator expression and where scalar functions combine simply by multiplication and delta-induced parameter substitution:

(14) $$\rho_{r_{2;1}}(Y_{2},Z,X_{1})\equiv \rho_{r_{2}}(Y_{2},Z)\rho_{r_{1}}(Z,X_{1}),$$

where the capital letter parameters are vectors of discrete and/or continuous parameters. Eq. 14 preserves the non-negativity of *ρ* scalar functions if rules *r*_1_ and *r*_2_ have it. Following Eq. 10, the variables *Z* will subsequently be integrated out. This parallelism between discrete and continuous versions of the identity ∫*dμ*(*x*)*δ*(*x* − *y*)*f*(*x*) = *f*(*y*) is the fundamental reason that Theorems 1 and 2 can be extended to the continuous case described in Proposition 1.

Given a formula for the product ${\hat{W}}_{r_{2}}{\hat{W}}_{r_{1}}$ of two (in general non-elementary) graph rewrite rule operators, their commutator is of course just

(15) $$\left[{\hat{W}}_{r_{2}},{\hat{W}}_{r_{1}}\right]={\hat{W}}_{r_{2}}{\hat{W}}_{r_{1}}-{\hat{W}}_{r_{1}}{\hat{W}}_{r_{2}}$$

The products and commutators of full probability-conserving rule operators of the form $W_{r}={\hat{W}}_{r}-D_{r}$ also follow directly. Nevertheless, the operator commutator is mathematically a fundamental object.

### 2.3 Three Problems to Solve

We can now state the central problems of this study:

1. Up to equivalence, can the product of two graph grammar rewrite rule operators be expressed in terms of a sum of such operators, and if so, how?
2. Likewise for the commutator of such operators: up to equivalence, can the commutator of two graph grammar rewrite rule operators be expressed in terms of a sum of such operators, and if so, how?
3. Do these results extend to dynamical graph grammars, which by definition include rules that bear differential equations?

### 2.4 Preview of Main Results

After a calculation and several arguments, the main result that answers the foregoing questions will be an operator algebra equivalence that turns a product of graph rewrite operators into a sum of other graph rewrite operators. The required sum is taken over two sets of recognizable combinatorial objects: first, the possible edge-maximal subgraphs *H* in the output side of rule *r*_1_ that match the structure and labels of some subgraph $\hat{H}$ of the input side of rule *r*_2_, representing their possible overlap of rule firing action, and second, the one or more possible distinct maps *h* along which such a one-to-one matching can occur. The equation is

(16) $${\hat{W}}_{G^{r_{2}\;\text{in}}\to G^{r_{2}\;\text{out}}}{\hat{W}}_{G^{r_{1}\;\text{in}}\to G^{r_{1}\;\text{out}}}≃\sum_{\begin{array}{c} H\subseteq G^{r_{1}\;\text{out}}≃\tilde{H}\subseteq G^{r_{2}\;\text{in}}\\ \;|\;\text{edge}-\text{maximal} \end{array}}\sum_{h:H\overset{1-1}{↪}\tilde{H}}{\hat{W}}_{G^{1;2\;\text{in}}\left(\tilde{H}\right)\;\underset{h}{\to }\;G^{1;2\;\text{out}}(H)}$$

where the new labeled graphs, roughly given by

(17) $$\;G^{1;2\;\text{in}}\left(\tilde{H}\right)=G^{r_{1}\;\text{in}}\dot{\cup }\left(G^{r_{2}\;\text{in}}\setminus \tilde{H}\right)\;G^{1;2\;\text{out}}(H)=G^{r_{2}\;\text{out}}\dot{\cup }\left(G^{r_{1}\;\text{out}}\setminus H\right),$$

and their labeled graph overlap will be defined more carefully in Section 3. The binary set difference “\” and disjoint union “$\overset{\cdot }{\cup }$” operators apply directly to the vertices in the respective graphs but extend to all associated edges to result in valid graphs. Scalar functions *ρ*_*r*_ will combine by multiplication and parameter substitution, as in Eq. 14. Note that all integer weights on the left-hand side of Eq. 16 are nominally zero or one. However, because the same or equivalent operators could arise multiple times, the weights are actually nonnegative integers.

In this way, the operator algebra of graph rewrite rules is “lifted” from the level of creation/annihilation operators on elementary binary random variables to the more abstract and structural level of well-formed labeled graph rewrite rules.

This result will be shown without (Theorem 1, Section 3.4, and Supplementary Section SA.5) and with (Theorem 2, Section 3.5, and Supplementary Section SA.6) hanging edge cleanup semantics. First (Sections 3.1-3.3 and Supplementary Sections SA.2-SA.4), we will discuss some of the used operator algebra calculational techniques and strategies without claiming any optimality for them.

As direct corollaries (Corollaries 1 and 5, Sections 3.4, 3.5), the full operators $W_{r}={\hat{W}}_{r}-D_{r}$ obey a similar product ≃ integer-weighted sum operator equivalence, except that the integer-weighted sum over graph rewrite rule operators on the right-hand side can have both positive and negative integer weights. Also, as direct corollaries (Corollaries 2 and 6, Sections 3.4, 3.5), the same is true for the commutators:

(18) $$\left[{\hat{W}}_{G^{r_{2}\;\;\text{in}}\to G^{r_{2}\;\;\text{out}}},{\hat{W}}_{G^{r_{1}\;\;\text{in}}\to G^{r_{1}\;\text{out}}}\right]≃\sum_{\begin{array}{c} H\subseteq G^{r_{1}\;\text{out}}≃\tilde{H}\subseteq G^{r_{2}\;\text{in}}\\ \;H\neq \emptyset \;\;\wedge \;\;\text{edge}-\text{maximal} \end{array}}\sum_{h:\;H\overset{1-1}{↪}\tilde{H}}{\hat{W}}_{G^{1;2\;\;\text{in}}\left(\tilde{H}\right)\;\underset{h}{\to }\;G^{1;2\;\;\text{out}}(H)}\;\;-\sum_{\begin{array}{c} H\subseteq G^{r_{2}\;\text{out}}≃\tilde{H}\subseteq G^{r_{1}\;\text{in}}\\ \;H\neq \emptyset \;\;\wedge \;\;\text{edge}-\text{maximal} \end{array}}\sum_{h:\;H\overset{1-1}{↪}\tilde{H}}{\hat{W}}_{G^{2;1\;\;\text{in}}\left(\tilde{H}\right)\;\underset{h}{\to }\;G^{2;1\;\;\text{out}}(H)}$$

except that the integer-weighted sum over graph rewrite rule operators on the right-hand side can have both positive and negative integer weights, and the *H* = ∅ terms always drop out.

In addition, in the course of proving these two theorems, we exhibit in each case a constructive mapping (Corollaries 3 and 7, Sections 3.4, 3.5) from the graph rewrite rule operator algebra semantics to the elementary bitwise (two-state) operator algebras of Supplementary Section SA.3.1.

Finally, Corollaries 4 and 8 (Sections 3.4, 3.5) point out that *H* = ∅ cancels out all the commutators of Corollaries 2 and 6.

Theorems 1 and 2 will extend straightforwardly, as stated in Proposition 1, to the dynamical graph grammar (DGG) case, in which some rule operators express dynamical systems in the form of systems of ordinary differential equations, as sketched in Section 2.2.1 and Eq. 14. In like manner, some rules could be SDE-bearing rules whose operator expression is given in Mjolsness and Yosiphon (2006) and Mjolsness (2010).

## 3 RESULTS

In this section, we will sketch the main calculations of this study, which appear in much greater detail in Supplementary Material SA, interleaved with mathematical statements of the results of those calculations. The sketch will take the following form: 1) preliminary definitions and notation, including the two different graph grammar operator semantics that differentiate Theorem 1 from Theorem 2 (Sections 3.1-3.3); 2) an operator product problem statement for the first semantics, followed by the statements of Lemma 1 and Theorem 1 each followed by a link to Supplementary Material SA for its proof, followed by a series of four corollaries with short proofs (Section 3.4); 3) an operator product problem statement for the second semantics, followed by a proof sketch for the removal of hanging edges, followed by the theorem statement of Theorem 2 and a link to Supplementary Material SA for its full proof, which expands on but does not depend on the proof sketch, followed by a series of four corollaries with short proofs (Section 3.5); 4) further observations based on earlier equations that are gathered together to prove Proposition 1, followed by the statement of Proposition 1 (Section 3.6). In addition, we will provide selected example calculations (Section 3.7) involving cytoskeleton in plant cells and synapses.

For the sketch, we will set 1/*C_r_*(*N*_max free_) = 1 by using a choice function for the next-needed unallocated graph node. This choice is, of course, multiplicative, but other ways of achieving that property are discussed in Supplementary Section SA.2.

### 3.1 Algebra of Binary and Mutual Exclusion State Changes

The expressions […] in square brackets in Eq. 11 for ${\hat{W}}_{r_{2}}{\hat{W}}_{r_{1}}$ need to be restored to normal order, with annihilators *a*_*α*_ to the right of (preceding) creation operators ${\hat{a}}_{\alpha }$. To this end, we need various operator rules for 2 × 2 elementary operators:

(19a) $$\hat{a}=\left(\begin{array}{cc} 0 & 0\\ 1 & 0 \end{array}\right),a=\left(\begin{array}{cc} 0 & 1\\ 0 & 0 \end{array}\right)\text{implies}$$

(19b) $$\hat{a}a=N\equiv \left(\begin{array}{cc} 0 & 0\\ 0 & 1 \end{array}\right),\;\;a\hat{a}=Z\equiv I-N=\left(\begin{array}{cc} 1 & 0\\ 0 & 0 \end{array}\right),\;\text{and}$$

(19c) $$\left[a_{\alpha },{\hat{a}}_{\beta }\right]=\delta_{\alpha \beta }(I_{\alpha }-2N_{\alpha })I\;\;\;\;\text{Alternative for normal form calcs}\;:$$

(19d) $$a_{\alpha }{\hat{a}}_{\beta }={\hat{a}}_{\beta }a_{\alpha }-2\delta_{\alpha \beta }{\hat{a}}_{\alpha }a_{\alpha }+\delta_{\alpha \beta }I_{\alpha }$$

(19e) $$a_{\alpha }{\hat{a}}_{\beta }=\left(1-\delta_{\alpha \beta }\right){\hat{a}}_{\beta }a_{\alpha }+\delta_{\alpha \beta }Z_{\alpha }.$$

Delta functions *δ*_*αβ*_ are by default Kronecker deltas or products thereof, but if *α* indexes a (node, label) pair and the label includes continuous variables, then *δ*_*αβ*_ for continuous variables should receive Dirac delta factors instead so that the composition rule of Eq. 14 is equally valid for discrete and continuous variables. It is important not to use anticommutators for these 0/1-valued random state vectors, even though, in the case *α* = *β*, the foregoing commutation relations are equivalent to anticommutation relations for fermions in quantum mechanics because, in the case *α* ≠ *β*, the corresponding operators commute and therefore do not anticommute.

For edges at least, we will also need the 2 × 2 “erasure” operator:

(20) $$E_{\alpha }=Z_{\alpha }+a_{\alpha }=\left(\begin{array}{cc} 1 & 1\\ 0 & 0 \end{array}\right),$$

which is a projection operator to the *n*_*α*_ = 0 state.

We can enforce a higher-level mutual exclusion (“winner-might-take-all” or “one or zero hot”) logic of binary labels by fiat using axioms

(21) $$a_{i,\lambda }a_{i,\lambda^{\prime }}=0\;{\hat{a}}_{i,\lambda }{\hat{a}}_{i,\lambda^{\prime }}=0\;a_{i,\lambda }{\hat{a}}_{i,\lambda^{\prime }}=\delta_{\lambda \lambda^{\prime }}Y_{i,\lambda^{\prime }}.$$

where $N_{i,\lambda^{\prime }}^{\left(a\right)}$ and *Y*_*i, λ′*_ are diagonal in the number basis and idempotent. This leads to a crucially more constraining version of Eq. 19e in the case of labels

(22) $$a_{j,\lambda }{\hat{a}}_{i,\lambda^{\prime }}=\left(1-\delta_{ij}\right){\hat{a}}_{i,\lambda^{\prime }}a_{j,\lambda }+\delta_{ij}\delta_{\lambda \lambda^{\prime }}Y_{j,\lambda }.$$

Here, operator *Y*_*j, λ*_ has eigenvalue 1 if node *j* is in the undecided state and also is not in the label *λ* state; otherwise, it is 0. The detailed mapping from Eqs 19-22 is discussed in Supplementary Section SA.4.1.

### 3.2 Removal of Hanging Edges

The hanging edge removal variant of graph grammar rule semantics is

(23) $${\hat{W}}_{r}=\frac{1}{C_{r}\;(N_{\max\;\text{free}})}\int d\mu_{r}\;(X)\;\rho_{r}\left(\lambda (X),\lambda^{\prime }(X)\right)\;\;\times \sum_{\langle i_{1},\ldots i_{k}\rangle_{\neq }}E_{\text{cleanup}}(G^{r}){\hat{a}}_{i_{1},\ldots i_{k}}(G^{r\;\text{out}})a_{\;i_{1},\ldots i_{k}}(G^{r\;\text{in}})$$

where, as in Eq. 6,

(24) $$E_{\text{cleanup}}(G^{r\;\text{in}},G^{r\;\text{out}})=\left[\left(\prod_{p\in G_{\text{nodes}}^{r\;\text{in}}\setminus G_{\text{nodes}}^{r\;\text{out}}}\prod_{i\in U}E_{i_{p}\;i}\right)\left(\prod_{p\in G_{\text{nodes}}^{r\;\text{in}}\setminus G_{\text{nodes}}^{r\;\text{out}}}\prod_{i\in U}E_{i\;i_{p}}\right)\right]\;\;{\hat{a}}_{i_{1},\ldots i_{k}}(G^{\prime })={\hat{a}}_{i_{1},\ldots i_{k}}\;(G_{\text{links}}^{\;\;\prime }){\hat{a}}_{i_{1},\ldots i_{k}}\;(G_{\text{nodes}}^{\;\;\;\prime })\equiv \left[\prod_{(s^{\prime },t^{\prime })\in G_{\text{links}}^{\;\prime }}{\hat{a}}_{i_{s^{\prime }}i_{t^{\prime }}}\right]\left[\prod_{v^{\prime }\in G_{\text{nodes}}^{\;\prime }}{\hat{a}}_{i_{v^{\prime }}\lambda_{v^{\prime }}^{\prime }}\right]\;\;a_{i_{1},\ldots i_{k}}(G)=a_{i_{1},\ldots i_{k}}\;(G_{\text{links}})a_{i_{1},\ldots i_{k}}\;(G_{\text{nodes}})\equiv \left[\prod_{(s,t)\in G_{\text{links}}}a_{i_{s}i_{t}}\right]\left[\prod_{v\in G_{\text{nodes}}}a_{i_{v}\lambda_{v}}\right],$$

*A* \ *B* is again the set difference, that is, the subset of *A* not containing members of *B*, and 𝒰 is the universe of object-modeling domain graph nodes.

### 3.3 Index Notation

In order to calculate operator products, we introduce systematic index set notation as follows.

Define *L*_*χ*_, *R*_*χ*_, ℒ_*χ*_, ℛ_*χ*_, for *χ* ∈ {1, 2}:

(25) $$\;\text{lhs nodes}(r_{1})\overset{ℐ}{\mapsto }ℐ\;(G_{\text{nodes}}^{1\;\text{in}})\equiv L_{1}\;\;\;\;\text{rhs nodes}(r_{1})\overset{ℐ}{\mapsto }ℐ\;(G_{\text{nodes}}^{1\;\text{out}})\equiv R_{1}\;\text{lhs nodes}(r_{2})\overset{𝒥}{\mapsto }𝒥\;(G_{\text{nodes}}^{2\;\text{in}})\equiv L_{2}\;\;\;\;\text{rhs nodes}(r_{2})\overset{𝒥}{\mapsto }ℐ\;(G_{\text{nodes}}^{2\;\text{out}})\equiv R_{2};\;\;\text{lhs links}(r_{1})\overset{ℐ}{\mapsto }ℐ\;(G_{\text{links}}^{1\;\text{in}})\equiv {ℒ}_{1}\;\;\;\;\text{rhs links}(r_{1})\overset{ℐ}{\mapsto }ℐ\;(G_{\text{links}}^{1\;\text{out}})\equiv {ℛ}_{1}\;\;\text{lhs links}(r_{2})\overset{𝒥}{\mapsto }𝒥\;(G_{\text{links}}^{2\;\text{in}})\equiv {ℒ}_{2}\;\;\;\;\text{rhs links}(r_{2})\overset{𝒥}{\mapsto }ℐ\;(G_{\text{links}}^{2\;\text{out}})\equiv {ℛ}_{2}.$$

In this notation, the no-edge-cleanup semantics of Eq. 6 becomes (making the parameter integrals implicit now, to limit the notational expansion):

(26) $${\hat{W}}_{r_{\chi }}=\frac{1}{C_{r_{\chi }}\;(N_{\max\;\text{free}})}\rho_{r_{\chi }}\left(\lambda^{\left(\chi \right)},\lambda^{\prime {\;}^{\left(\chi \right)}}\right)\sum_{{ℐ}_{\chi }:L_{\chi }\cup R_{\chi }\overset{1-1}{↪}𝒰}\;\;\times \left[\prod_{(i_{1},i_{2})\in {ℛ}_{\chi }}{\hat{a}}_{i_{1}i_{2}}\right]\left[\prod_{i_{5}\in R_{\chi }}{\hat{a}}_{i_{5}^{{}^{\prime }},\lambda_{{ℐ}^{-1}(i_{5})}^{\left(1\right)}}\right]\left[\prod_{(i_{3},i_{4})\in {ℒ}_{\chi }}a_{i_{3}i_{4}}\right]\left[\prod_{i_{6}\in L_{\chi }}a_{i_{6},\lambda_{{ℐ}^{-1}(i_{6})}^{\left(1\right)}}\right]$$

for *χ* ∈ {1, 2}, where ℐ_*χ*=1_ ≡ ℐ and ℐ_*χ*=2_ ≡ 𝓘. Notation “$\overset{1-1}{↪}$” denotes any one-to-one map from whole of the stated domain into the stated range. Note that the middle square-bracketed terms commute trivially as elementary node and link operators operate in different spaces.

Also in this notation, node maps ℐ and 𝒥 can have overlapping images in 𝒰. This relationship is parameterized by a set *S* (the inverse image of the overlap, under ℐ) and an induced map *h* from *S* into the domain of 𝒥 (from the inverse image of the overlap under ℐ to the inverse image of the overlap under 𝒥):

(27) $$\;S={\text{rhs}}_{1}\;\cap \;h^{-1}\;({\text{lhs}}_{2})\;\;\;\;\;\;\;=G_{\text{nodes}}^{r_{1}\;\text{out}}\;\cap \;h^{-1}\;(G_{\text{nodes}}^{r_{2}\;\text{in}})\;h(S)={\text{lhs}}_{2}\;\cap \;h\;({\text{rhs}}_{1})\;\;\;\;\;\;\;\;=G_{\text{nodes}}^{r_{2}\;\text{in}}\;\cap \;h^{-1}\;(G_{\text{nodes}}^{r_{1}\;\text{out}})\;ℐ(S)=𝒥(h(S))=L_{2}\;\cap \;R_{1}\;\bar{ℐ(S)}=\bar{L_{2}\;\cap \;R_{1}}=\bar{L_{2}}\;\cup \;\bar{R_{1}}.$$

Note also that

(28) $${ℒ}_{\chi }\subseteq \left[L_{\chi }\times L_{\chi }\right]\;\text{and}\;{ℛ}_{\chi }\subseteq \left[R_{\chi }\times R_{\chi }\right]$$

should be preserved inductively by rule firing semantics.

Define 𝒫_*χ*_(*i*_1_, *i*_2_) = a predicate that determines which edges *E*_*i*_1_,*i*_2__ are hanging, if present, and should be deleted, where *χ* ∈ {1, 2}. It may be a predicate function: ${𝒫}_{\chi }[L_{\chi },R_{\chi },\ldots ,G_{\text{links}}^{\chi \;\text{in}},G_{\text{links}}^{\chi \;\text{out}}](i_{1},i_{2})$. Also, *P*^*T*^(*i*_1_, *i*_2_) ≡ *P*(*i*_2_, *i*_1_). We will use one of several equivalent possibilities:

(29) $${𝒫}_{\chi }=\left[\left(L_{\chi }\setminus R_{\chi }\right)\times 𝒰\right]\overset{\text{dual}}{↔}{𝒫}_{\chi }^{⋆}={𝒫}_{\chi }^{T}=\left[𝒰\times \left(L_{\chi }\setminus R_{\chi }\right)\right]$$

As before, 𝒰 = the universe of possible node indices *i*.

### 3.4 Sketch of Commutation Calculation: No Edge Cleanup

The product of two such operators is (omitting for now the integral over parameters *X*)

(30) $${\hat{W}}_{r_{2}}{\hat{W}}_{r_{1}}=\frac{1}{C_{r_{1}}\;(N_{\max\;\text{free}})}\frac{1}{C_{r_{2}}\;(N_{\max\;\text{free}})}\rho_{r_{1}}\left(\lambda^{\left(1\right)},\lambda^{\prime^{\left(1\right)}}\right)\rho_{r_{2}}\left(\lambda^{\left(2\right)},\lambda^{\prime^{\left(2\right)}}\right)\sum_{𝒥:L_{2}\cup R_{2}\overset{1-1}{↪}𝒰}\;\;\times \sum_{ℐ:L_{1}\cup R_{1}\overset{1-1}{↪}𝒰}\left[\prod_{\left(j_{1},j_{2}\right)\in {ℛ}_{2}}{\hat{a}}_{j_{1}j_{2}}\right]\left[\prod_{\left(j_{3},j_{4}\right)\in {ℒ}_{2}}a_{j_{3}j_{4}}\right]\left[\prod_{j_{5}\in R_{2}}{\hat{a}}_{j_{5}^{{}^{\prime }},\lambda_{{𝒥}^{-1}(j_{5})}^{\;(2)}}\right]\;\;\times \left[\prod_{j_{6}\in L_{2}}a_{j_{6},\lambda_{{𝒥}^{-1}(j_{6})}^{\left(2\right)}}\right]\left[\prod_{(i_{1},i_{2})\in {ℛ}_{1}}{\hat{a}}_{i_{1}i_{2}}\right]\left[\prod_{(i_{3},i_{4})\in {ℒ}_{1}}a_{i_{3}i_{4}}\right]\;\;\times \left[\prod_{i_{5}\in R_{1}}{\hat{a}}_{i_{5}^{{}^{\prime }},\lambda_{{ℐ}^{-1}(i_{5})}^{\;(1)}}\right]\left[\prod_{i_{6}\in L_{1}}a_{i_{6},\lambda_{{ℐ}^{-1}(i_{6})}^{\left(1\right)}}\right]$$

Then, we will use the relevant commutation relations to calculate the following:

**Lemma 1.** Let *H*(*S, h*) be the maximal common subgraph of both *G*^*r*_1_ out^ and *G*^*r*_2_ in^, for any given choice of nodes S in *G*^*r*_1_ out^ and 1-1 corresponding nodes h(S) in *G*^*r*_2_ in^. We can restrict S to sets of nodes whose labels match in $G_{\text{nodes}}^{r_{2}\;\text{in}}$ and $G_{\text{nodes}}^{r_{1}\;\text{out}}$. For any such H, we can commute the link operators as follows:

(31) $$\left[\prod_{\left\{(j_{3},j_{4})\in {ℒ}_{2}\right\}}a_{j_{3}j_{4}}\right]\left[\prod_{\left\{(i_{1},i_{2})\in {ℛ}_{1}\right\}}{\hat{a}}_{i_{1}i_{2}}\right]\;\;=\left[\prod_{\{(i_{1},i_{2})\in ℐ\left(G_{\text{links}}^{r_{1}\;\;\text{out}}\setminus H_{\text{links}})\right\}}{\hat{a}}_{i_{1}i_{2}}\right]\left[\prod_{\{(j_{3},j_{4})\in 𝒥\left(G_{\text{links}}^{r_{2}\;\;\text{in}}\setminus h^{-1}\;(H_{\text{links}}))\right\}}a_{j_{3}j_{4}}\right]\;\;\times \left[\prod_{\left\{(j_{7},j_{8})\in ℐ(H_{\text{links}})\equiv {ℒ}_{2}\cap {ℛ}_{1}\right\}}Z_{j_{7}j_{8}}\right]$$

The last factor above implements the edge-checking or link correspondence portion of graph matching between a subgraph H(S, h) of the output graph of rule r_1_ and a corresponding subgraph of the input graph of rule r_2_.

Note that the 1-1 and onto node map $h:H\to \tilde{H}$ preserves edges and labels of labeled subgraphs *H* and $\tilde{H}$ and thus is an isomorphism of labeled subgraphs.

By further calculation and careful interpretation of terms, we arrive at the main result, except limited to the case in which hanging edges are not removed by the rule semantics: for the hanging edge permissive semantics of Eqs 5, 6, or equivalently Eq. 26,

(32) $${\hat{W}}_{G^{r_{2}\;\;\text{in}}\to G^{r_{2}\;\;\text{out}}}{\hat{W}}_{G^{r_{1}\;\;\text{in}}\to G^{r_{1}\;\;\text{out}}}≃\sum_{\begin{array}{c} H\subseteq G^{r_{1}\;\;\text{out}}≃\tilde{H}\subseteq G^{r_{2}\;\;\text{in}}\\ \;\;|\;\text{edge}-\text{maximal} \end{array}}\sum_{h:\;H\overset{1-1}{↪}\tilde{H}}{\hat{W}}_{G^{r_{1}\;\;\text{in}}\dot{\cup }\left(G^{r_{2}\;\;\text{in}}\setminus \tilde{H}\right)\;\underset{h}{\to }\;G^{r_{2}\;\;\text{out}}\dot{\cup }(G^{r_{1}\;\;\text{out}}\setminus H)}$$

In more detail, the summand graph rewrite rule is then defined by Theorem 1. Under the definitions of the compound label graphs in Eqs 34, 35, one can write the graph rewrite rule algebra as announced in Section 2.4.

**Theorem 1.** For the hanging edge-permissive semantics of Eqs 5, 6 or equivalently Eq. 26 and assuming multiplicative normalization C_r_, then

(33) $${\hat{W}}_{G^{r_{2}\;\;in}\to G^{r_{2}\;\;out}}{\hat{W}}_{G^{r_{1}\;\;in}\to G^{r_{1}\;\;out}}≃\sum_{\begin{array}{c} H\subseteq G^{r_{1}\;\;\text{out}}≃\tilde{H}\subseteq G^{r_{2}\;\;\text{in}}\\ \;\;|\;\text{edge}-\text{maximal} \end{array}}\sum_{h:\;H\overset{1-1}{↪}\tilde{H}}{\hat{W}}_{G^{1;2\;\;in}\left(\tilde{H}\right)\;\underset{h}{\to }\;G^{1;2\;\;out}(H)}$$

where the compound labeled graphs *G*^1;2 in^ ($\tilde{H}$) and G^1;2 out^(H) are defined by

(34) $$G_{nodes}^{1;2\;in}\left({\tilde{H}}_{nodes}\right)=G_{nodes}^{r_{1}\;in}\dot{\cup }\left(G_{nodes}^{r_{2}\;\;in}\setminus {\tilde{H}}_{nodes}\right)\;\;\;\;\;G_{nodes}^{1;2\;out}\;(H_{nodes})=G_{nodes}^{r_{2}\;\;out}\dot{\cup }\left(G_{nodes}^{r_{1}\;\;out}\setminus H_{nodes}\right)\;\;\equiv G_{nodes}^{r_{1}\;in}\;\cup \;h^{-1⋆}\left(G_{nodes}^{r_{2}\;in}\setminus {\tilde{H}}_{nodes}\right)\;\;\;\;\;\;\;\;\equiv G_{nodes}^{r_{2}\;\;out}\;\cup \;h^{⋆}\left(G_{nodes}^{r_{1}\;\;out}\setminus H_{nodes}\right)\;G_{links}^{1;2\;in}\left({\tilde{H}}_{nodes}\right)=G_{links}^{r_{1}\;in}\;\cup \;h^{-1⋆}\left(G_{links}^{r_{2}\;in}\setminus {\tilde{H}}_{links}\right)\;\;\;\;\;G_{links}^{1;2\;out}\;(H_{links})=G_{links}^{r_{2}\;\;out}\;\cup \;h^{⋆}\left(G_{links}^{r_{1}\;\;out}\setminus H_{links}\right)$$

and their label overlaps K_1;2_ are defined by

(35) $$\;K_{a}=G_{nodes}^{r_{a}\;in}\;\cap \;G_{nodes}^{r_{a}\;\;out}\;K_{1;2}=(K_{1}\setminus H_{nodes})\;\cup \;h^{-1}\left(K_{2}\setminus {\tilde{H}}_{nodes}\right)\cup \left(K_{1}\;\cup \;h^{-1\;⋆}\;(K_{2})\right).$$

The coefficients in Eq. 33 are all nonnegative integers (as the same graph grammar rule could arise several times by different means). Rate factors ρ multiply with parameter substitution, as in Eq. 14. Here, symbol $\overset{\cdot }{\cup }$ denotes disjoint union, and *h*^★^: *G*^*r*_1_ *out*^ → *G*^1;2 *out*^ extends $h:H\subseteq G^{r_{1}\;out}\to \tilde{H}\subseteq G^{r_{2}\;in}$ by remapping the nodes of *G*^*r*_1_^ along h if possible and to the disjoint union nodes if not, preserving all possible links except those in H_*links*_, likewise for $h^{-1}:\tilde{H}\subseteq G^{r_{2}\;in}\to H\subseteq G^{r_{1}\;out}$ and *h*^−1★^: *G*^*r*_2_ in^ → *G*^1;2 in^.

Proof: The proof of this theorem is provided in Supplementary Material SA, with Theorem 1. It follows the proof sketch above but is written out in detail.

Note that Eqs 34, 35 reflect a time-reversal *L* ↔ *R* duality. Examples of graph numbering and disjoint union $\overset{\cdot }{\cup }$ are given in Section 3.7. We now derive a series of corollaries presented only here, not in the detailed calculation sections.

**Corollary 1.** There is an algebraic reduction of operator products to sums, similar to Theorem 1, which applies to the W_r_ operators that subtract diagonal operators from ${\hat{W}}_{r}$ to conserve probability as in Eq. 1, except that the coefficients can be any integer.

Proof: Note that substituting *Z*_*α*_ = *I*_*α*_ − *N*_*α*_ in each elementary operator in Eq. 54 of Supplementary Section SA.1 and distributing multiplication over addition, yields an integer-weighted sum of operators of the form of Eq. 53 of Supplementary Section SA.1 or equivalently Eq. 5. Therefore, *W*_*r*_2__*W*_*r*_2__ is equivalent to a sum of ${\hat{W}}_{s}$ operators for a set of labeled graph grammar rules indexed by *s*. As *W*_*r*_2__ preserves probability, **1** · *W*_*r*_2__*W*_*r*_1__ = **0** · *W*_*r*_1__ = **0**. We can therefore subtract zero in the form of diag (**1** · *W*_*r*_2__*W*_*r*_1__), applied term by term with the same sum of graph grammar rules substituted in for *W*_*r*_2__*W*_*r*_1__, and find that *W*_*r*_2__*W*_*r*_2__ is equivalent to a sum of full $W={\hat{W}}_{s}-\text{diag}(1\cdot {\hat{W}}_{s})$ operators for a set of labeled graph grammar rules indexed by *s*.

**Corollary 2.** There is an algebraic reduction of commutators of labeled graph grammar rule state-change operators ${\hat{W}}_{r}$ to sums of the same form, similar to Theorem 1, with integer coefficients and cancellation of $H=\emptyset =\tilde{H}$ summands:

(36) $$\left[{\hat{W}}_{G^{r_{2}\;\;in}\to G^{r_{2}\;\;out}},{\hat{W}}_{G^{r_{1}\;\;in}\to G^{r_{1}\;out}}\right]≃\sum_{\begin{array}{c} H\subseteq G^{r_{1}\;\text{out}}≃\tilde{H}\subseteq G^{r_{2}\;\text{in}}\\ \;H\neq \emptyset \;\;\wedge \;\;\text{edge}-\text{maximal} \end{array}}\sum_{h:\;H\overset{1-1}{↪}\tilde{H}}{\hat{W}}_{G^{1;2\;in}\left(\tilde{H}\right)\;\underset{h}{\to }\;G^{1;2\;out}(H)}\;\;-\sum_{\begin{array}{c} H\subseteq G^{r_{2}\;\text{out}}≃\tilde{H}\subseteq G^{r_{1}\;\text{out}}\\ \;H\neq \emptyset \;\;\wedge \;\;\text{edge}-\text{maximal} \end{array}}\sum_{h:\;H\overset{1-1}{↪}\tilde{H}}{\hat{W}}_{G^{2;1\;in}\left(\tilde{H}\right)\;\underset{h}{\to }\;G^{2;1\;out}(H)}$$

Also, there is a similar algebraic reduction of commutators of labeled graph grammar rule full operator *W*_*r*_ commutators to sums of the same form, with integer coefficients.

Proof: As in Corollary 1, but with extra minus signs on some of the rule operators. Cancellation of $H=\emptyset =\tilde{H}$ summands follows from the *r*_1_ → *r*_2_ symmetric definitions of *G*^1;2 in^ and *G*^1;2 out^ in Eq. 34 in that special case.

**Corollary 3.** There exists (as exhibited in the proof of Theorem 1) a constructive mapping from the graph rewrite rule operator algebra semantics to the elementary bitwise operator algebras of Supplementary Section SA.3.1. Because it depends on an index allocation scheme which can be done in many ways, this mapping is not unique.

**Corollary 4.** One particular subgraph that always contributes to the product is $H=\emptyset =\tilde{H}$, the empty graph. Its contribution always cancels out of the commutator $[{\hat{W}}_{r_{2}},{\hat{W}}_{r_{1}}]={\hat{W}}_{r_{2}}{\hat{W}}_{r_{1}}-{\hat{W}}_{r_{1}}{\hat{W}}_{r_{2}}$ because *H* = ∅ and then nothing is shared between the two rule firings so their order does not matter.

### 3.5 Sketch of Commutation Calculation: With Edge Cleanup

We now turn to the hanging edge cleanup semantics and prove (Theorem 2) that the same algebra as in Theorem 1 and Eqs 34 and 35, and 33 still applies.

An elaboration of rule operators ${\hat{W}}_{r}$ can clean up hanging edges that may otherwise be left behind by a rule firing:

(37) $${\hat{W}}_{r}^{\text{cleaned}}=\left(\prod_{k_{1}\in L_{r}\setminus R_{r}}\prod_{k_{2}\in 𝒰}E_{k_{1}k_{2}}E_{k_{2}k_{1}}\right){\hat{W}}_{r}^{\text{bare}}\;\;≃\left(\prod_{(k_{1},k_{2})\in 𝒮}E_{k_{1}k_{2}}\right)\left(\prod_{(k_{1},k_{2})\in 𝒮}E_{k_{2}k_{1}}\right){\hat{W}}_{r}^{\text{bare}}$$

where 𝒮 is the set of indices specified by

(38) $$𝒮=[(L_{r}\setminus R_{r})\times {𝒰}_{A^{⋆}}]$$

where 𝒰_A*_ = all node indices that have ever been allocated in a memory block, hence all memory-live node indices, and 𝒰 = the whole universe of node indices, so that 𝒰_A*_ ⊆ 𝒰.

The semantics is now

(39) $${\hat{W}}_{r_{\chi }}=\frac{1}{C_{r_{\chi }}\;(N_{\max\;\text{free}})}\int d\mu_{r_{\chi }}(X)\;\rho_{r_{\chi }}\left(\lambda [X],\lambda^{\prime }[X]\right)\sum_{{ℐ}_{\chi }:L_{\chi }\cup R_{\chi }\overset{1-1}{↪}𝒰}\left[\left(\prod_{\left(i^{\prime },i\right)\in {𝒫}_{\chi }}E_{i^{\prime }\;i}\right)\left(\prod_{\left(\hat{i},{\hat{i}}^{\prime }\right)\in {𝒫}_{\chi }^{*}}E_{\hat{i}\;{\hat{i}}^{\prime }}\right)\right]\;\;\times \left[\prod_{(i_{1},i_{2})\in {ℛ}_{\chi }}{\hat{a}}_{i_{1}i_{2}}\right]\left[\prod_{(i_{3},i_{4})\in {ℒ}_{\chi }}a_{i_{3}i_{4}}\right]\left[\prod_{i_{5}\in R_{\chi }}{\hat{a}}_{i_{5},\lambda_{{ℐ}_{\chi }^{-1}(i_{5})}^{\prime }}\right]\left[\prod_{i_{6}\in L_{\chi }}a_{i_{6},\lambda_{{ℐ}_{\chi }^{-1}(i_{6})}}\right].$$

We now work to replace the product of *E*_*ij*_ factors above with the exponential of a sum:

(40) $$E_{\alpha }=Z_{\alpha }+a_{\alpha }=I_{\alpha }+(a_{\alpha }N_{\alpha })=I_{\alpha }+W_{\alpha \to \emptyset }$$

Defining *ϵ* = *τ*/*m*, we will see that

(41) $$\text{exp}\left(\tau \sum_{\alpha \in 𝒮}W_{\alpha \to \emptyset }\right)=\underset{m\to +\infty ,∊->0^{+}}{\text{lim}}{\left(\prod_{\alpha \in 𝒮}(I+∊W_{\alpha \to \emptyset }\right)}^{m}.$$

and we will compute that therefore asymptotically as *τ* = *ρ*_erase_*t* → +∞,

(42) $$\text{exp}\left(\tau \sum_{\alpha \in 𝒮}W_{\alpha \to \emptyset }\right)=\prod_{\alpha \in 𝒮}E_{\alpha }.$$

So, complete erasure is the limiting behavior of this edge-by-edge stochastic erasure process, and it can be achieved simply by taking the limit *ρ*_erase_ → +∞.

Now, we apply these calculations to the actual hanging edge erasure operator:

(43) $$\text{exp}\left(\tau \sum_{(i_{1},i_{2})\in 𝒮}W_{(i_{1},i_{2})\to \emptyset }\right)=\text{exp}\left(\tau \sum_{(i_{1},i_{2})\in 𝒮}(E_{i_{1},i_{2}}-I_{i_{1},i_{2}})N_{i_{2}}Z_{i_{1}}\right)$$

Here, the node operator *Z*_*i*_ checks for unallocated nodes *i* with no label.

Then, asymptotically as *τ* = *ρ*_erase_*t* → +∞,

(44) $$\text{exp}\left(\tau \sum_{\alpha \in 𝒮}W_{\alpha \to \emptyset }\right)=\prod_{(i,j)\in 𝒮}E_{i,j}N_{j}Z_{i}≃\prod_{(i,j)\in 𝒫}E_{i,j}.$$

So again, we get the product of forward edge erasures by an incremental process of deletion, run for a long effective time *τ*.

In Eq. 37,

(45) $${\hat{W}}^{\text{cleaned}}=\left(\prod_{(k_{1},k_{2})\in 𝒮}E_{k_{1}k_{2}}E_{k_{2}k_{1}}\right){\hat{W}}^{\text{bare}}\;\;=\underset{n\to +\infty ,∊\to 0^{+}}{\text{lim}}{\left[I+∊\sum_{(k_{1},k_{2})\in 𝒮}(a_{k_{1},k_{2}}N_{k_{1},k_{2}})N_{k_{2}}Z_{k_{1}}\right]}^{n}\;\;\times {\left[I+∊\sum_{(k_{1},k_{2})\in 𝒮}(a_{k_{2},k_{1}}N_{k_{2},k_{1}})N_{k_{1}}Z_{k_{2}}\right]}^{n}{\hat{W}}^{\text{bare}}$$

The core calculation within ${\hat{W}}_{r_{2}}^{\text{cleaned}}\cdot {\hat{W}}_{r_{1}}^{\text{cleaned}}$ is thus

(46) $${\hat{W}}_{r_{2}}^{\text{bare}}\left[∊\sum_{(k_{1},k_{2})\in 𝒮}(a_{k_{1},k_{2}}-N_{k_{1},k_{2}})N_{k_{2}}Z_{k_{1}}\right]\;\;=\frac{∊}{C_{r_{2}}}\sum_{ℐ}\sum_{(k_{1},k_{2})\in 𝒮}\left[\prod_{(i_{1},i_{2})\in {ℛ}_{2}}{\hat{a}}_{i_{1}i_{2}}\right]\left[\prod_{(i_{3},i_{4})\in {ℒ}_{2}}a_{i_{3}i_{4}}\right](a_{k_{1},k_{2}}-N_{k_{1},k_{2}})\;\;\times \left[\prod_{i_{5}\in R_{2}}{\hat{a}}_{i_{5},\lambda_{{ℐ}^{-1}(i_{5})}}\right]\left[\prod_{i_{6}\in L_{2}}a_{i_{6},\lambda_{{ℐ}^{-1}(i_{6})}}\right]N_{k_{2}}Z_{k_{1}}$$

By operator algebra calculation, we find

(47) [∏(i1,i2)∈ℛ2a^i1i2][∏(i3,i4)∈ℒ2ai3i4](ak1,k2Nk1,k2)={−[∏(i1,i2)∈ℛ2a^i1i2][∏(i3,i4)∈ℒ2ai3i4]if(k1,k2)∈ℒ2Nk1,k2[∏(i1,i2)∈ℛ2a^i1i2][∏(i3,i4)∈ℒ2ai3i4]if(k1,k2)∈ℛ2∖ℒ2(ak1,k2Nk1,k2)[∏(i1,i2)∈ℛ2a^i1i2][∏(i3,i4)∈ℒ2ai3i4]if(k1,k2)∈ℛ¯2∩ℒ¯2.}

Further arguments in the detailed calculation section will show that all surviving terms behave as in the third line of Eq. 47, and the factor of *a* − *N* to the right of the second rule firing simply joins the infinite supply of such factors to its left.

Intuitively, this means that hanging edges can be eliminated nonspecifically by an overactive syntax-checking process rather than surgically in a way that depends on the details of each rule firing because the assumed form of the graph rewrite rules does not recognize or respond to hanging edges; all edges are verified to have two vertices before a rule can fire. As an aside, this explanation would not remain valid if the semantics were changed to allow things like the nonconforming operator *W*_(*i*_1_,*i*_2_)→∅_ above, so as to allow hanging edges as part of the normal graph grammar operation. Then, a more complex algebraic operator equation might result.

Thus, we find no change to the algebraic formula of Theorem 1 for the hanging edge removal semantics.

**Theorem 2.** For the hanging edges removal semantics of Eqs. 23, 24, or equivalently Eq. 39, assuming finiteness of rules, index allocation blocks, and number of rule firings, and assuming multiplicative normalization *C_r_*, then

(48) $${\hat{W}}_{G^{r_{2}\;\;\text{in}}\to G^{r_{2}\;\;\text{out}}}{\hat{W}}_{G^{r_{1}\;\;\text{in}}\to G^{r_{1}\;\;\text{out}}}≃\sum_{\begin{array}{c} H\subseteq G^{r_{1}\;\;\text{out}}≃\tilde{H}\subseteq G^{r_{2}\;\;\text{in}}\\ |\;\text{edge}-\text{maximal} \end{array}}\sum_{h:\;H\overset{1-1}{↪}\tilde{H}}{\hat{W}}_{G^{1;2\;\;\text{in}}\left(\tilde{H}\right)\;\underset{h}{\to }\;G^{1;2\;\;\text{out}}(H)}$$

where the compound labeled graphs $G^{1;2\;\text{in}}(\tilde{H})$ and *G*^1;2 out^(*H*), and their label overlaps *K*_1;2_ are defined by Eqs 34, 35 in Theorem 1. The coefficients in this expression are all nonnegative integers (as the same graph grammar rule could arise several times by different means). Rate factors *ρ* multiply with parameter substitution, as in Eq. 14.

Proof: The proof of this theorem is provided in Supplementary Material SA, with Theorem 2. It follows the proof sketch above but is written out in detail.

We now derive another series of corollaries presented only here, not in the detailed calculation section.

**Corollary 5.** There is an algebraic reduction of operator products to sums, similar to Theorem 2, that applies to the *W_r_* operators that subtract diagonal operators from ${\hat{W}}_{r}$ to conserve probability, except that the coefficients can be any integer.

Proof: Exactly as for Corollary 1.

**Corollary 6.** There is an algebraic reduction of commutators of labeled graph grammar rule state-change operators ${\hat{W}}_{r}$ to sums of the same form, similar to Theorem 2, with integer coefficients. Also, there is a similar algebraic reduction of commutators of labeled graph grammar rule full operator W_r_ commutators to sums of the same form, with integer coefficients.

Proof: As in Corollary 5 or 1, but with extra minus signs on some of the rule operators.

**Corollary 7.** There exists (as exhibited in the proofs of Theorems 1 and 2) a constructive mapping from the graph rewrite rule operator algebra semantics to the elementary bitwise operator algebras of Supplementary Section SA.3.1. As it depends on an index allocation scheme that can be done in many ways, this mapping is not unique.

**Corollary 8.** One particular subgraph that always contributes to the product is $H=\emptyset =\tilde{H}$, the empty graph. Its contribution always cancels out of the commutator $[{\hat{W}}_{r_{2}},{\hat{W}}_{r_{1}}]={\hat{W}}_{r_{2}}{\hat{W}}_{r_{1}}-{\hat{W}}_{r_{1}}{\hat{W}}_{r_{2}}$ because nothing is shared between the two rule firings so their order does not matter.

We note here that a previous attempt to prove Theorem 2 directly using the large product of *E* operators and 𝒫, *L, R*, ℒ, *R*, among others, by Boolean logic ran aground in notational complexity. The method used here, with the exponential of a sum of *E* − *I* operators, seems more tractable.

### 3.6 Application to ODEs and Dynamical Graph Grammars

In Section 2.2.1, we showed that a graph grammar rule that expresses a differential equation by not adding or removing any graph edges and by changing only the node labels, not the presence or absence of graph nodes, can be expressed within the general framework by applying Theorems 1 and 2 using a particular form of rule rate function *ρ* which, however, may take values of either sign. All steps of Theorems 1 and 2 remain valid. The signs of the commutator-induced coefficients that multiply the rate functions *ρ* remain as stated in these theorems and their corollaries.

The only change is that when the time to derive a simulation algorithm for these semantics comes, the functions *ρ*_*r*_ for differential equation rules cannot be interpreted as propensities (unnormalized probabilities per unit time) because they can be negative. That is all right because Mjolsness (2013) derived a separate kind of algorithm for stochastic parameterized grammars that contain such rules, calling an ODE solver as a subroutine. Of course, Section 2.2.1 together with Eq. 49 below suggests another algorithm under a limiting procedure for small but discrete changes in parameter values.

Thus, we show the following:

**Proposition 1.** Theorems 1 and 2 extend to rules that express differential equations by way of semantics incorporating Dirac delta functions as in Eq. 9.

For example, we will see in Supplementary Material SB.3 that the operators [𝒪_DE 2_, 𝒪_DE 1_] of two differential equations for the same variables *d**x**/dt* = ***v***_1_(***x***) and *d**x**/dt* = ***v***_2_(***x***) have a commutator 𝒪_DE [2, 1]_ equivalent to a third differential equation *d**x**/dt* = ***v***_[2, 1]_(***x***) ≡ (***v***_1_ · grad_***x***_)***v***_2_(***x***) − (***v***_2_ · grad_***x***_)***v***_1_(***x***). In the same section, we will use the notation of Theorem 1 to exhibit the commutator of an ODE rule and a (non-ODE) SPG rule.

Alternatively to Eq. 9, one could eschew continuous parameters until the very end of a calculation by taking continuous “motion” of each real-valued parameter component *x*_*a*_ under an ODE to consist of many small discrete uniform-sized steps ±Δ*x*, with Δ*x* > 0, with the sign chosen to be that of *v* in each component, and each step having a continuous-time propensity to occur given by ∣*v*(***x***)∣. Then, after integration by parts and assuming suitable boundary conditions can be imposed on ***v***,

(49) $${\hat{W}}_{\text{ODE discr}\;r}=\sum_{x,a}\frac{|v_{a}(x)|}{\Delta x}\sum_{\langle i_{1},\ldots i_{k}\rangle_{\neq }}[{\hat{a}}_{i_{1},\ldots i_{k}}\;(G^{r}\;(x+\text{sign}\;(v_{a}(x))\Delta xe_{a}))a_{i_{1},\ldots i_{k}}\;(G^{r}\;(x))\;\;-{\hat{a}}_{i_{1},\ldots i_{k}}\;(G^{r}\;(x))a_{i_{1},\ldots i_{k}}\;(G^{r}\;(x))]$$

where ***e***_*a*_ is the unit vector along axis *a* of the local parameter vector space containing ***x***. On timescales Δ*t* ≪ Δ*x*/max_*a*_∣*v_a_* (***x***)∣, parameter jumps occur one at a time and add up in the expected manner. Again, one would take a parametric limit, this time in the limit Δ*x* → 0. This approach has the advantage of non-negative *ρ* functions and, thus, a probabilistic interpretation of the rule operator.

By either of these means, mixed stochastic graph dynamics and differential equation dynamics can be approximated arbitrarily closely by operators of graph grammar dynamics of the algebraic form we have assumed. Eq. 49 also hints at a different family of stochastic simulation algorithms, which may or may not lead to something efficient. Alternatively, as in Eq. 9 and Proposition 1, one can simply admit Dirac delta functions into the allowed expressions for *ρ*_*r*_ and selected commutators, and no parametric limit is needed; this will be our preferred approach.

### 3.7 Examples

Several biological models have been formulated in terms of structural rewrite rules for graphs and cell complexes (Mjolsness et al., 1991; Spicher and Michel, 2007; Giavitto and Spicher, 2008; Lane, 2015) and the literature on L-systems, all reviewed from the present operator algebra point of view in Mjolsness (2019a).

Here, we will take as a working example a highly simplified stochastic parameterized graph grammar (SPGG) for microtubule dynamics, including treadmilling, bundling/zippering, and katanin-mediated severing in cytoskeleton dynamics as it appears in current plant biology.

#### 3.7.1 MT Stochastic Graph Grammar

A diagrammatic presentation of a small subset of a plant cortical microtubule (MT) graph grammar, with subscripts for the rule-local arbitrary but consistent numbering of vertices in left- and right-hand side graphs of each rule, is shown below. These rules and calculations are a subset of those presented in Supplementary Material SB. Discrete parameters will include a four-valued categorical label *s* ∈ {*internal, grow_end, retract_end, junct*} (or *s* ∈ {◦, ●, ■, ▲}) for status as interior segment, growth-capable end segment, retraction-capable end segment, or bundling junction segment, respectively: 
(50)

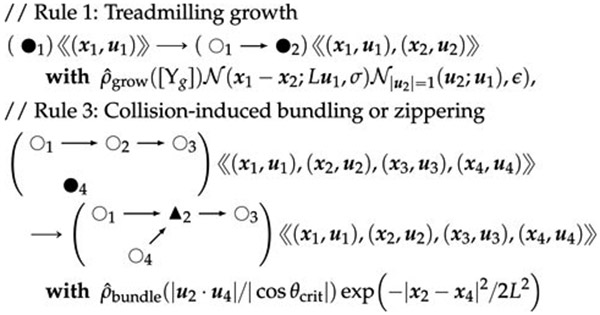



Here, *Y*_*g*_ is a diffusible MT growth factor such as tubulin itself or a catalyst or regulator of tubulin polymerization and/or nucleation, such as (perhaps) XMAP215 (Hamant et al., 2019), and *Y*_*r*_ plays the same role in catastrophe/retraction.

In working out the commutators, we will drop the propensity functions *ρ*, but they just multiply the results with appropriate variable identifications.

Further MT rules are provided in Supplementary Section SB.1.

#### 3.7.2 Example MT Commutator Calculation

The commutator calculations for this minimal MT graph grammar’s Lie algebra can be outlined as follows:

$\left[{\hat{W}}_{3},{\hat{W}}_{1}\right]$:

${\hat{W}}_{3}\cdot {\hat{W}}_{1}$: shared same-label vertex sets run over by *H* and their mappings under *h* are ∅; {(1↦1′)}; {(1↦2′)}; {(1↦3′)}; {(1↦1′), (2↦4′)}; {(1↦2′) (2↦4′)}; {(1↦3′), (2↦4′)}.

${\hat{W}}_{1}\cdot {\hat{W}}_{3}$: shared same-label vertex sets run over by *H* and their mappings under *h* are ∅.

*H* = ∅ always cancels in the commutator:

(51)

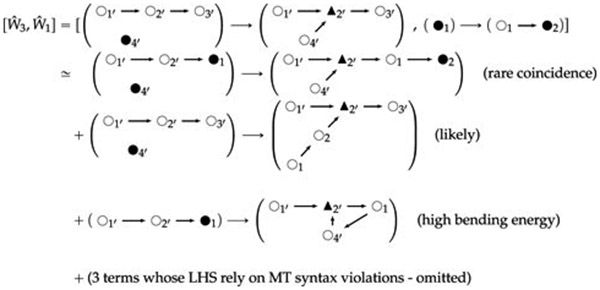



The reason the second line above involves a “rare coincidence” is that its left-hand side represents a collision of two long MTs very near to the growing end of both, assuming the MTs are generically quite long and thus have many internal nodes (open circles). Likewise, the fourth line requires a high bending energy (can thus be disfavored in a more detailed model) because of the loop of three small MT segments, two interior and one junction, in the RHS graph.

Further commutators are calculated in Theorems 1 and 2 and Proposition 1 in Supplementary Section SB.

For the restricted case in which one of the operators is a diagonal “observable,” a rule commutator calculation has been exhibited independently in the “double pushout” formalism (Section 4.2) for a particular set of basic biochemical binding/unbinding rules expressed in Kappa (Behr et al., 2020). The general combinatorial formula of Theorems 1 and 2 and the extension of Proposition 1 remain unique as far as we know.

The special case in which no graph edges are present, only graph nodes, corresponds to a well-mixed stochastic chemical reaction network. The commutation relations for such models are calculated in Supplementary Section SC, in the conventional representation in which all particles of a given type lose their identity and only their population numbers matter.

#### 3.7.3 Actin Cytoskeleton Stochastic Graph Grammar Examples

Actin filament polymerization and depolymerization rules can be analogous to those for MTs. Branching occurs in a different way than the bundling rule for MTs, as for example in this two-dimensional rule:

(52)

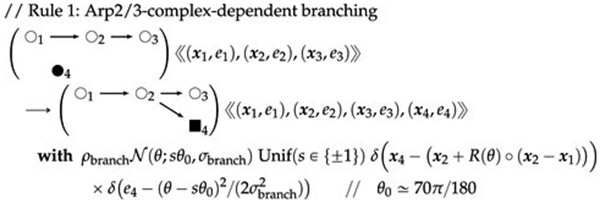



Here, O_2_ represents actin or short polymers of actin (which have a sense of directionality), ● represents the Arp2/3 complex in solution, and ■ represents the Arp2/3 complex bound to actin and can serve as the nucleation site for a new actin filament. Also, the *e* parameters measure biomechanical energy owing to geometry, which can drive mechanics using differential equation rules. A simple prototype model of this sort has been simulated using the software of Yosiphon (2009).

In fact, MTs also have branching nucleation dynamics facilitated by other molecular players such as the augmin complex.

Other actin grammar rules, including polymerization-driven growth, can be modeled in a very similar manner to MT rules. For example, both kinds of filaments undergo catalyzed severing. Growing actin filaments may also acquire an end-cap, preventing further growth.

#### 3.7.4 Related Kinds of Rewrite Rules

We have analyzed the semantics and given examples of stochastic parameterized graph grammar (SPGG) models.

Mjolsness and Yosiphon (2006) demonstrated how to use integer-valued Object ID (OID) parameters to encode such graph grammars within stochastic parameterized grammars (SPG) comprising parameter-bearing stochastic rewrite rules with operator algebra semantics. This reduction requires the use or dynamical emulation of a source of novel OIDs. Because the reverse inclusion is trivial, SLGGs, SPGGs, and SPGs are essentially different syntactic presentations for the same semantics; SPGGs may be easier to write since the OID encoding step is unnecessary.

Nevertheless, Mjolsness and Yosiphon (2006) showed how to add to SPG rules with ordinary and/or stochastic differential equation syntax and differential operator semantics, obtaining “dynamical grammars” (DGs). DGs can be a continuum limit (in label space and in time) of SPGs. If we allow differential equation rules and stochastic parameterized graph grammar rules, we arrive at dynamical graph grammars (DGGs), as defined here and the subject of Proposition 1.

Many other notational conveniences are possible while maintaining or generalizing the operator algebra semantics.

#### 3.7.5 Cell Complex Rewrite Rules

In Mjolsness (2019a), the operator algebra semantics for a labeled graph rewrite rule is generalized in several ways. One of these generalizations is to cell complexes (each of some maximal dimension *d*), which have been applied to developmental modeling (Spicher and Michel, 2007; Lane, 2015). Mjolsness (2019a) also provided a constructive implementation mapping from the generalized rewrite rules back to graph grammar rewrite rules. In principle, the graph grammar operator algebra of our Theorems 1 and 2 apply to these generalized settings—but whether the sum of graph grammar operators resulting from a higher-level product is also a sum of higher-level rewrite rules or not remains to be worked out.

Here, we point out a useful special case for cell complex dynamics: if a graph can be locally embedded in *d* dimensions (i.e., in *d* dimensional manifolds with $R^{d}$ as the usual case) in such a way that it becomes a Voronoi diagram or a power diagram (weighted Voronoi diagram), then its label set can be augmented by the resulting node positions, and, more importantly, there is a dual *d*-dimensional cell complex consisting of the boundaries at equal distance (in the Voronoi case) from two or more graph node positions, together with the *d*-dimensional single-node cells they bound. Then, local graph grammar rewrite rules will generically result in local updates to the embedding and the dual cell complex, inducing local cell complex changes describable as rewrite rules.

As a final point of discussion, in the Lie group theory, the Lie algebra is related to the curvature tensor of a group-invariant metric. Likewise, in differentiable manifolds, commutators of covariant derivatives are related to the manifold curvature tensor. The Lie algebras discussed here are generically in a much higher dimension but, in some cases, may also relate to geometric and/or topological structures.

## 4 DISCUSSION

### 4.1 Conclusion

We have computed the product and commutator for any two stochastic parameterized graph rewrite rule operators in a stochastic graph grammar possessing operator algebra semantics, in structural (graph-expressed, combinatorial) form. In this form, the product of the state-changing portions (off-diagonal in the number basis) of two graph rewrite rule operators is a sum, with nonnegative integer coefficients, of other such operators. Non-negative real-valued rate multipliers are also carried along expectedly. The product of the full-graph rewrite rule operators and the commutator of off-diagonal or full-rule operators are likewise expressed as a sum with integer-valued weights of other full-graph rewrite rule operators. The algebra can also be applied to rewrite rules that bear ordinary differential equations for real-valued node parameters. The results are expressed in Theorem 1 and its corollaries for the case of semantics in which hanging edges are left behind and Theorem 2 and its corollaries for the case in which they are not. Proposition 1 demonstrates the application to the differential equation bearing rules. The algebra can be computed explicitly.

There is also a computer-implementable constructive mapping from the resulting graph rewrite rule algebra to many elementary two-state creation/annihilation operators. Because the algebra is expressed in the present work entirely in terms of identities relating to graph rewrite rule operators (up to equivalence) rather than more general expressions built from the underlying elementary two-state creation/annihilation operators, Theorems 1 and 2 are a substantial improvement in utility and perspicuity over the corresponding Propositions 1 and 2 in Mjolsness (2019a). Here, the operator algebra of graph rewrite rules is “lifted” from the concrete level of creation/annihilation operators on elementary binary random variables to the more abstract and structural level of well-formed labeled graph rewrite rules.

As a clarifying test case, the resulting graph-grammar level algebra was applied to an elementary example inspired by the dynamics of cortical microtubules in plant cells, one of many structure-changing dynamical systems in biophysics and other sciences that could be amenable to modeling by stochastic parameterized graph grammars.

### 4.2 Related Work

The present line of development for operator algebra semantics of chemical reaction networks and graph grammars began with expressivity studies (Mjolsness, 2005; Mjolsness and Yosiphon, 2006), including suitable measure spaces for a probabilistic foundation, followed by a combined SPG and DGG implementation (Yosiphon, 2009), which was applied to growing plant root models with cell division (Mjolsness, 2013), a direct graph semantics without object ID encoding (Mjolsness, 2010), a systematic derivation of stochastic simulation algorithms including differential equations (Mjolsness, 2013), the existence proof for rule product and commutator reduction in (Mjolsness, 2019a), and the calculations reported herein.

The larger context is diverse and includes L-systems (which generate tree-structured graphs without loops) and their generalizations, such as differential L-systems (Prusinkiewicz et al., 1993) and stochastic L-systems (Eichhorst and Walter, 1990; Cieslak and Prusinkiewicz, 2019). The earlier reference (Eichhorst and Walter, 1990) is related to Cieslak and Prusinkiewicz (2019) in part by being applied to computer science rather than biology and by projecting out stochastic event waiting times as described, for example, in Mjolsness and Yosiphon (2006) (Section 3.8), and by the Gillespie algorithm of Cieslak and Prusinkiewicz (2019) is just one possible sampling algorithm for an operator algebra semantics as derived, for example, in Mjolsness (2013). Further context also includes grammar-like “connectionist” models for biological development (Mjolsness et al., 1991) and plant developmental models incorporating cell division (Jönsson et al., 2006; Smith et al., 2006). These include some kind of dynamic graph topology as part of the dynamical system to be modeled. Investigation of more formalized computer support for variable-structure developmental models based on topological cell complexes is shown in Spicher and Michel (2007) and Lane (2015).

Independently, cytoskeletal modeling simulators have been developed, including algorithmic provision for changing topology of filament networks due to, for example, dynamic crosslinking and/or bundling of microtubule or actin fibers, which necessarily change the graph topology that influences further biomechanical dynamics in both microtubule and actin fibers (Nedelec and Dietrich, 2007; Popov et al., 2016; Belmonte et al., 2017; Kim et al., 2022). In such dynamic cytoskeleton codes, there comes a moment when the structure of the graph changes, for example, as a consequence of some molecular binding or unbinding event. At that moment, the problem of biomechanics changes locally but with potentially global consequences. So, it may be important to explore a more systematic formalization, such as the present operator algebra, of local structure-changing dynamics interacting with differential equation dynamics. Improvements in both algorithms and analyses may result.

There is an alternative category-theory-based approach to graph grammar semantics based on single or double pushout (DPO) commutative diagrams rather than operator algebras and a collection of “independence” conditions for two successive rule firings to have an order-independent result as explained by Ehrig et al. (2006). In our operator algebra language, these conditions would guarantee a zero commutator. The DPO approach was applied to molecular complexes in Danos and Laneve (2004). However, it requires the use of an abstract mathematical language (category theory) that poses a substantial barrier to understanding many biological modelers; direct use of the operator algebra developed by Heisenberg, Von Neumann, and others to formalize quantum mechanics in the 1920s is substantially more accessible, especially when, as in our case, it concerns probability distributions rather than quantum amplitudes.

Behr et al. (2016) and Behr et al. (2019) combined and connected both double-pushout and Master Equation semantics, using a restricted subset of the operator algebra implied by Propositions 1 or 2 in Mjolsness (2019a) or the more powerful Theorems 1 and 2 of this work. Commutators were introduced to this approach in Behr et al. (2016), but, apparently, without computing the explicit combinatorial result in Eq. 16, treating only the special case in which one of the operators, an “observable,” is diagonal in the number basis, a case which is potentially quite useful for pursuing moment closure approximation methods. They did not address the possibility in Proposition 1 of continuous parameters in the graph node labels or differential equation dynamics on those parameters. This approach has not been applied to the scientific domain of cell- or tissue-level morphodynamics in biological development.

Further discussion of the likely yet unproven relationship between our operator algebra semantics (Section 2.2) and the DPO semantics is provided in Mjolsness (2019a) (**Supplementary Section S7.2.10**).

Another work that associates a Lie algebra with a graph grammar is Marcoli and Port (2015). In this case, the basis Fock space over which the Lie algebra operators are defined is a space of labeled graphs *G*, rather than labeled graph grammar rules *G*^in^ → *G*^out^, so it is a different and smaller operator Lie algebra than ours. It seems closely related to a subalgebra of “graph insertions,” comprising rules whose left-hand side graph is a single node.

A hypergraph variant of graph grammars has recently been used as the starting part for a dark-horse attempt to find a fully discrete-mathematical route to fundamental physical theory (Wolfram, 2020). Many evocative examples are given and visualized as evolving graphs embedded in low-dimensional visualization space. Our operator algebra formulation, including Theorems 1 and 2, does not appear, nor is there an integration (e.g., Proposition 1) with continuous-time differential processes we require for efficient simulation of emergent, non-fundamental processes.

### 4.3 Domain of Applicability

The present line of research began as an approach to multicellular models of biological development that include cell birth, death, and geometry-induced changes in topology (Mjolsness et al., 1991; Jönsson et al., 2006; Mjolsness and Yosiphon, 2006). The graph grammar operator algebra was defined implicitly (by mapping to unique object IDs) in Mjolsness and Yosiphon (2006), a method used to implement general dynamical graph grammar models in Yosiphon (2009), and defined explicitly in Mjolsness (2010).

As discussed in Mjolsness (2019a), operator commutators provide an analytic tool when used with perturbation series expansions such as the Baker–Campbell–Hausdorff (BCH) theorem (as suggested for stochastic chemical reaction networks in Hellander et al. (2014) and rewrite operator algebras in Mjolsness and Yosiphon (2006) and Behr et al. (2019)) underlying operator splitting methods (Jahnke and Altıntan, 2010; MacNamara and Strang, 2016) or the Time-Ordered Product Expansion for Feynman diagrams underlying the Gillespie Stochastic Simulation Algorithm (SSA) and some of its generalizations, including integration with differential equations (Mjolsness, 2013), by which to derive both general and model-specific simulation algorithms and approximations and bound or estimate their errors from the perturbation series remainder terms. For example, operator splitting algorithms, including the exploitation of analytically solvable submodels (Jahnke and Altıntan, 2010) can be formulated and have their errors analyzed by way of commutation relations using BCH. If, for example, two rule firings are simulated out of order for algorithmic efficiency, their commutator (which could be zero) quantifies the error introduced. Operator commutators are also fundamental, of course, for understanding the causal structure of a dynamical model. For example, in the Wightman axioms for quantum field theory in the Minkowski metric, the “Locality” axiom specifies the commutation (or anti-commutation) of operators that act at points separated by spacelike displacements (Glimm and Jaffe, 1981).

Potential applications of dynamical graph grammars (including stochastic parameterized graph grammars) are legion, particularly in multiscale modeling. We claim they comprise a third major scientific computing paradigm on the same level of generality and applicability as 1) partial differential equations (PDEs) or 2) particle methods. These are the two most relevant parallel computing “design patterns” identified for high-performance computing (HPC) in the survey of Asanovic et al. (2009). The same source identifies graph algorithms as a design pattern ubiquitous across parallel computing fields, excluding HPC. This exclusion can now be removed. Dynamical graphs and their operators, optionally expressed by dynamical graph grammars, in principle, bring a third paradigm major into play for generic mathematical and algorithmic tools for computational science.

Examples and categories of examples that would be suitable for DGG description include the following:

- Cytoskeleton: application to plant cortical microtubule dynamics has been described already in Mjolsness (2019a) and will be a running example for Section 3.7.1 and Supplementary Material SB.1. An additional analogous example is the dynamic actin filament network in synapses during learning.
- The originally intended domain for DGGs was multicellular models of biological development that include cell birth, death, and geometry-induced changes in the topology of networks of cells whose adjacency relationships form a graph (Mjolsness et al., 1991), including topology-changing models of plant development in the *Arabidopsis* shoot apical meristems as in Jönsson et al. (2006). An explicit one-dimensional DGG (in textual OID form) for pattern formation and growth in the *Arabidopsis* root apical meristems is presented in Yosiphon (2009) and Mjolsness (2013). DGGs for dynamic developmental topologies such as abstract cell complexes and stratified spaces, *via* graph slice categories, are discussed in Mjolsness (2019a).
- Physical applications may include the dynamics of topological dislocations, defects, and fractures in materials, treated as sparse extended objects in communication with the dense extended object(s) comprising the material [e.g., in “dislocation dynamics” (Devincre et al., 2008; Vattré et al., 2014)].
- Axonal and dendritic arbor growth and retraction in microscope imagery of animal development, under the regulatory influence of key genes such as DSCAM (Santos et al., 2018), comprise a dynamic spatially embedded graph.
- Agent-based systems running on interaction graphs are widely used models in epidemiology (Venkatramanan et al., 2018), social science (Klein et al., 2018), and multiscale biological modeling (Letort et al., 2019). When agent-based systems take agent-agent connectivity to be not only a factor affecting the dynamics of particle-like state-bearing agents but also a time-varying component of the system state governed by its own dynamics, then the underlying mathematics may be well described by the local graph dynamics of DGG rule operators and DGGs are a candidate mathematical formalism (at a higher level of abstraction than computer code) for expressing such models.
- Approximate solution algorithms for partial differential equations frequently proceed by way of spatial discretization first, resulting in a grid or mesh of dynamic variables connected to neighbors that appear on the right-hand sides of a local ordinary differential equation (dynamical system) description. Time is then discretized inside the solution algorithm for the resulting differential equations. If the grid graph is adaptive by local rules, the approximation can be described by a dynamical graph grammar.
- Hypergraph models can also be represented *via* the standard mapping of (labeled) hypergraphs to (labeled) bipartite graphs that connect hypervertex-flavored vertices to hyperedge-flavored vertices and *vice versa*.
- There could be methodological connections to loop quantum gravity.

Most of these applications have in common some form of model reduction from a finer-scale description that does not need dynamical graph description. The standard model of fundamental physics encompasses intertwined particles and fields but not dynamical graphs. On the other hand, coarse-graining or upscaling often introduces dynamical connectivity descriptions suitable for dynamical graphs, so any modeling framework that is to be universal for or invariant under a broad class of model reductions needs something like the rule operators of DGGs.

Universality under model reduction is even better served if DGGs can also encompass partial differential equations (PDEs). As suggested above, DDGs, as described here, can express a wide variety of approximations to PDEs for spatial models, including approximations to continuum models described by PDEs. However, what is missing is the formalization entirely within DGGs of a graph limit that approaches continuous geometries, such as manifolds, cell complexes, and stratified spaces, as discussed in Mjolsness (2019a). Furthermore, a definition of graph limit based on the preservation of graph diffusion across scales was proposed by Scott and Mjolsness (2021). Graph diffusion has the advantage [over, e.g., graphons (Lovász, 2012)] that it is closely related to metric structure in the case of manifolds.

## Supplementary Material

Supplementary Material: Explicit Calculation of Structural Commutation Relations for Stochastic and Dynamical Graph Grammar Rule Operators in Biological Morphodynamics (the titular calculation, in full)

## Data Availability

The original contributions presented in the study are included in the article/Supplementary Material. Further inquiries can be directed to the corresponding author.
