# Supplementary material for: Explicit Calculation of Structural Commutation Relations for Stochastic and Dynamical Graph Grammar Rule Operators in Biological Morphodynamics
Source: Front Syst Biol. Author manuscript; Available in PMC 2023 Jan 26. (PMC9879069; doi:10.3389/fsysb.2022.898858)
Supplement: Supplementary Material: Explicit Calculation of Structural Commutation Relations for Stochastic and Dynamical Graph Grammar Rule Operators in Biological Morphodynamics (the titular calculation, in full) [file NIHMS1842397-supplement-Supplementary_Material__Explicit_Calculation_of_Structural_Commutation_Relations_for_Stochastic_and_Dynamical_Graph_Grammar_Rule_Operators_in_Biological_Morphodynamics__the_titular_calculation__in_full_.pdf]

Supplementary Material: Explicit Calculation of Structural Commutation Relations for Stochastic and Dynamical Graph Grammar Rule Operators in Biological Morphodynamics

Eric Mjolsness

Abstract

This Supplementary Material, Appendices A, B, and C, contains details of calculations reported and referred to in the published paper of nearly the same name appearing in Frontiers in Systems Biology (2022), https://www.frontiersin.org/articles/10.3389/fsysb.2022.898858/full.

[1] Departments of Computer Science and Mathematics, University of California Irvine CA 92697. Email: emj@uci.edu .

Appendix A Supplementary Material: Detailed calculation for graph grammars

Here we record detailed calculations that prove Theorem 1 and Theorem 2. Four corollaries of each of Theorems 1 and 2 are shown in Section 3 and are not repeated here.

SA.1 Diagonal context factors

Another useful form for Equation (5) is to factor out any graph K that is completely unchanged, having graph node labels $\lambda'_v = \lambda_v$ and graph edges that are all the same in labeled graphs G and G'. Then:

$$\hat{W}_r = \frac{1}{C_r(N_{\text{max free}})} \int d\mu_r(X)\, \rho_r(\boldsymbol{\lambda}(X), \boldsymbol{\lambda}'(X)) \sum_{\langle i_1,\ldots i_k\rangle_{\neq}} \hat{a}_{i_1,\ldots i_k}(G^{r\,\text{out}} \backslash K^r) N_{i_1,\ldots i_k}(K^r) a_{i_1,\ldots i_k}(G^{r\,\text{in}} \backslash K^r) \tag{53}$$

where $N_\alpha = \hat{a}_\alpha a_\alpha$ is an elementary diagonal 0/1-valued number operator and $N_{i_1,\ldots i_k}(K^r)$ is the product of such elementary diagonal operators for of the nodes and edges in graph K. Set $G \backslash K$ is the set difference of two graphs, i.e. the set (not necessarily a graph) of nodes and edges in G after removing those that are also in K. Because the $\hat{a}$, N, and a operators are nonoverlapping products of nonoverlapping elementary node/label and edge $\hat{a}$, N, and a operators, the corresponding diagonal operator D_r of Equation (1), required to conserve probability, is now easy to compute: globally, a becomes N, N remains N, and $\hat{a}$ becomes $Z = I - N$ as in Equation (19). Thus:

$$D_r = \frac{1}{C_r(N_{\text{max free}})} \int d\mu_r(X)\, \rho_r(\boldsymbol{\lambda}(X), \boldsymbol{\lambda}'(X)) \sum_{\langle i_1,\ldots i_k\rangle_{\neq}} Z_{i_1,\ldots i_k}(G^{r\,\text{out}} \backslash K^r) N_{i_1,\ldots i_k}(K^r) N_{i_1,\ldots i_k}(G^{r\,\text{in}} \backslash K^r) \tag{54}$$

In the special case of Equation (53) for which $G^{r\,\text{out}} = G^{r\,\text{in}}$ and the node labels $\lambda(X)$ are all constrained by ρ to be unchanged, then $K = G^{r\,\text{out}} = G^{r\,\text{in}}$ and the off-diagonal factors disappear, leaving only the diagonal operator. By substituting $Z = I - N$ and expanding, the D_r of Equation (54) appearing in the master equation to conserve probability for any rule r can be expanded out into an integer-weighted sum of such diagonal $\hat{W}_{r'}$ operator expressions.

SA.2 Normalization

The factor of $1/C_r(N_{\text{max free}})$ in Equation (5) accounts for a large number of equivalent states that could result from a rule firing, whose weight should add up to $\mathcal{O}(1)$. It reflects the fact that in operator algebra formalism reaction rates naturally follow the law of mass action, so that if (as one would hope) a large number $N_{\text{max free}}$ of unallocated node indices are available for creating new graph content then the net rate of creation for that content is proportionately very high; yet this factor should instead be unimportant, so we scale it out. Roughly, $C_r(N_{\text{max free}})$ should be $N_{\text{max free}}!/((N_{\text{max free}}) - m_r)!$ where m_r is the number of new nodes $|G^{r\,\text{out}}_{\text{nodes}} \backslash G^{r\,\text{in}}_{\text{nodes}}|$ appearing in the output graph but not the input graph. However, $N_{\text{max free}}$ should be much larger than m_r so that it does not change appreciably when graph nodes are created or destroyed, in which

case $C_r(N_{\text{max free}}) \simeq (N_{\text{max free}})^{m_r}$ with equality in the $N_{\text{max free}} \to +\infty$ limit. Then in the limit C_r is "multiplicative" for additive m_r (i.e. $(N_{\text{max free}})^{m_{r_1}}(N_{\text{max free}})^{m_{r_2}} = (N_{\text{max free}})^{m_{r_1}+m_{r_2}}$) as we assume for Theorems 1 and 2 below.

Another possible formula, $C_r(N) = (N)_{m_r} \equiv N!/(N-m_r)!$, is "multiplicative" :

$$C_{r_2;r_1}(N_{\text{max free}}) = C_{r_2}(N_{\text{max free}} - m_{r_1})C_{r_1}(N_{\text{max free}}) \tag{55}$$

but requires dynamic tracking of $N_{\text{max free}}$. Alternatively, C_r could be held constant by an index allocation mechanism such as that described in Section SA.4.2. (Thus, one could invent a memory gatekeeping mechanism similar to "malloc" in C, "new" in C++, and "cons" in Lisp, but expressed in operator algebraic notation for allocating one block of indices at a time, at the risk of some degree of unnecessary serialization.) A useful limit of this route is to set $N_{\text{max free}} = 1, C_r = 1$ (also multiplicative) by imposing a unique choice of new, unique index value for each new node generated in each rule firing; this method requires a suitable choice function. A hash function on the left hand side indices i_k and the rule number r would go much of the way towards defining such a choice function, but some occupancy state information may also be needed as input. Occupancy state information is discussed in Section SA.4.2 below.

SA.3 Operator algebra techniques

The expressions [...] in square brackets in Equation (11) need to be restored to normal order, with annihilators a_α to the right of (preceding) creation operators $\hat{a}_\alpha$.

SA.3.1 Elementary operators' algebra

To do this systematically we need various operator rules for $2x2$ elementary operators:

$$\hat{a} = \begin{pmatrix} 0 & 0 \\ 1 & 0 \end{pmatrix}, a = \begin{pmatrix} 0 & 1 \\ 0 & 0 \end{pmatrix} \text{ implies} \tag{56a}$$

$$\hat{a}a = N \equiv \begin{pmatrix} 0 & 0 \\ 0 & 1 \end{pmatrix}, \quad a\hat{a} = Z \equiv I - N = \begin{pmatrix} 1 & 0 \\ 0 & 0 \end{pmatrix}, \text{ and} \tag{56b}$$

$$[a_\alpha, \hat{a}_\beta] = \delta_{\alpha\beta}(I_\alpha - 2N_\alpha)I \qquad \text{Alternative for normal form calcs:} \tag{56c}$$

$$a_\alpha \hat{a}_\beta = \hat{a}_\beta a_\alpha - 2\delta_{\alpha\beta}\hat{a}_\alpha a_\alpha + \delta_{\alpha\beta} I_\alpha \tag{56d}$$

$$a_\alpha \hat{a}_\beta = (1-\delta_{\alpha\beta})\hat{a}_\beta a_\alpha + \delta_{\alpha\beta} Z_\alpha \tag{56e}$$

Then for calculational purposes we record these elementary relationships:

$$\begin{aligned} \hat{a}_\alpha^2 &= 0 = a_\alpha^2 \\ N_\alpha &\equiv \hat{a}_\alpha a_\alpha \quad \text{(diagonal)} \\ Z_\alpha &\equiv I_\alpha - N_\alpha \quad \text{(diagonal)} \\ a_\alpha \hat{a}_\beta &= (1-\delta_{\alpha\beta})\hat{a}_\beta a_\alpha + \delta_{\alpha\beta} Z_\alpha \end{aligned} \quad \text{and} \quad \begin{aligned} N_\alpha N_\alpha &= N_\alpha \\ Z_\alpha Z_\alpha &= Z_\alpha \end{aligned} \quad \text{and} \quad \begin{aligned} Z_\alpha a_\alpha &= a_\alpha \\ a_\alpha Z_\alpha &= 0 \\ Z_\alpha \hat{a}_\alpha &= 0 \\ \hat{a}_\alpha Z_\alpha &= \hat{a}_\alpha \end{aligned} \tag{57}$$

In addition, an extra multiplicative algebra sector governs the erasure operator $E \equiv Z + a$:

$$\begin{aligned} E_\alpha \equiv & \Pi_{0\,\alpha} \equiv Z_\alpha + a_\alpha \\ E_\alpha a_\alpha &= a_\alpha \\ a_\alpha E_\alpha &= 0 \\ E_\alpha \hat{a}_\alpha &= Z_\alpha \\ \hat{a}_\alpha E_\alpha &= \Pi_{1\,\alpha} \end{aligned} \quad \text{and} \quad \begin{aligned} \Pi_{1\,\alpha} \equiv & \hat{a}_\alpha + N_\alpha \\ \Pi_{1\,\alpha} a_\alpha &= N_\alpha \\ a_\alpha \Pi_{1\,\alpha} &= \Pi_{0\alpha} \\ \Pi_{1\,\alpha} \hat{a}_\alpha &= \hat{a}_\alpha \\ \hat{a}_\alpha \Pi_{1\,\alpha} &= 0 \end{aligned} \tag{58}$$

In order to control the signs of integer-valued weights in operator products, we observe the following: For creation/annihilation operators pertaining to graph edges, including those making up the edge erasure operators $E_{i_p\,i}$ and $E_{i\,i_q}$, using e.g. Equation (56e) rather than (56d) removes the explicit negative signs from the algebra by introducing matrix $Z_{i_p\,i_q}$ which has nonnegative entries.

This algebra governs the graph edge creation and annihilation operators, for which $\alpha = (i, j)$. It does not apply directly to the node label creation and annihilation operators, except as targets of an operator homomorphism to be described next. For this homomorphism the elementary bitwise operators obeying the algebra above will be denoted "b" rather than "a".

SA.4 Operator Algebra homomorphisms

A homomorphism of operator algebras is defined here as a mapping from one operator algebra to another that preserves the basic algebraic operations: finite sums, scalar multiplication, and finite products of operators. It is thus a ring homomorphism, for a ring of linear operators that act on a vector space. In our case the vector space is a Fock space capable of hosting classical probability distributions [1, 2, 3] If the operator algebra homomorphism is also injective, it could be called an "embedding".

SA.4.1 Winner Take All (WTA or 1-Hot) Encoding of Labels

We can enforce a winner-might-take-all logic of labels either by fiat using axioms:

$$\begin{aligned} a_{i,\lambda} a_{i,\lambda'} =& 0 \\ \hat{a}_{i,\lambda} \hat{a}_{i,\lambda'} =& 0 \\ a_{i,\lambda} \hat{a}_{i,\lambda'} =& \delta_{\lambda\,\lambda'} Y_{i,\lambda'}. \end{aligned} \tag{59}$$

where $N^{(a)}_{i,\lambda'}$ and $Y_{i,\lambda'}$ are diagonal in the number basis and idempotent, satisfying

$$\begin{aligned} Y_\alpha a_\alpha =& a_\alpha \\ a_\alpha Y_\alpha =& 0 \\ Y_\alpha \hat{a}_\alpha =& 0 \\ \hat{a}_\alpha Y_\alpha =& \hat{a}_\alpha \end{aligned} \quad \text{and} \quad \begin{aligned} Y_\alpha a_\beta =& a_\alpha Y_\beta \quad \text{for } (\alpha \neq \beta) \\ Y_\alpha \hat{a}_\beta =& \hat{a}_\beta Y_\alpha \quad \text{for } (\alpha \neq \beta) \\ Y_\alpha Y_\alpha =& Y_\alpha \end{aligned} \tag{60}$$

for $\alpha = (i, \lambda)$ as appropriate for node labels, just as Z does in Equation (57). Likewise for N:

$$\begin{aligned} N_\alpha a_\alpha =& 0 \\ a_\alpha N_\alpha =& a_\alpha \\ N_\alpha \hat{a}_\alpha =& \hat{a}_\alpha \\ \hat{a}_\alpha N_\alpha =& 0 \end{aligned} \quad \text{and} \quad \begin{aligned} N_\alpha a_\beta =& a_\alpha N_\beta \quad \text{for } (\alpha \neq \beta) \\ N_\alpha \hat{a}_\beta =& \hat{a}_\beta N_\alpha \quad \text{for } (\alpha \neq \beta) \\ N_\alpha N_\alpha =& N_\alpha \end{aligned} \tag{61}$$

and $N_\alpha Y_\alpha = 0 = Y_\alpha N_\alpha$; also $N_\alpha Y_\beta = Y_\beta N_\alpha$.

Alternatively, we can ground this WTA algebra in terms of the usual elementary 0/1-valued states using the 0/1-winner mapping

$$\begin{aligned} a_{i,\lambda} &= \hat{b}_{i,\varnothing} b_{i,\lambda} \\ \hat{a}_{i,\lambda} &= \hat{b}_{i,\lambda} b_{i,\varnothing} \end{aligned} \tag{62}$$

in which the $b, \hat{b}$ operators obey the bitwise algebra of Equation (56) above, and they *also* by induction obey the WTA/one-hot subspace constraint imposed by initial condition and preserved by operators constructed from $a, \hat{a}$:

$$\begin{aligned} N_{i,\varnothing} + \sum_\lambda N^{(b)}_{i,\lambda} &\simeq I \ , \\ b_{i,\varnothing} b_{i,\lambda} &\simeq 0 \simeq b_{i,\lambda} b_{i,\varnothing} \ , \\ b_{i,\lambda} b_{i,\lambda'} &\simeq 0 \quad . \end{aligned} \tag{63}$$

In the number basis for b, these equivalences follow from the initialization and inductive preservation of

$$\begin{aligned} n_{i,\varnothing} + \sum_\lambda n^{(b)}_{i,\lambda} &= 1 \\ n_{i,\varnothing},\, n^{(b)}_{i,\lambda} &\in \{0,1\} \end{aligned} \tag{64}$$

so that $n_{i,\varnothing} n^{(b)}_{i,\lambda} = 0$ and $\lambda \neq \lambda' \implies n^{(b)}_{i,\lambda} n^{(b)}_{i,\lambda'} = 0$; then use $b_\alpha | \ldots n_\alpha \ldots \rangle = n^{(b)}_\alpha | \ldots (n_\alpha - 1) \ldots \rangle$.

Using this algebra for $b, \hat{b}$ and the operator algebra homomorphism to $a, \hat{a}$ induced by Equation (62), then the $a, \hat{a}$ algebra of Equations (59),(60), and (61) (interpreting N in (59)-(61) as $N^{(a)}$ below, not as $N^{(b)}$) can be verified by direct computation. We find the additional homomorphism mappings to the bitwise "b" algebra for Y and $N^{(a)}$:

$$\begin{aligned} N^{(a)}_{i,\lambda} &= N^{(b)}_{i,\lambda} Z_{i,\varnothing} = \hat{b}_{i,\lambda} b_{i,\lambda} b_{i,\varnothing} \hat{b}_{i,\varnothing} \\ Y_{i,\lambda} &= Z^{(b)}_{i,\lambda} N_{i,\varnothing} = b_{i,\lambda} \hat{b}_{i,\lambda} \hat{b}_{i,\varnothing} b_{i,\varnothing} \end{aligned} \tag{65}$$

Of course operators indexed by nodes $i \neq j$ all commute. Combined with the last line of Equation (59), this fact produces a major calculational tool for nodes in the form of the following key commutation relation simply repeating Equation (22) :

$$\boxed{a_{j,\lambda} \hat{a}_{i,\lambda'} = (1 - \delta_{ij}) \hat{a}_{i,\lambda'} a_{j,\lambda} + \delta_{ij} \delta_{\lambda\lambda'} Y_{j,\lambda'}} \tag{66}$$

This relation differs from Equation (56e) in producing fewer nonzero results, so it is more constraining, and a slightly different diagonal operator Y obeying the same algebra for node labels (in Equation (60)) as Z (in Equation (57)) does for edges. Equation (56e) however still governs edge operators.

Thus we reach sufficient multiplicative information on $\{a, \hat{a}, N, Z, Y, E, \Pi_1\}$ in principle to compute all products of $\hat{W}$ operators.

SA.4.2 Controlled index allocation

Each graph rewrite rule may introduce new graph nodes not already present. The graph rewrite rule algebra will be simpler if these can be modeled with fresh node indices i not previously used - even if some further algebra homomorphism and remapping not undertaken here actually reuses old, deallocated graph node indices. (Edge index pairs will necessarily be fresh - heretofore unused - if at least one of their node indices is fresh.) Here we just seek to express algebraically a continual, parallelism-compatible supply of fresh indices. Choose an index block size B that is large enough to encompass the new nodes of any rule we consider. The chosen B could even be countably infinite, e.g. if we use a diagonal raster traversal of B and the infinite collection of blocks needed; however in Theorem 2 we will assume B is finite. Index the blocks needed by $\mu \in M$ where M is a countably infinite tree of finite maximum branching degree, and let $\phi \subseteq M$ denote a frontier in M: the collection of next blocks available for allocation, whose ancestors have all been allocated.

As a special case, if M is a graph isomorphic to the integers with succession ($\mathbb{N}^+$) as the tree relationship, then this scheme will force serial computation; but an average branching degree even slightly greater than 1 permits parallelism.

Each block τ has binary variables $A_\tau \in \{0,1\}$ taking the value 1 if and only if block τ is "allocated" or "alive" (in which case all of τ's ancestors mush also be alive), and $F_\tau \in \{0,1\}$ taking the value 1 if and only if block τ is in the current frontier ϕ, in which case $A_\tau = 1$ but all of τ's children must be unallocated ($A_{\sigma \in \text{children}(\tau)} = 0$). These binary variables F have creation/annihilation operators $\hat{b}^{\text{ind}}_\tau$ and b^{ind}_τ. Then $|\phi| = \sum_{\tau \in M} F_\tau$.

We will assume that nodes i which have never been allocated in a memory block all obey the initial condition that $n_{i,\varnothing} = 1$ and $n_{i,\lambda} = 0$ for other labels λ and inductively have no way of changing until the memory block τ containing i is allocated; and likewise all the edge numbers n_{ij} and n_{ji} involving node i are all initialized to zero and inductively have no way of changing until the memory blocks σ, τ containing i and j respectively are both allocated.

Let $\text{Ch}(\tau)$ be the set of child blocks of memory block τ in M. Then the combined operator

$$\text{Advance}_\tau \equiv \Big[\prod_{\sigma \in \text{Ch}(\tau)} \hat{b}^{\text{ind}}_\sigma \Big] b^{\text{ind}}_\tau \tag{67}$$

could be used to advance the frontier ϕ of allocated memory under a single rule firing. (M could even be permitted to be a directed acyclic graph, if the child operator $\hat{b}^{\text{ind}}_\sigma$ in the product in Equation (67) is replaced by $(\hat{b}^{\text{ind}}_\sigma + N^{\text{ind}}_\sigma)$. Then child memory blocks that are already alive and in the frontier are permitted, and remain that way.) If we initialize the aliveness and frontier at the root of the tree and maintain it by Equation (67) inductively, then we can take

$$a^{\text{ind}}_\tau = b^{\text{ind}}_\tau \quad \text{and} \quad \hat{a}^{\text{ind}}_\tau = \hat{b}^{\text{ind}}_\tau \tag{68}$$

More conservatively we could continually check that old memory is not about to be reused incorrectly:

$$a^{\text{ind}}_\tau = b^{\text{ind}}_\tau \quad \text{and} \quad \hat{a}^{\text{ind}}_\tau = \hat{b}^{\text{ind}}_\tau \Big(\prod_{\sigma \in \text{ancestors}(\tau)} Z^{\text{ind}}_\sigma \Big) \tag{69}$$

The index allocation frontier maps to parallel computational architectures in which time can be local, for example time can be a spacelike foliation of spacetime that respects signal propagation delays.

Now the idea is that rule-firing operators $\hat{W}_r$ will act also in the index allocation space, using and then advancing the frontier ϕ of blocks τ from which newly allocated graph nodes i can be drawn. Denoting by $\hat{W}_{r,\tau}$ the variant of $\hat{W}_r$ that draws all newly allocated nodes i from block τ (the block size B being always large enough for this), then

$$\hat{W}_r \equiv \sum_{\tau \in M} \Big[\prod_{\sigma \in \mathrm{Ch}(\tau)} \hat{a}^{\mathrm{ind}}_{\sigma} \Big] \hat{W}_{r\tau} a^{\mathrm{ind}}_{\tau} \frac{1}{|\phi|}. \tag{70}$$

where all such expressions as τ varies are regarded as equivalent owing to index permutation invariance and operator linearity. In the special case $M = \mathbb{N}^+$, $\phi = \{\tau\}, |\phi| = 1$ and this operator becomes

$$\hat{W}_r \equiv \sum_{\tau \in M} \hat{a}^{\mathrm{ind}}_{\tau+1} \hat{W}_{r\tau} a^{\mathrm{ind}}_{\tau} \tag{71}$$

(cf. [4], a quantum version that adds in the time-reversal Hermitian conjugate of all transitions) which is the form we will assume. Parallel computational implementations with a large frontier should be equivalent, in the sense of Equation (8), to this simplest case.

With more complex dynamics one could try to ensure that in Equation (70) the $|\phi|$, size of the frontier, is constant or nearly constant in time, and move its inverse to the left of the $\sum_\tau$ above. For example M could be a root node connected to the zero nodes of $|\phi|$ half-infinite chains each isomorphic to the integers under succession. Alternatively one could track the relationship between simulated and computational time. In what follows we'll assume one of these options has been taken, so that the factor of $1/|\phi|$ is the same for all rules, treat the general M case as *equivalent* (using $\simeq$ as previously defined) to the special case $M = \mathbb{N}^+$, $\phi = \{\tau\}, |\phi| = 1$ that we assume in the calculations that follow.

Similar "aliveness" variables in quantitative grammar models have been used in [5] and [6], along with winner-take-all variable subset constraints, though without the operator algebra framework. Controlled index allocation could be related in a computational implementation to controlled memory allocation.

SA.4.3 Hanging edge cleanup

Another elaboration of rule operators $\hat{W}_r$ can clean up hanging edges that may otherwise be left behind by a rule firing:

$$\hat{W}^{\mathrm{cleaned}}_r = \Big(\prod_{k_1 \in L_r \backslash R_r} \prod_{k_2 \in \mathcal{U}} E_{k_1 k_2} E_{k_2 k_1} \Big) \hat{W}^{\mathrm{bare}}_r \simeq \Big(\prod_{(k_1,k_2) \in \mathcal{S}} E_{k_1 k_2} \Big) \Big(\prod_{(k_1,k_2) \in \mathcal{S}} E_{k_2 k_1} \Big) \hat{W}^{\mathrm{bare}}_r \tag{72}$$

where $\mathcal{S}$ is the set of indices specified by

$$\mathcal{S} = [(L_r \backslash R_r) \times \mathcal{U}_{A*}] \tag{73}$$

where $\mathcal{U}_{A*}$ = all node indices that have ever been allocated in a memory block, hence all memory-live node indices, and $\mathcal{U}$ = the whole universe of node indices, so that $\mathcal{U}_{A*} \subseteq \mathcal{U}$. The second line in Equation (72) is *equivalent*, $\simeq$ to the first because as discussed above, unallocated k_2 indices inductively have $n_{k_1 k_2} = 0 = n_{k_2 k_1}$, and the erasure operator does nothing (is equivalent to the

identity operator) in that case. The reason for including this restriction in the definition of $\mathcal{S}$ is that, for rules with finite graphs and index allocation with finite block size and after any finite number of rule firings, $\mathcal{U}_{A*}$ is finite and both factors of the set $\mathcal{S}$ are finite, so $\mathcal{S}$ itself is finite; only a finite amount of cleanup work needs to be done for each rule firing. We will use this assumption in the proof of Theorem 2.

In the next section we will use the notation $\mathcal{P}_\chi = [(L_\chi \backslash R_\chi) \times \mathcal{U}]$ for the predicate that designates the possibly infinite superset of index set $\mathcal{S}$ above, that pertains to the top line of Equation (72).

In greater detail the hanging-edge removal semantics as specified less formally in the top line of Equation (72) above, is given by Equations (23) and (24) of Section 3.2.

Also, index notation will be used as specified by Equations (23)- (29) of Section 3.3. (In connection with Equation (29), we add here that any of these alternative formulations of $\mathcal{P}$ would be equivalent:

$$\begin{array}{rl} \mathrm{I}: \mathcal{P}_\chi = [(L_\chi \backslash R_\chi) \times \mathcal{U}] & \mathcal{P}^*_\chi = \mathcal{P}^T_\chi = [\mathcal{U} \times (L_\chi \backslash R_\chi)] \\ \mathrm{II}: \mathcal{P}_\chi = [(L_\chi \backslash R_\chi) \times \overline{L_\chi \cup R_\chi}] & \underset{\longleftrightarrow}{\text{duals}} \; \mathcal{P}^*_\chi = \mathcal{P}^T_\chi = [\overline{L_\chi \cup R_\chi} \times (L_\chi \backslash R_\chi)] \\ \mathrm{III}: \mathcal{P}_\chi = [(L_\chi \backslash R_\chi) \times \mathcal{U}] \backslash (\mathcal{L}_\chi \backslash \mathcal{R}_\chi)] & \mathcal{P}^*_\chi = [\mathcal{U} \times (L_\chi \backslash R_\chi)] \backslash (\mathcal{L}_\chi \backslash \mathcal{R}_\chi)] \\ \mathrm{IV}: \mathcal{P}_\chi = [(L_\chi \backslash R_\chi) \times \overline{L_\chi \cup R_\chi}] \backslash (\mathcal{L}_\chi \backslash \mathcal{R}_\chi)]; & \mathcal{P}^*_\chi = [\overline{L_\chi \cup R_\chi} \times (L_\chi \backslash R_\chi)] \backslash (\mathcal{L}_\chi \backslash \mathcal{R}_\chi)]. \end{array} \quad (74)$$

However, we will use Equation (29) = case I above, since it is the easiest to work with.)

Denote the sought-after "compound rule" for rules r_1 followed by r_2 as $r_{1;2}$. Calculate $L_{1;2} = L_1 \cup (L_2 \backslash R_1) = L_1 \cup (L_2 \backslash \mathcal{I}(S))$ because $\mathcal{I}(S) \subseteq R_1$ and $L_2 \cap R_1 \subseteq \mathcal{I}(S)$; likewise $R_{1;2} = R_2 \cup (R_1 \backslash L_2) = R_2 \cup (R_1 \backslash \mathcal{I}(S))$ because $\mathcal{I}(S) \subseteq L_2$ and $R_1 \cap L_2 \subseteq \mathcal{I}(S)$. Similarly for $\mathcal{L}_{1;2}$ and $\mathcal{R}_{1;2}$.

Then we have these compound rule index set definitions:

$$\boxed{\begin{array}{rll} L_{1;2} = & L_1 \cup (L_2 \backslash R_1) & = L_1 \cup (L_2 \backslash \mathcal{I}(S)) \\ R_{1;2} = & R_2 \cup (R_1 \backslash L_2) & = R_2 \cup (R_1 \backslash \mathcal{I}(S)) \\ \Delta = & (L_2 \backslash R_2) \cap (R_1 \backslash L_1) \equiv \overline{R_2} \cap L_2 \cap R_1 \cap \overline{L_1} & \\ \mathcal{L}_{1;2} = & \mathcal{L}_1 \cup (\mathcal{L}_2 \backslash \mathcal{R}_1) & = \mathcal{L}_1 \cup (\mathcal{L}_2 \backslash \mathcal{I}(H_{1\ \mathrm{links}})) \\ \mathcal{R}_{1;2} = & \mathcal{R}_2 \cup (\mathcal{R}_1 \backslash \mathcal{L}_2) & = \mathcal{R}_2 \cup (\mathcal{R}_1 \backslash \mathcal{I}(H_{1\ \mathrm{links}})) \\ \mathcal{D} = & (\mathcal{L}_2 \backslash \mathcal{R}_2) \cap (\mathcal{R}_1 \backslash \mathcal{L}_1) \equiv \overline{\mathcal{R}_2} \cap \mathcal{L}_2 \cap \mathcal{R}_1 \cap \overline{\mathcal{L}_1} & \end{array}}\ . \quad (75)$$

The index sets Δ and $\mathcal{D}$ above will turn up in the calculation of the next section.

From $a^2 = 0 = \hat{a}^2$, we have:

$$\begin{aligned} (R_1 \backslash L_1) \cap (R_2 \backslash L_2) &= \varnothing \\ (L_1 \backslash R_1) \cap (L_2 \backslash R_2) &= \varnothing \\ (\mathcal{R}_1 \backslash \mathcal{L}_1) \cap (\mathcal{R}_2 \backslash \mathcal{L}_2) &= \varnothing \\ (\mathcal{L}_1 \backslash \mathcal{R}_1) \cap (\mathcal{L}_2 \backslash \mathcal{R}_2) &= \varnothing. \end{aligned} \quad (76)$$

SA.5 Commutation calculation - no edge cleanup

The product of two such operators is (omitting for now the integral over parameters X)

$$\begin{aligned}\hat{W}_{r_2}\hat{W}_{r_1} &= \frac{1}{C_{r_1}(N_{\text{max free}})}\frac{1}{C_{r_2}(N_{\text{max free}})}\rho_{r_1}(\boldsymbol{\lambda}^{(1)},\boldsymbol{\lambda}'^{(1)})\rho_{r_2}(\boldsymbol{\lambda}^{(2)},\boldsymbol{\lambda}'^{(2)}) \sum_{\mathcal{J}:L_2\cup R_2 \overset{1\text{-}1}{\hookrightarrow}\mathcal{U}} \sum_{\mathcal{I}:L_1\cup R_1 \overset{1\text{-}1}{\hookrightarrow}\mathcal{U}} \\ &\times \left[\prod_{(j_1,j_2)\in\mathcal{R}_2}\hat{a}_{j_1j_2}\right]\left[\prod_{(j_3,j_4)\in\mathcal{L}_2}a_{j_3j_4}\right]\left[\prod_{j_5\in R_2}\hat{a}_{j_5,\lambda'^{(2)}_{\mathcal{J}^{-1}(j_5)}}\right]\left[\prod_{j_6\in L_2}a_{j_6,\lambda^{(2)}_{\mathcal{J}^{-1}(j_6)}}\right] \\ &\times \left[\prod_{(i_1,i_2)\in\mathcal{R}_1}\hat{a}_{i_1i_2}\right]\left[\prod_{(i_3,i_4)\in\mathcal{L}_1}a_{i_3i_4}\right]\left[\prod_{i_5\in R_1}\hat{a}_{i_5,\lambda'^{(1)}_{\mathcal{I}^{-1}(i_5)}}\right]\left[\prod_{i_6\in L_1}a_{i_6,\lambda^{(1)}_{\mathcal{I}^{-1}(i_6)}}\right]\end{aligned} \tag{77}$$

Grouping the node operators together at the end, and grouping together terms that need to be commuted next as $\{\dots\}_{①}$ and $\{\dots\}_{②}$, this is:

$$\begin{aligned}\hat{W}_{r_2}\hat{W}_{r_1} =& \frac{1}{C_{r_1}(N_{\text{max free}})}\frac{1}{C_{r_2}(N_{\text{max free}})}\rho_{r_1}(\boldsymbol{\lambda}^{(1)},\boldsymbol{\lambda}'^{(1)})\rho_{r_2}(\boldsymbol{\lambda}^{(2)},\boldsymbol{\lambda}'^{(2)}) \\ &\times \sum_{\mathcal{J}:L_2\cup R_2 \overset{1\text{-}1}{\hookrightarrow}\mathcal{U}} \sum_{\mathcal{I}:L_1\cup R_1 \overset{1\text{-}1}{\hookrightarrow}\mathcal{U}} \left[\prod_{(j_1,j_2)\in\mathcal{R}_2}\hat{a}_{j_1j_2}\right] \\ &\times \left\{\left[\prod_{(j_3,j_4)\in\mathcal{L}_2}a_{j_3j_4}\right]\left[\prod_{(i_1,i_2)\in\mathcal{R}_1}\hat{a}_{i_1i_2}\right]\right\}_{①}\left[\prod_{(i_3,i_4)\in\mathcal{L}_1}a_{i_3i_4}\right]\left[\prod_{j_5\in R_2}\hat{a}_{j_5,\lambda'^{(2)}_{\mathcal{J}^{-1}(j_5)}}\right] \\ &\times \left\{\left[\prod_{j_6\in L_2}a_{j_6,\lambda^{(2)}_{\mathcal{J}^{-1}(j_6)}}\right]\left[\prod_{i_5\in R_1}\hat{a}_{i_5,\lambda'^{(1)}_{\mathcal{I}^{-1}(i_5)}}\right]\right\}_{②}\left[\prod_{i_6\in L_1}a_{i_6,\lambda^{(1)}_{\mathcal{I}^{-1}(i_6)}}\right]\end{aligned} \tag{78}$$

Strategically rewriting the sum over $\mathcal{J}$,

$$\boxed{\begin{aligned}\hat{W}_{r_2}\hat{W}_{r_1} =& \frac{1}{C_{r_1}(N_{\text{max free}})}\frac{1}{C_{r_2}(N_{\text{max free}})}\rho_{r_1}(\boldsymbol{\lambda}^{(1)},\boldsymbol{\lambda}'^{(1)})\rho_{r_2}(\boldsymbol{\lambda}^{(2)},\boldsymbol{\lambda}'^{(2)}) \\ &\times \sum_{T\subseteq G^{r_1\,\text{in}}_{\text{nodes}}\setminus G^{r_1\,\text{out}}_{\text{nodes}}} \sum_{\pi:T\overset{1\text{-}1}{\hookrightarrow}G^{r_2\,\text{out}}_{\text{nodes}}\setminus G^{r_2\,\text{in}}_{\text{nodes}}} \sum_{S\subseteq G^{r_1\,\text{out}}_{\text{nodes}}} \sum_{h:S\overset{1\text{-}1}{\hookrightarrow}G^{r_2\,\text{in}}_{\text{nodes}}} \\ & \sum_{\mathcal{I}:L_1\cup R_1 \overset{1\text{-}1}{\hookrightarrow}\mathcal{U}} \sum_{\substack{\mathcal{J}:L_2\cup R_2 \overset{1\text{-}1}{\hookrightarrow}\mathcal{U} \\ \text{Im}(\mathcal{I})\cap\text{Im}(\mathcal{J})=\mathcal{I}(S)\cup\mathcal{I}(T) \\ \mathcal{I}(S)=\mathcal{J}(h(S))\wedge\mathcal{I}(T)=\mathcal{J}(\pi(T))}} \left[\prod_{(j_1,j_2)\in\mathcal{R}_2}\hat{a}_{j_1j_2}\right] \\ &\times \left\{\left[\prod_{(j_3,j_4)\in\mathcal{L}_2}a_{j_3j_4}\right]\left[\prod_{(i_1,i_2)\in\mathcal{R}_1}\hat{a}_{i_1i_2}\right]\right\}_{①}\left[\prod_{(i_3,i_4)\in\mathcal{L}_1}a_{i_3i_4}\right]\left[\prod_{j_5\in R_2}\hat{a}_{j_5,\lambda'^{(2)}_{\mathcal{J}^{-1}(j_5)}}\right] \\ &\times \left\{\left[\prod_{j_6\in L_2}a_{j_6,\lambda^{(2)}_{\mathcal{J}^{-1}(j_6)}}\right]\left[\prod_{i_5\in R_1}\hat{a}_{i_5,\lambda'^{(1)}_{\mathcal{I}^{-1}(i_5)}}\right]\right\}_{②}\left[\prod_{i_6\in L_1}a_{i_6,\lambda^{(1)}_{\mathcal{I}^{-1}(i_6)}}\right]\end{aligned}} \tag{79}$$

In the controlled index model the sum over maps $\mathcal{J}$ will simplify because T and $\pi(T)$ will both be the null set. In any case, note that

$$\mathcal{I}(T) \subseteq L_1\setminus R_1 \quad \text{and} \quad \mathcal{J}(\pi(T)) \subseteq R_2\setminus L_2 \quad . \tag{80}$$

The more constraining form to commute is $\{\dots\}_{②}$:

$$\left\{\left[\prod_{j_6\in L_2} a_{j_6,\lambda^{(2)}_{\mathcal{J}^{-1}(j_6)}}\right]\left[\prod_{i_5\in R_1}\hat{a}_{i_5,\lambda'^{(1)}_{\mathcal{I}^{-1}(i_5)}}\right]\right\}_{②}=\left[\prod_{j_{6\,0}\in L_2\cap\mathcal{I}(S)}\ \prod_{j_{6\,1}\in L_2\cap\overline{\mathcal{I}(S)}} a_{j_{6\,0},\lambda^{(2)}_{\mathcal{J}^{-1}(j_{6\,0})}}a_{j_{6\,1},\lambda^{(2)}_{\mathcal{J}^{-1}(j_{6\,1})}}\right]$$
$$\times\left[\prod_{i_{5\,0}\in R_1\cap\mathcal{I}(S)}\ \prod_{i_{5\,1}\in R_1\cap\overline{\mathcal{I}(S)}}\hat{a}_{i_{5\,0},\lambda'^{(1)}_{\mathcal{I}^{-1}(i_{5\,0})}}\hat{a}_{i_{5\,1},\lambda'^{(1)}_{\mathcal{I}^{-1}(i_{5\,1})}}\right]. \tag{81}$$

Using Equation (66) $a_{j,\lambda}\hat{a}_{i,\lambda'}=(1-\delta_{ij})\hat{a}_{i,\lambda'}a_{j,\lambda}+\delta_{ij}\delta_{\lambda\lambda'}Y_{j,\lambda'}$, so that only indices in $\mathcal{I}(S)$ may fail to commute, this becomes $\{\dots\}_{②}$:

$$\left\{\left[\prod_{j_6\in L_2} a_{j_6,\lambda^{(2)}_{\mathcal{J}^{-1}(j_6)}}\right]\left[\prod_{i_5\in R_1}\hat{a}_{i_5,\lambda'^{(1)}_{\mathcal{I}^{-1}(i_5)}}\right]\right\}_{②}$$
$$=\prod_{j_{6\,0}\in L_2\cap\mathcal{I}(S)=\mathcal{I}(S)=L_2\cap R_1}\ \prod_{j_{6\,1}\in L_2\cap\overline{\mathcal{I}(S)}=L_2\cap\overline{R_1}}\ \prod_{i_{5\,0}\in R_1\cap\mathcal{I}(S)}\ \prod_{i_{5\,1}\in R_1\cap\overline{\mathcal{I}(S)}}$$
$$a_{j_{6\,0},\lambda^{(2)}_{\mathcal{J}^{-1}(j_{6\,0})}}a_{j_{6\,1},\lambda^{(2)}_{\mathcal{J}^{-1}(j_{6\,1})}}\hat{a}_{i_{5\,0},\lambda'^{(1)}_{\mathcal{I}^{-1}(i_{5\,0})}}\hat{a}_{i_{5\,1},\lambda'^{(1)}_{\mathcal{I}^{-1}(i_{5\,1})}}$$
$$=\prod_{j_{6\,0}\in L_2\cap R_1}\ \prod_{j_{6\,1}\in L_2\cap\overline{R_1}}\ \prod_{i_{5\,0}\in L_2\cap R_1}\ \prod_{i_{5\,1}\in R_1\cap\overline{L_2}}\hat{a}_{i_{5\,1},\lambda'^{(1)}_{\mathcal{I}^{-1}(i_{5\,1})}}a_{j_{6\,1},\lambda^{(2)}_{\mathcal{J}^{-1}(j_{6\,1})}}a_{j_{6\,0},\lambda^{(2)}_{\mathcal{J}^{-1}(j_{6\,0})}}\hat{a}_{i_{5\,0},\lambda'^{(1)}_{\mathcal{I}^{-1}(i_{5\,0})}}$$
$$=\prod_{j_{6\,1}\in L_2\cap\overline{R_1}}\ \prod_{i_{5\,0}\in L_2\cap R_1}\ \prod_{i_{5\,1}\in R_1\cap\overline{L_2}}\hat{a}_{i_{5\,1},\lambda'^{(1)}_{\mathcal{I}^{-1}(i_{5\,1})}}a_{j_{6\,1},\lambda^{(2)}_{\mathcal{J}^{-1}(j_{6\,1})}}\delta_{\lambda'^{(1)}_{\mathcal{I}^{-1}(i_{5\,0})},\lambda^{(2)}_{\mathcal{J}^{-1}(j_{6\,1})=h(\mathcal{I}^{-1}(i_{5\,0}))}}Y_{i_{5\,0},\lambda'^{(1)}_{\mathcal{I}^{-1}(i_{5\,0})}} \tag{82}$$

and thus

$$\left\{\left[\prod_{j_6\in L_2} a_{j_6,\lambda^{(2)}_{\mathcal{J}^{-1}(j_6)}}\right]\left[\prod_{i_5\in R_1}\hat{a}_{i_5,\lambda'^{(1)}_{\mathcal{I}^{-1}(i_5)}}\right]\right\}_{②}=\left[\prod_{i_{5\,1}\in R_1\setminus L_2}\hat{a}_{i_{5\,1},\lambda'^{(1)}_{\mathcal{I}^{-1}(i_{5\,1})}}\right]\left[\prod_{j_{6\,1}\in L_2\setminus R_1}a_{j_{6\,1},\lambda^{(2)}_{\mathcal{J}^{-1}(j_{6\,1})}}\right]$$
$$\times\left[\prod_{i_{5\,0}\in L_2\cap R_1}\delta_{\lambda'^{(1)}_{\mathcal{I}^{-1}(i_{5\,0})},\lambda^{(2)}_{h(\mathcal{I}^{-1}(i_{5\,0}))}}Y_{i_{5\,0},\lambda'^{(1)}_{\mathcal{I}^{-1}(i_{5\,0})}}\right] \tag{83}$$

since on S, $\mathcal{J}^{-1}=h\circ\mathcal{I}^{-1}$. The last line implements label-checking in the node correspondence portion of graph matching between a subgraph $H(S,h)$ of the output graph of rule r_1 and a corresponding subgraph of the input graph of rule r_2.

We must now simplify $\{\dots\}_{①}$ by commuting its leftmost factor, $\left[\prod_{(j_3,j_4)\in\mathcal{R}_2}a_{j_3j_4}\right]$, to the right of its rightmost factor, $\left[\prod_{(i_1,i_2)\in\mathcal{R}_1}\hat{a}_{i_1i_2}\right]$. To this end, using Equation (56) and the conditions $\mathrm{Im}(\mathcal{I})\cap\mathrm{Im}(\mathcal{J})=\mathcal{I}(S)\cup\mathcal{I}(T)$, and (from Equation (80)) $\mathcal{I}(T)\cap R_1=\varnothing\wedge\mathcal{J}(\pi(T))\cap L_2=\varnothing$, we compute:

$$\left\{\left[\prod_{(j_3,j_4)\in\mathcal{L}_2} a_{j_3j_4}\right]\left[\prod_{(i_1,i_2)\in\mathcal{R}_1} \hat{a}_{i_1i_2}\right]\right\}_{①} = \left[\prod_{(j_3,j_4)\in\mathcal{L}_2}\ \prod_{(i_1,i_2)\in\mathcal{R}_1} a_{j_3j_4}\hat{a}_{i_1i_2}\right]$$
$$= \left[\prod_{(i_1,i_2)\in\mathcal{R}_1\backslash\mathcal{L}_2\subseteq\overline{[L_2\cap R_1\ \times\ L_2\cap R_1]}} \hat{a}_{i_1i_2}\right]\ \left[\prod_{(j_3,j_4)\in(\mathcal{L}_2\ \subseteq\ \overline{[L_2\cap R_1\ \times\ L_2\cap R_1]})\backslash\mathcal{R}_1} a_{j_3j_4}\right] \quad (84)$$
$$\times\left[\prod_{(j_7,j_8)\in\mathcal{L}_2\ \cap\ \mathcal{R}_1\ \subseteq\ [L_2\cap R_1\ \times\ L_2\cap R_1]} Z_{j_7j_8}\right]$$

Thus in our case,

$$\left\{\left[\prod_{(j_3,j_4)\in\mathcal{L}_2} a_{j_3j_4}\right]\left[\prod_{(i_1,i_2)\in\mathcal{R}_1} \hat{a}_{i_1i_2}\right]\right\}_{①} = \left[\prod_{(i_1,i_2)\in\mathcal{R}_1\backslash\mathcal{L}_2} \hat{a}_{i_1i_2}\right]\left[\prod_{(j_3,j_4)\in\mathcal{L}_2} a_{j_3j_4}\right]\left[\prod_{(j_7,j_8)\in\mathcal{L}_2\cap\mathcal{R}_1\equiv\mathcal{I}(H_{\text{links}})} Z_{j_7j_8}\right] \quad (85)$$

Here $\mathcal{I}(H_{\text{links}}) \equiv \mathcal{L}_2 \cap \mathcal{R}_1$, or equivalently $H_{\text{links}} \equiv G^{r_1\ \text{out}}_{\text{links}} \cap \mathcal{I}^{-1}(\mathcal{J}(G^{r_2\ \text{in}}_{\text{links}})) = G^{r_1\ \text{out}}_{\text{links}} \cap h^{-1}(G^{r_2\ \text{in}}_{\text{links}})$; likewise $H_{\text{nodes}} \equiv G^{r_1\ \text{out}}_{\text{nodes}} \cap h^{-1}(G^{r_2\ \text{in}}_{\text{nodes}})$.

Thus we have established Lemma 1, which we restate here:

Lemma 1 *$H(S,h)$ must be the* maximal *common subgraph of both $G^{r_1\ out}$ and $G^{r_2\ in}$, for any given choice of nodes S in $G^{r_1\ out}$ and 1-1 corresponding nodes $h(S)$ in $G^{r_2\ in}$. From factor ② we can restrict S to sets of nodes whose labels match in $G^{r_2\ in}_{nodes}$ and $G^{r_1\ out}_{nodes}$. For any such H, we can commute the link operators as follows:*

$$\left[\prod_{(j_3,j_4)\in\mathcal{L}_2} a_{j_3j_4}\right]\left[\prod_{(i_1,i_2)\in\mathcal{R}_1} \hat{a}_{i_1i_2}\right]$$
$$= \left[\prod_{(i_1,i_2)\in\mathcal{I}(G^{r_1\ out}_{links}\backslash H_{links})} \hat{a}_{i_1i_2}\right]\left[\prod_{(j_3,j_4)\in\mathcal{J}(G^{r_2\ in}_{links}\backslash h^{-1}(H_{links}))} a_{j_3j_4}\right]\left[\prod_{(j_7,j_8)\in\mathcal{I}(H_{links})\equiv\mathcal{L}_2\cap\mathcal{R}_1} Z_{j_7j_8}\right] \quad (86)$$

The last factor above augments the graph matching of Equation (83) by implementing the edge-checking or link correspondence portion of graph matching between a subgraph $H(S,h)$ of the output graph of rule r_1 and a corresponding subgraph of the input graph of rule r_2.

Note that the 1-1 and onto node map $h : H \to \tilde{H}$ preserves edges and labels of labeled subgraphs H and $\tilde{H}$, and thus is also an isomorphism of labeled subgraphs.

No proper subsets of $\mathcal{L}_2 \cap \mathcal{R}_1$ from commuting creation and annihilation operators need to be considered, because the Z factor in the last term, arising from Equation (79), is already a sum of two terms: I and $-N$ corresponding to commutation with index miss and hit.

Thus, the "Common(G_1, G_2)" set of shared subgraphs H that we sum over in the graph rewrite commutator is:

Definition Common(G_1, G_2) = An isomorphic pair of (edge-maximal) labeled subgraphs $H_1 \simeq H_2 \simeq H$, with graph embeddings $H_1 \hookrightarrow G_1$ and $H_2 \hookrightarrow G_2$.

From $\mathcal{I}(S) = \mathcal{I}((H_{\text{nodes}}) = L_2 \cap R_1$ and edge-maximality we conclude $\mathcal{I}(H_{\text{links}}) = \mathcal{L}_2 \cap \mathcal{R}_1$, whence Equation (86) becomes

$$\left[\prod_{(j_3,j_4)\in\mathcal{L}_2} a_{j_3j_4}\right]\left[\prod_{(i_1,i_2)\in\mathcal{R}_1} \hat{a}_{i_1i_2}\right] = \left[\prod_{(i_1,i_2)\in\mathcal{L}_2\setminus\mathcal{R}_1} \hat{a}_{i_1i_2}\right]\left[\prod_{(j_3,j_4)\in\mathcal{R}_1\setminus\mathcal{L}_2} a_{j_3j_4}\right]\left[\prod_{(j_7,j_8)\in\mathcal{L}_2\cap\mathcal{R}_1} Z_{j_7j_8}\right] \tag{87}$$

We now assemble partial results of Equations (79), (83), and (87):

$$\begin{aligned}
\hat{W}_{r_2}\hat{W}_{r_1} = & \frac{1}{C_{r_1}(N_{\text{max free}})}\frac{1}{C_{r_2}(N_{\text{max free}})}\rho_{r_1}(\boldsymbol{\lambda}^{(1)},\boldsymbol{\lambda}'^{(1)})\rho_{r_2}(\boldsymbol{\lambda}^{(2)},\boldsymbol{\lambda}'^{(2)}) \\
& \times \sum_{T\subseteq G^{r_1\text{ in}}_{\text{nodes}}\setminus G^{r_1\text{ out}}_{\text{nodes}}} \quad \sum_{\pi:T\overset{1\text{-}1}{\hookrightarrow}G^{r_2\text{ out}}_{\text{nodes}}\setminus G^{r_2\text{ in}}_{\text{nodes}}} \quad \sum_{S\subseteq G^{r_1\text{ out}}_{\text{nodes}}} \quad \sum_{h:S\overset{1\text{-}1}{\hookrightarrow}G^{r_2\text{ in}}_{\text{nodes}}} \\
& \sum_{\mathcal{I}:L_1\cup R_1\overset{1\text{-}1}{\hookrightarrow}\mathcal{U}} \quad \sum_{\substack{\mathcal{J}:L_2\cup R_2\overset{1\text{-}1}{\hookrightarrow}\mathcal{U} \\ \text{Im}(\mathcal{I})\cap\text{Im}(\mathcal{J})=\mathcal{I}(S)\cup\mathcal{I}(T) \\ \mathcal{I}(S)=\mathcal{J}(h(S))=L_2\cap R_1\ \wedge\ \mathcal{I}(T)=\mathcal{J}(\pi(T))}} \left[\prod_{(j_1,j_2)\in\mathcal{R}_2} \hat{a}_{(j_1,j_2)}\right] \\
& \left\{\left[\prod_{(i_1,i_2)\in\mathcal{R}_1\setminus\mathcal{L}_2} \hat{a}_{i_1i_2}\right]\left[\prod_{(j_3,j_4)\in\mathcal{L}_2\setminus\mathcal{R}_1} a_{j_3j_4}\right]\left[\prod_{\substack{(j_7,j_8)\in\mathcal{I}(H_{\text{links}}) \\ \equiv\mathcal{L}_2\cap\mathcal{R}_1}} Z_{j_7j_8}\right]\right\}_{\textcircled{1}}\left[\prod_{(i_3,i_4)\in\mathcal{L}_1} a_{i_3i_4}\right]\left[\prod_{j_5\in R_2} \hat{a}_{j_5,\lambda'^{(2)}_{\mathcal{J}^{-1}(j_5)}}\right] \\
& \times\left\{\left[\prod_{i_{5\,1}\in R_1\setminus L_2} \hat{a}_{i_{5\,1},\lambda'^{(1)}_{\mathcal{I}^{-1}(i_{5\,1})}}\right]\left[\prod_{j_{6\,1}\in L_2\setminus R_1} a_{j_{6\,1},\lambda^{(2)}_{\mathcal{J}^{-1}(j_{6\,1})}}\right]\right. \\
& \times\left[\prod_{i_{5\,0}\in R_1\cap\mathcal{I}(S)=\mathcal{I}(S)} \delta_{\lambda'^{(1)}_{\mathcal{I}^{-1}(i_{5\,0})},\lambda^{(2)}_{h(\mathcal{I}^{-1}(i_{5\,0}))}}\right]\text{(a commuting scalar)} \\
& \left.\times\left[\prod_{i_{5\,0}\in R_1\cap\mathcal{I}(S)=\mathcal{I}(S)} Y_{i_{5\,0},\lambda'^{(1)}_{\mathcal{I}^{-1}(i_{5\,0})}}\right]\right\}_{\textcircled{2}}\left[\prod_{i_6\in L_1} a_{i_6,\lambda^{(1)}_{\mathcal{I}^{-1}(i_6)}}\right]
\end{aligned} \tag{88}$$

Ungrouping $\{\dots\}_{\textcircled{1}}$ and $\{\dots\}_{\textcircled{2}}$, and using the identities $Z_\alpha a_\alpha = a_\alpha$, $Y_\alpha a_\alpha = a_\alpha$, and $(a_\alpha)^2 =$

$0 = (\hat{a}_\alpha)^2$, and regrouping, we get to the point of maximum intermediate expression swell:

$$
\begin{aligned}
\hat{W}_{r_2}\hat{W}_{r_1} = & \frac{1}{C_{r_1}(N_{\text{max free}})}\frac{1}{C_{r_2}(N_{\text{max free}})}\rho_{r_1}(\boldsymbol{\lambda}^{(1)},\boldsymbol{\lambda}'^{(1)})\rho_{r_2}(\boldsymbol{\lambda}^{(2)},\boldsymbol{\lambda}'^{(2)}) \\
& \times \sum_{T\subseteq G^{r_1\ \text{in}}_{\text{nodes}}\setminus G^{r_1\ \text{out}}_{\text{nodes}}} \ \sum_{\pi:T\overset{1\text{-}1}{\hookrightarrow} G^{r_2\ \text{out}}_{\text{nodes}}\setminus G^{r_2\ \text{in}}_{\text{nodes}}} \\
& \times \sum_{S\subseteq G^{r_1\ \text{out}}_{\text{nodes}}} \ \sum_{h:S\overset{1\text{-}1}{\hookrightarrow} G^{r_2\ \text{in}}_{\text{nodes}}} \left[\prod_{i_{5\,0}\in L_2\cap R_1=\mathcal{I}(S)} \delta_{\lambda'^{(1)}_{\mathcal{I}^{-1}(i_{5\,0})},\ \lambda^{(2)}_{h(\mathcal{I}^{-1}(i_{5\,0}))}}\right] \quad // \text{ defines } \sum_H \text{ and adjusts } \frac{1}{C} \\
& \sum_{\mathcal{I}:L_1\cup R_1\overset{1\text{-}1}{\hookrightarrow}\mathcal{U}} \ \sum_{\substack{\mathcal{J}:L_2\cup R_2\overset{1\text{-}1}{\hookrightarrow}\mathcal{U} \\ \mathrm{Im}(\mathcal{I})\cap\mathrm{Im}(\mathcal{J})=\mathcal{I}(S)\cup\mathcal{I}(T) \\ \mathcal{I}(S)=\mathcal{J}(h(S))=L_2\cap R_1\ \wedge\ \mathcal{I}(T)=\mathcal{J}(\pi(T))}} \left\{\left[\prod_{(j_1,j_2)\in\mathcal{R}_2}\hat{a}_{(j_1,j_2)}\right]\left[\prod_{(i_1,i_2)\in\mathcal{R}_1\setminus\mathcal{L}_2}\hat{a}_{i_1 i_2}\right]\right\}_{\textcircled{3}} \\
& \times\left\{\left[\prod_{j_5\in R_2\setminus(R_1\cap\overline{L_2\cap R_1})=R_2\setminus(R_1\setminus L_2)} \hat{a}_{j_5,\lambda'^{(2)}_{\mathcal{J}^{-1}(j_5)}}\right]\left[\prod_{i_{5\,1}\in R_1\setminus L_2=\mathcal{I}(G^{r_1\ \text{out}}_{\text{nodes}}\setminus S)} \hat{a}_{i_{5\,1},\lambda'^{(1)}_{\mathcal{I}^{-1}(i_{5\,1})}}\right]\right\}_{\textcircled{4}} \\
& \times\left\{\left[\prod_{(j_3,j_4)\in\mathcal{L}_2\setminus\mathcal{R}_1} a_{j_3 j_4}\right]\left[\prod_{(i_3,i_4)\in\mathcal{L}_1} a_{i_3 i_4}\right]\right\}_{\textcircled{5}} \\
& \times\left\{\left[\prod_{j_{6\,1}\in L_2\setminus R_1=\mathcal{J}(G^{r_2\ \text{in}}_{\text{nodes}}\setminus h^{-1}(S))} a_{j_{6\,1},\lambda^{(2)}_{\mathcal{J}^{-1}(j_{6\,1})}}\right]\left[\prod_{i_6\in L_1} a_{i_6,\lambda^{(1)}_{\mathcal{I}^{-1}(i_6)}}\right]\right\}_{\textcircled{6}} \\
& \times\left\{\left[\prod_{(j_7,j_8)\in\mathcal{I}(H_{\text{links}})\setminus\mathcal{L}_1\equiv(\mathcal{L}_2\cap\mathcal{R}_1)\setminus\mathcal{L}_1} Z_{j_7 j_8}\right]\left[\prod_{i_{5\,0\,0}\in\mathcal{I}(H_{\text{nodes}})\setminus L_1=(L_2\cap R_1)\setminus L_1} Y_{i_{5\,0\,0},\lambda'^{(1)}_{\mathcal{I}^{-1}(i_{5\,0\,0})}}\right]\right\}_{\textcircled{7}}
\end{aligned}
\tag{89}
$$

Then, using Equation (75) to redefine the index set domains, along with extended indices $i_n^\star$ with $\star$ superscripts to run over them (according to the index map $\mathcal{I}^\star$ which extends $\mathcal{I}$ with nonoverlapping assignments from $\mathcal{J}$ as appropriate); and using the index allocation scheme to

force $T = \varnothing$; we have

$$
\begin{aligned}
\hat{W}_{r_2}\hat{W}_{r_1} =& \frac{1}{C_{r_1}(N_{\text{max free}})}\frac{1}{C_{r_2}(N_{\text{max free}})}\rho_{r_1}(\boldsymbol{\lambda}^{(1)},\boldsymbol{\lambda}'^{(1)})\rho_{r_2}(\boldsymbol{\lambda}^{(2)},\boldsymbol{\lambda}'^{(2)}) \sum_{S\subseteq G^{r_1\,\text{out}}_{\text{nodes}}} \sum_{h:S\overset{1\text{-}1}{\hookrightarrow} G^{r_2\,\text{in}}_{\text{nodes}}} \\
& \sum_{\mathcal{I}:L_1\cup R_1 \overset{1\text{-}1}{\hookrightarrow}\mathcal{U}} \sum_{\substack{\mathcal{J}:L_2\cup R_2\overset{1\text{-}1}{\hookrightarrow}\mathcal{U} \\ \text{Im}(\mathcal{I})\cap\text{Im}(\mathcal{J})=\mathcal{I}(S) \\ \mathcal{I}(S)=\mathcal{J}(h(S))=L_2\cap R_1}} \left[\prod_{i_{5\,0}\in L_2\cap R_1=\mathcal{I}(S)} \delta_{\lambda'^{(1)}_{\mathcal{I}^{-1}(i_{5\,0})},\,\lambda^{(2)}_{h(\mathcal{I}^{-1}(i_{5\,0}))}}\right] \\
& \left[\prod_{(i^\star_1,i^\star_2)\in\mathcal{R}_{1;2}=\mathcal{R}_2\cup(\mathcal{R}_1\backslash\mathcal{L}_2)} \hat{a}_{i_1 i_2}\right]_{③} \left[\prod_{i^\star_1\in R_{1;2}} \hat{a}_{i^\star_1,\,\lambda'^{(1;2)}_{\mathcal{I}^{\star-1}_1(i^\star)}}\right]_{④} \\
& \times\left[\prod_{(i^\star_3,i^\star_4)\in\mathcal{L}_{1;2}=\mathcal{L}_1\cup(\mathcal{L}_2\backslash\mathcal{R}_1)} a_{i_3 i_4}\right]_{⑤} \left[\prod_{i^\star_2\in L_{1;2}} a_{i^\star_2,\,\lambda^{(1;2)}_{\mathcal{I}^{\star-1}(i^\star_2)}}\right]_{⑥} \\
& \times\left\{\left[\prod_{(j_7,j_8)\in\mathcal{D}=(\mathcal{L}_2\cap\mathcal{R}_1)\backslash\mathcal{L}_1\backslash\mathcal{R}_2} Z_{j_7 j_8}\right]\left[\prod_{i_{5\,0\,0}\in\Delta=(L_2\cap R_1)\backslash L_1\backslash R_2} Y_{i_{5\,0\,0},\,\lambda'^{(1)}_{\mathcal{I}^{-1}(i_{5\,0\,0})}}\right]\right\}_{⑦}
\end{aligned}
\tag{90}
$$

Here, by the indexed form for graph grammar rules of Equation (26),

$$
[\dots]_{③}[\dots]_{④} = \hat{a}(G^{r_{1;2}\,\text{out}}) \quad \text{and} \quad [\dots]_{⑤}[\dots]_{⑥} = a(G^{r_{1;2}\,\text{in}}) \tag{91}
$$

as in the non-indexed semantics form of Equation (5). Furthermore, the idempotent factors of Y and Z (diagonal in the number basis, multiplying each pure graph state by 0 or 1) just require sufficient free memory to operate the "churn" of memory used in rule 1 and released in rule 2; assuming index allocation works as designed from a countably infinite store, it is equivalent in the sense of Equation (8) to drop these factors. The Kronecker delta functions are interpreted as label-matching conditions in labeled graph matching as in Lemma 1, constraining the 1-1 correspondence map h to respect the node labels and thus (again by Lemma 1) to be an isomorphism of labeled graphs; and they also help to ensure that the normalization for number of equivalent outcome graphs is correct. Thus in order to compute the labeled, numbered graph rewrite rule in each summand over S and h, one needs to find a labeled subgraph H of $G^{r_1\,\text{out}}$ that is isomorphic as a labeled graph to a labeled subgraph $\tilde{H}$ of $G^{r_2\,\text{in}}$, and do this in an edge-maximal way; then one needs to pick an isomorphism h between H and $\tilde{H}$; then using H, $\tilde{H}$ and h to map between the rule r_1 and r_2 node numberings, one needs to compute the left hand side and right hand labeled graphs as numbered and labeled node sets and link sets.

Thus by careful interpretation of terms we arrive at the main result, except limited to the case in which hanging edges are *not* removed by the rule semantics: for the hanging-edge permissive semantics of Equations (5) and (6), or equivalently Equation (26),

$$
\hat{W}_{G^{r_2\,\text{in}}\to G^{r_2\,\text{out}}}\hat{W}_{G^{r_1\,\text{in}}\to G^{r_1\,\text{out}}} \simeq \sum_{\substack{H\subseteq G^{r_1\,\text{out}}\simeq\tilde{H}\subseteq G^{r_2\,\text{in}} \\ \mid\ \text{edge-maximal}}} \sum_{h:H\overset{1\text{-}1}{\hookrightarrow}\tilde{H}} \hat{W}_{G^{r_1\,\text{in}}\dot\cup(G^{r_2\,\text{in}}\backslash\tilde{H})\underset{h}{\to}G^{r_2\,\text{out}}\dot\cup(G^{r_1\,\text{out}}\backslash H)} \tag{92}
$$

In more detail, the summand graph rewrite rule is then defined by the disjoint unions $\dot\cup$ (re-

flecting time-reversal $L \leftrightarrow R$ duality):

$$\begin{aligned}
G^{1;2\,\text{in}}_{\text{nodes}}(\tilde{H}_{\text{nodes}}) &= G^{r_1\,\text{in}}_{\text{nodes}} \dot{\cup} (G^{r_2\,\text{in}}_{\text{nodes}} \backslash \tilde{H}_{\text{nodes}}) & G^{1;2\,\text{out}}_{\text{nodes}}(H_{\text{nodes}}) &= G^{r_2\,\text{out}}_{\text{nodes}} \dot{\cup} (G^{r_1\,\text{out}}_{\text{nodes}} \backslash H_{\text{nodes}}) \\
&\equiv G^{r_1\,\text{in}}_{\text{nodes}} \cup h^{-1\star}(G^{r_2\,\text{in}}_{\text{nodes}} \backslash \tilde{H}_{\text{nodes}}) & &\equiv G^{r_2\,\text{out}}_{\text{nodes}} \cup h^{\star}(G^{r_1\,\text{out}}_{\text{nodes}} \backslash H_{\text{nodes}}) \\
G^{1;2\,\text{in}}_{\text{links}}(\tilde{H}_{\text{nodes}}) &= G^{r_1\,\text{in}}_{\text{links}} \cup h^{-1\star}(G^{r_2\,\text{in}}_{\text{links}} \backslash \tilde{H}_{\text{links}}) & G^{1;2\,\text{out}}_{\text{links}}(H_{\text{nodes}}) &= G^{r_2\,\text{out}}_{\text{links}} \cup h^{\star}(G^{r_1\,\text{out}}_{\text{links}} \backslash H_{\text{links}})
\end{aligned} \tag{93}$$

where $\dot{\cup}$ denotes disjoint union, and where $h^\star$: extends the labeled graph isomorphism $h : H \subseteq G^{r_1\,\text{out}} \to \tilde{H} \subseteq G^{r_2\,\text{in}}$ to a map on nodes and links (not necessarily a graph homomorphism) $h^\star : G^{r_1\,\text{out}} \to G^{1;2\,\text{out}}$ by remapping the nodes of G^{r_1} along h if possible, and to the disjoint union nodes if not, and preserving all possible links except those in H_{links} since they are subject to editing by rule r_2; and likewise for $h^{-1} : \tilde{H} \subseteq G^{r_2\,\text{in}} \to H \subseteq G^{r_1\,\text{out}}$ and $h^{-1\star} : G^{r_2\,\text{in}} \to G^{1;2\,\text{in}}$. The conserved core graphs are determined by shared node labels on the left and right of a rule:

$$\begin{aligned}
K_a &= G^{r_a\,\text{in}}_{\text{nodes}} \cap G^{r_a\,\text{out}}_{\text{nodes}} \\
K_{1;2} &= (K_1 \backslash H_{\text{nodes}}) \;\cup\; h^{-1}(K_2 \backslash \tilde{H}_{\text{nodes}}) \;\cup\; (K_1 \cap h^{-1\star}(K_2))
\end{aligned} \tag{94}$$

The exact mechanics of graph numbering and disjoint union are discussed in [3], and examples are given in Section 3.7.

Recall that Equations (12), (13), and (14) take care of updating the integrals over parameters that we have omitted from the composition of semantics calculation starting in Section 3.4, by using up all the commutator-derived delta functions on extra label parameters. Then, given the definitions of the compound label graphs in Equations (93) and (94), one can write the graph rewrite rule algebra as announced in Section 2.4 and Theorem 1:

Theorem 1 *For the hanging-edge-permissive semantics of Equations (5) and (6), or equivalently Equation (26), and assuming multiplicative normalization C_r, then*

$$\hat{W}_{G^{r_2\,in} \to G^{r_2\,out}} \hat{W}_{G^{r_1\,in} \to G^{r_1\,out}} \simeq \sum_{\substack{H \subseteq G^{r_1\,out} \simeq \tilde{H} \subseteq G^{r_2\,in} \\ \mid \text{ edge-maximal}}} \; \sum_{h : H \overset{1\text{-}1}{\hookrightarrow} \tilde{H}} \hat{W}_{G^{1;2\,in}(\tilde{H}) \underset{h}{\to} G^{1;2\,out}(H)} \tag{95}$$

where the compound labeled graphs $G^{1;2\,in}(\tilde{H})$ and $G^{1;2\,out}(H)$, and their label overlaps $K_{1;2}$, are defined by Equations (93) and (94) above. The coefficients in this expression are all nonnegative integers (as the same graph grammar rule could arise several times by different means). Rate factors ρ multiply with parameter substitution, as in Equation (14).

From this theorem we derived a series of corollaries in Section 3, which comprise key conclusions of this paper.

SA.6 Commutation calculation - with edge cleanup

We now turn to the hanging-edge cleanup semantics, and prove (Theorem 2) that the same algebra as in Theorem 1 and Equations (93), (94), and (95), above still applies.

The semantics is now (restating Equation (96))

$$\begin{aligned}\hat{W}_{r_\chi} &= \frac{1}{C_{r_\chi}(N_{\text{max free}})} \int d\mu_{r_\chi}(X)\, \rho_{r_\chi}(\boldsymbol{\lambda}[X], \boldsymbol{\lambda}'[X]) \sum_{\mathcal{I}_\chi : L_\chi \cup R_\chi \overset{1\text{-}1}{\hookrightarrow} \mathcal{U}} \left[\Big(\prod_{(i',i)\in\mathcal{P}_\chi} E_{i'\,i} \Big) \Big(\prod_{(\hat{\imath},\hat{\imath}')\in\mathcal{P}^*_\chi} E_{\hat{\imath}\,\hat{\imath}'} \Big) \right] \\ &\quad \times \left[\prod_{(i_1,i_2)\in\mathcal{R}_\chi} \hat{a}_{i_1 i_2} \right] \left[\prod_{(i_3,i_4)\in\mathcal{L}_\chi} a_{i_3 i_4} \right] \left[\prod_{i_5 \in R_\chi} \hat{a}_{i_5\,,\,\lambda'_{\mathcal{I}_\chi^{-1}(i_5)}} \right] \left[\prod_{i_6 \in L_\chi} a_{i_6\,,\,\lambda_{\mathcal{I}_\chi^{-1}(i_6)}} \right].\end{aligned} \tag{96}$$

The product of two such operators is (omitting for now the integral over parameters X)

$$\begin{aligned}\hat{W}_{r_2}\hat{W}_{r_1} &= \frac{1}{C_{r_1}(N_{\text{max free}})} \frac{1}{C_{r_2}(N_{\text{max free}})} \rho_{r_1}(\boldsymbol{\lambda}^{(1)}, \boldsymbol{\lambda}'^{(1)}) \rho_{r_2}(\boldsymbol{\lambda}^{(2)}, \boldsymbol{\lambda}'^{(2)}) \\ &\quad \times \sum_{\mathcal{J} : L_2 \cup R_2 \overset{1\text{-}1}{\hookrightarrow} \mathcal{U}} \; \sum_{\mathcal{I} : L_1 \cup R_1 \overset{1\text{-}1}{\hookrightarrow} \mathcal{U}} \left[\Big(\prod_{(j',j)\in\mathcal{P}_2} E_{j'\,j} \Big) \Big(\prod_{(\hat{\jmath},\hat{\jmath}')\in\mathcal{P}^*_2} E_{\hat{\jmath}\,\hat{\jmath}'} \Big) \right] \\ &\quad \times \left[\prod_{(j_1,j_2)\in\mathcal{R}_2} \hat{a}_{j_1 j_2} \right] \left[\prod_{(j_3,j_4)\in\mathcal{L}_2} a_{j_3 j_4} \right] \left[\prod_{j_5 \in R_2} \hat{a}_{j_5\,,\,\lambda'^{(2)}_{\mathcal{J}^{-1}(j_5)}} \right] \left[\prod_{j_6 \in L_2} a_{j_6\,,\,\lambda^{(2)}_{\mathcal{J}^{-1}(j_6)}} \right] \\ &\quad \times \left[\Big(\prod_{(i',i)\in\mathcal{P}_1} E_{i'\,i} \Big) \Big(\prod_{(\hat{\imath},\hat{\imath}')\in\mathcal{P}^*_1} E_{\hat{\imath}\,\hat{\imath}'} \Big) \right] \\ &\quad \times \left[\prod_{(i_1,i_2)\in\mathcal{R}_1} \hat{a}_{i_1 i_2} \right] \left[\prod_{(i_3,i_4)\in\mathcal{L}_1} a_{i_3 i_4} \right] \left[\prod_{i_5 \in R_1} \hat{a}_{i_5\,,\,\lambda'^{(1)}_{\mathcal{I}^{-1}(i_5)}} \right] \left[\prod_{i_6 \in L_1} a_{i_6\,,\,\lambda^{(1)}_{\mathcal{I}^{-1}(i_6)}} \right]\end{aligned} \tag{97}$$

The problem is to treat the potentially very high degree factors of $\prod_{\mathcal{P}_1 \cup \mathcal{P}_1^*} E$ that have been inserted into the middle of this semantics.

SA.6.1 Edge cleanup asymptotics

We now work to replace the product of E_{ij} factors above with the exponential of a sum.

$$E_\alpha = Z_\alpha + a_\alpha = I_\alpha + (a_\alpha - N_\alpha) = I_\alpha + W_{\alpha\to\varnothing} \tag{98}$$

First we note an application of the Euler formula for the matrix exponential. Defining

$$\tau = \rho_{\text{erase}} t\ , \tag{99}$$

where ρ_{erase} is an effective high speed of interpolated edge erasures, then

$$\begin{aligned}\exp\left(\tau \sum_{\alpha\in\mathcal{S}} W_{\alpha\to\varnothing} \right) &= \lim_{m\to\infty} \Big(I + \frac{\tau}{m} \sum_{\alpha\in\mathcal{S}} W_{\alpha\to\varnothing} \Big)^m = \lim_{m\to\infty} \Big(\prod_{\alpha\in\mathcal{S}} (I + \frac{\tau}{m} W_{\alpha\to\varnothing}) \Big)^m \\ &= \prod_{\alpha\in\mathcal{S}} \Big(\lim_{m\to\infty} (I + \frac{\tau}{m} W_{\alpha\to\varnothing})^m \Big)\end{aligned} \tag{100}$$

where the product orders are arbitrary because different $W_{\alpha\to\varnothing}$ commute. Defining $\epsilon = \tau/m$, another expression for this is

$$\exp\left(\tau\sum_{\alpha\in\mathcal{S}} W_{\alpha\to\varnothing}\right) = \lim_{m\to+\infty,\epsilon->0^+}\left(\prod_{\alpha\in\mathcal{S}}(I+\epsilon W_{\alpha\to\varnothing})\right)^m. \tag{101}$$

On the other hand, recalling that E_α, N_α, Z_α and I_α are all idempotent ($E^2 = E$, etc.) in the 2×2 case,

$$\begin{aligned}\exp\left(\tau\sum_{\alpha\in\mathcal{S}} W_{\alpha\to\varnothing}\right) &= \exp\left(\tau\sum_{\alpha\in\mathcal{S}}(E_\alpha - I_\alpha)\right) = \exp\left(-\tau\sum_{\alpha\in\mathcal{S}} I_\alpha\right)\exp\left(\tau\sum_{\alpha\in\mathcal{S}} E_\alpha\right)\\ &= \exp(-\tau|\mathcal{S}|)[I + \tau\sum_{\alpha\in\mathcal{S}} E_\alpha + \frac{\tau^2}{2}[\sum_{\alpha\in\mathcal{S}} E_\alpha + \sum_{\alpha\neq\beta\in\mathcal{S}} E_\alpha E_\beta] + \cdots]\\ &= \exp(-\tau|\mathcal{S}|)\left[\sum_{k=0}^{\infty}\frac{\tau^k}{k!}\sum_{\alpha_1\ldots\alpha_k\in\mathcal{S}} E_{\alpha_1}\ldots E_{\alpha_k}\right]\end{aligned} \tag{102}$$

Using $E_\alpha^2 = E_\alpha$ and grouping α_*s into partition blocks of equal α value,

$$\begin{aligned}\exp\left(\tau\sum_{\alpha\in\mathcal{S}} W_{\alpha\to\varnothing}\right) &= \exp(-\tau|\mathcal{S}|)\left[\sum_{k=0}^{\infty}\frac{\tau^k}{k!}\sum_{\alpha_1\ldots\alpha_k\in\mathcal{S}} E_{\alpha_1}\ldots E_{\alpha_k}\right]\\ &= \exp(-\tau|\mathcal{S}|)\left[I + \sum_{k=1}^{\infty}\frac{\tau^k}{k!}\sum_{l=1}^{\min(k,|\mathcal{S}|)}\left\{{k \atop l}\right\}\sum_{\langle\beta_1\ldots\beta_l\in\mathcal{S}\rangle_{\neq}} E_{\beta_1}\ldots E_{\beta_k}\right]\\ &= \exp(-\tau|\mathcal{S}|)\left[I + \sum_{l=1}^{|\mathcal{S}|}\sum_{k=l}^{\infty}\frac{\tau^k}{k!}\left\{{k \atop l}\right\}\sum_{\langle\beta_1\ldots\beta_l\in\mathcal{S}\rangle_{\neq}} E_{\beta_1}\ldots E_{\beta_k}\right]\\ &= \exp(-\tau|\mathcal{S}|)\left[I + \sum_{l=1}^{|\mathcal{S}|}\frac{(e^\tau-1)^l}{l!}\sum_{\langle\beta_1\ldots\beta_l\in\mathcal{S}\rangle_{\neq}} E_{\beta_1}\ldots E_{\beta_k}\right]\end{aligned} \tag{103}$$

where $\left\{{k \atop l}\right\}$ are Stirling numbers of the second kind and where the last line uses a generating function for these numbers.

Then asymptotically as $\tau = \rho_{\text{erase}} t \to +\infty$, and defining $|\mathcal{S}|_{(m)} \equiv |\mathcal{S}|!/(|\mathcal{S}|-m)!$, where $m + l = |\mathcal{S}|$,

$$\begin{aligned}\exp\left(\tau\sum_{\alpha\in\mathcal{S}} W_{\alpha\to\varnothing}\right) &\to \exp(-\tau|\mathcal{S}|)I + \frac{1}{|\mathcal{S}|!}\sum_{m=0}^{|\mathcal{S}|-1}|\mathcal{S}|_{(m)}e^{-mt}\sum_{\langle\beta_1\ldots\beta_{|\mathcal{S}|-m}\in\mathcal{S}\rangle_{\neq}} E_{\beta_1}\ldots E_{\beta_{|\mathcal{S}|-m}}\\ &\to \frac{1}{|\mathcal{S}|!}\sum_{\langle\beta_1\ldots\beta_{|\mathcal{S}|}\in\mathcal{S}\rangle_{\neq}} E_{\beta_1}\ldots E_{\beta_{|\mathcal{S}|}} = \prod_{\alpha\in\mathcal{S}} E_\alpha.\end{aligned} \tag{104}$$

So, *complete erasure is the limiting behavior of this edge-by-edge stochastic erasure process*, and it can be achieved simply by taking the limit $\rho_{\text{erase}} \to +\infty$.

Now we apply these calculations to the actual hanging-edge erasure operator:

$$\exp\left(\tau \sum_{(i_1,i_2)\in\mathcal{S}} W_{(i_1,i_2)\to\varnothing}\right) = \exp\left(\tau \sum_{(i_1,i_2)\in\mathcal{S}} (E_{i_1,i_2} - I_{i_1,i_2}) N_{i_2} Z_{i_1}\right) \tag{105}$$

Here the node operator Z_i checks for unallocated nodes i with no label:

$$\begin{aligned}
Z_i &\equiv \prod_\lambda Y_{i,\lambda} = N_{i,\varnothing} \prod_\lambda Z_{i,\lambda} = N_{i,\varnothing} \prod_\lambda (I - N_{i,\lambda}) && (106)\\
&\simeq N_{i,\varnothing}(I - \sum_\lambda N_{i,\lambda}) \quad \text{(since } N_{i,*} \text{ cross-terms vanish from WTA)} && (107)\\
&\simeq N_{i,\varnothing} \cdot N_{i,\varnothing} \quad \text{(from Equation (63), top line)} && (108)\\
Z_i &= N_{i,\varnothing} && (109)
\end{aligned}$$

whence $Z_i Z_i = Z_i$. Also $N_i \equiv \sum_\lambda N_{i,\lambda}$ counts the number of active labels for node i which by WTA constraint is 0 or 1; we have again $N_i N_i = N_i$ and $N_i + Z_i = I$ and $N_i Z_i = 0$. We note here that the operator Z as defined above *doesn't* quite fit within the graph grammar rule semantics we have defined so far because it checks for *non*existence. Nonexistence checks are identified as a more general kind of semantics in [1] and [3], which we do not treat in the present work except for this particular technical example. Of course, Equation (105) doesn't need to fit within the rule semantics, as it is not explicitly accessible at the level of stochastic parameterized graph grammar rules - it is just substructure.

Again defining $\epsilon = \tau/m$, another expression for the exponential in Equation (105) is

$$\exp\left(\tau \sum_{\alpha\in\mathcal{S}} W_{(i_1,i_2)\in\mathcal{S}\to\varnothing}\right) = \lim_{m\to+\infty,\epsilon->0^+} \Big(\prod_{(i_1,i_2)\in\mathcal{S}} (I + \epsilon W_{(i_1,i_2)\in\mathcal{S}\to\varnothing})\Big)^m \tag{110}$$

On the other hand,

$$\begin{aligned}
\exp(\tau \sum_{(i,j)\in\mathcal{S}} W_{(i_1,i_2)\to\varnothing}) &= \exp\left(\tau \sum_{(i,j)\in\mathcal{S}} (E_{i,j} - I_{i,j}) N_j Z_i\right) = \exp\left(-\tau \sum_{(i,j)\in\mathcal{S}} N_j Z_i\right) \exp\left(\tau \sum_{(i_1,i_2)\in\mathcal{S}} E_{i,j} N_j Z_i\right)\\
&= \exp(-\tau|\mathcal{S}|)\left[\sum_{k=0}^{\infty} \frac{\tau^k}{k!} \sum_{(i_1,j_1)\dots(i_k,j_k)\in\mathcal{S}} (E_{i_1,j_1}\dots E_{i_k,j_k})(N_{j_1}\dots N_{j_k})(Z_{i_1}\dots Z_{i_k})\right]\\
&= \exp(-\tau|\mathcal{S}|)\left[I + \sum_{k=1}^{\infty} \frac{\tau^k}{k!} \sum_{l=1}^{\min(k,|\mathcal{S}|)} \left\{ {k \atop l} \right\} \sum_{\langle (i_1,j_1)\dots(i_l,j_l)\in\mathcal{S}\rangle_{\neq}} (E_{i_1,j_1}\dots E_{i_l,j_l})(N_{j_1}\dots N_{j_l})(Z_{i_1}\dots Z_{i_l})\right]\\
&= \exp(-\tau|\mathcal{S}|)\left[I + \sum_{l=1}^{|\mathcal{S}|} \sum_{k=l}^{\infty} \frac{\tau^k}{k!} \left\{ {k \atop l} \right\} \sum_{\langle (i_1,j_1)\dots(i_l,j_l)\in\mathcal{S}\rangle_{\neq}} (E_{i_1,j_1}\dots E_{i_l,j_l})(N_{j_1}\dots N_{j_l})(Z_{i_1}\dots Z_{i_l})\right]\\
&= \exp(-\tau|\mathcal{S}|)\left[I + \sum_{l=1}^{|\mathcal{S}|} \frac{(e^\tau - 1)^l}{l!} \sum_{\langle (i_1,j_1)\dots(i_l,j_l)\in\mathcal{S}\rangle_{\neq}} (E_{i_1,j_1}\dots E_{i_l,j_l})(N_{j_1}\dots N_{j_l})(Z_{i_1}\dots Z_{i_l})\right]
\end{aligned}$$

(111)

where as before $\left\{ {k \atop l} \right\}$ are Stirling numbers of the second kind and where the last line uses a generating function for these numbers. Then asymptotically as $\tau = \rho_{\text{erase}} t \rightarrow +\infty$, and defining $|\mathcal{S}|_{(m)} \equiv |\mathcal{S}|!/(|\mathcal{S}| - m)!$,

$$
\begin{aligned}
\exp\left(\tau \sum_{\alpha \in \mathcal{S}} W_{\alpha \rightarrow \varnothing}\right) &\rightarrow \exp(-\tau|\mathcal{S}|) I + \frac{1}{|\mathcal{S}|!} \sum_{m=0}^{|\mathcal{S}|-1} |\mathcal{S}|_{(m)} e^{-m\tau} \\
&\qquad \sum_{\langle (i_1,j_1) \dots (i_l,j_l) \in \mathcal{S} \rangle_{\neq}} (E_{i_1,j_1} \dots E_{i_l,j_l})(N_{j_1} \dots N_{j_l})(Z_{i_1} \dots Z_{i_l}) \\
&\rightarrow \frac{1}{|\mathcal{S}|!} \sum_{\langle (i_1,j_1) \dots (i_l,j_l) \in \mathcal{S} \rangle_{\neq}} (E_{i_1,j_1} \dots E_{i_l,j_l})(N_{j_1} \dots N_{j_l})(Z_{i_1} \dots Z_{i_l}) \\
&= \prod_{(i,j) \in \mathcal{S}} E_{i,j} N_j Z_i \simeq \prod_{(i,j) \in \mathcal{P}} E_{i,j} \; .
\end{aligned}
\tag{112}
$$

The final expression above is a key step prepared for by the discussion in Section SA.4.3, and it is justified by the fact that inductively the operator N_j produces a zero value unless node j has been allocated at some point in the history of rule-firings .

So again we get the product of forward edge erasures by an incremental process of deletion, run for a long effective time τ.

SA.6.2 Commutation with edge cleanup

In Equation (97), as in (72),

$$
\begin{aligned}
\hat{W}^{\text{cleaned}} &= \Big(\prod_{(k_1,k_2) \in \mathcal{S}} E_{k_1 k_2} E_{k_2 k_1} \Big) \hat{W}^{\text{bare}} \\
&= \lim_{n \rightarrow +\infty, \epsilon \rightarrow 0^+} [I + \epsilon \sum_{(k_1,k_2) \in \mathcal{S}} (a_{k_1,k_2} - N_{k_1,k_2}) N_{k_2} Z_{k_1}]^n [I + \epsilon \sum_{(k_1,k_2) \in \mathcal{S}} (a_{k_2,k_1} - N_{k_2,k_1}) N_{k_1} Z_{k_2}]^n \hat{W}^{\text{bare}}
\end{aligned}
\tag{113}
$$

The core calculation within $\hat{W}^{\text{cleaned}}_{r_2} \cdot \hat{W}^{\text{cleaned}}_{r_1}$ is thus:

$$
\begin{aligned}
\hat{W}^{\text{bare}}_{r_2} [\epsilon \sum_{(k_1,k_2) \in \mathcal{S}} (a_{k_1,k_2} - N_{k_1,k_2}) N_{k_2} Z_{k_1}] &= \frac{\epsilon}{C_{r_2}} \sum_{\mathcal{I}} \sum_{(k_1,k_2) \in \mathcal{S}} \Big[\prod_{(i_1,i_2) \in \mathcal{R}_2} \hat{a}_{i_1 i_2} \Big] \Big[\prod_{(i_3,i_4) \in \mathcal{L}_2} a_{i_3 i_4} \Big] (a_{k_1,k_2} - N_{k_1,k_2}) \\
&\quad \times \Big[\prod_{i_5 \in R_2} \hat{a}_{i_5, \lambda_{\mathcal{I}^{-1}(i_5)}} \Big] \Big[\prod_{i_6 \in L_2} a_{i_6, \lambda_{\mathcal{I}^{-1}(i_6)}} \Big] N_{k_2} Z_{k_1}
\end{aligned}
\tag{114}
$$

Now calculate components:

Graph Nodes:

$$
\Big[\prod_{i_6 \in L_2} a_{i_6, \lambda_{\mathcal{I}^{-1}(i_6)}} \Big] N_{k_2} = \begin{cases} [\prod_{i_6 \in L_2} a_{i_6, \lambda_{\mathcal{I}^{-1}(i_6)}}] & \text{if } k_2 \in L_2 \\ N_{k_2} [\prod_{i_6 \in L_2} a_{i_6, \lambda_{\mathcal{I}^{-1}(i_6)}}] & \text{if } k_2 \notin L_2 \end{cases}
\tag{115}
$$

$$\Big[\prod_{i_5\in R_2}\hat{a}_{i_5,\lambda_{\mathcal{I}^{-1}(i_5)}}\Big]N_{k_2}=\begin{cases}0 & \text{if } k_2\in R_2\\ N_{k_2}[\prod_{i_5\in R_2}\hat{a}_{i_5,\lambda_{\mathcal{I}^{-1}(i_5)}}] & \text{if } k_2\notin R_2\end{cases}\tag{116}$$

so

$$\Big[\prod_{i_5\in R_2}\hat{a}_{i_5,\lambda_{\mathcal{I}^{-1}(i_5)}}\Big]\Big[\prod_{i_6\in L_2}a_{i_6,\lambda_{\mathcal{I}^{-1}(i_6)}}\Big]N_{k_2}=\begin{cases}0 & \text{if } k_2\in R_2\backslash L_2\\ \Big[\prod_{i_5\in R_2}\hat{a}_{i_5,\lambda_{\mathcal{I}^{-1}(i_5)}}\Big]\Big[\prod_{i_6\in L_2}a_{i_6,\lambda_{\mathcal{I}^{-1}(i_6)}}\Big] & \text{if } k_2\in L_2\\ N_{k_2}\Big[\prod_{i_5\in R_2}\hat{a}_{i_5,\lambda_{\mathcal{I}^{-1}(i_5)}}\Big]\Big[\prod_{i_6\in L_2}a_{i_6,\lambda_{\mathcal{I}^{-1}(i_6)}}\Big] & \text{if } k_2\in\overline{L_2}\cap\overline{R_2}\end{cases}\tag{117}$$

Likewise $Z_k=I-N_k \implies N_k=I-Z_k$ and

$$\Big[\prod_{i_6\in L_2}a_{i_6,\lambda_{\mathcal{I}^{-1}(i_6)}}\Big]Z_{k_1}=\begin{cases}0 & \text{if } k_1\in L_2\\ Z_{k_1}[\prod_{i_6\in L_2}a_{i_6,\lambda_{\mathcal{I}^{-1}(i_6)}}] & \text{if } k_2\notin L_2\end{cases}\tag{118}$$

$$\Big[\prod_{i_5\in R_2}\hat{a}_{i_5,\lambda_{\mathcal{I}^{-1}(i_5)}}\Big]Z_{k_1}=\begin{cases}[\prod_{i_5\in R_2}\hat{a}_{i_5,\lambda_{\mathcal{I}^{-1}(i_5)}}] & \text{if } k_2\in R_2\\ Z_{k_1}[\prod_{i_5\in R_2}\hat{a}_{i_5,\lambda_{\mathcal{I}^{-1}(i_5)}}] & \text{if } k_2\notin R_2\end{cases}\tag{119}$$

so

$$\Big[\prod_{i_5\in R_2}\hat{a}_{i_5,\lambda_{\mathcal{I}^{-1}(i_5)}}\Big]\Big[\prod_{i_6\in L_2}a_{i_6,\lambda_{\mathcal{I}^{-1}(i_6)}}\Big]Z_{k_1}=\begin{cases}0 & \text{if } k_1\in L_2\\ \Big[\prod_{i_5\in R_2}\hat{a}_{i_5,\lambda_{\mathcal{I}^{-1}(i_5)}}\Big]\Big[\prod_{i_6\in L_2}a_{i_6,\lambda_{\mathcal{I}^{-1}(i_6)}}\Big] & \text{if } k_1\in R_2\backslash L_2\\ Z_{k_1}\Big[\prod_{i_5\in R_2}\hat{a}_{i_5,\lambda_{\mathcal{I}^{-1}(i_5)}}\Big]\Big[\prod_{i_6\in L_2}a_{i_6,\lambda_{\mathcal{I}^{-1}(i_6)}}\Big] & \text{if } k_1\in\overline{L_2}\cap\overline{R_2}\end{cases}\tag{120}$$

Together, then,

$$\boxed{\begin{aligned}&\Big[\prod_{i_5\in R_2}\hat{a}_{i_5,\lambda_{\mathcal{I}^{-1}(i_5)}}\Big]\Big[\prod_{i_6\in L_2}a_{i_6,\lambda_{\mathcal{I}^{-1}(i_6)}}\Big]N_{k_2}Z_{k_1}\\ &\quad=\begin{cases}0 & \text{if } (k_1\in L_2)\vee(k_2\in R_2\backslash L_2)\\ \Big[\prod_{i_5\in R_2}\hat{a}_{i_5,\lambda_{\mathcal{I}^{-1}(i_5)}}\Big]\Big[\prod_{i_6\in L_2}a_{i_6,\lambda_{\mathcal{I}^{-1}(i_6)}}\Big] & \text{if } (k_1\in R_2\backslash L_2)\wedge(k_2\in L_2)\\ Z_{k_1}\Big[\prod_{i_5\in R_2}\hat{a}_{i_5,\lambda_{\mathcal{I}^{-1}(i_5)}}\Big]\Big[\prod_{i_6\in L_2}a_{i_6,\lambda_{\mathcal{I}^{-1}(i_6)}}\Big] & \text{if } (k_1\in\overline{L_2}\cap\overline{R_2})\wedge(k_2\in L_2)\\ N_{k_2}\Big[\prod_{i_5\in R_2}\hat{a}_{i_5,\lambda_{\mathcal{I}^{-1}(i_5)}}\Big]\Big[\prod_{i_6\in L_2}a_{i_6,\lambda_{\mathcal{I}^{-1}(i_6)}}\Big] & \text{if } (k_1\in R_2\backslash L_2)\wedge(k_2\in\overline{L_2}\cap\overline{R_2})\\ N_{k_2}Z_{k_1}\Big[\prod_{i_5\in R_2}\hat{a}_{i_5,\lambda_{\mathcal{I}^{-1}(i_5)}}\Big]\Big[\prod_{i_6\in L_2}a_{i_6,\lambda_{\mathcal{I}^{-1}(i_6)}}\Big] & \text{if } (k_1\in\overline{L_2}\cap\overline{R_2})\wedge(k_2\in\overline{L_2}\cap\overline{R_2}).\end{cases}\end{aligned}}\tag{121}$$

Graph Links: We continue to calculate

$$\Big[\prod_{(i_3,i_4)\in\mathcal{L}_2}a_{i_3i_4}\Big]a_{k_1,k_2}=\begin{cases}0 & \text{if } (k_1,k_2)\in\mathcal{L}_2\\ a_{k_1,k_2}[\prod_{(i_3,i_4)\in\mathcal{L}_2}a_{i_3i_4}] & \text{if } (k_1,k_2)\notin\mathcal{L}_2\end{cases}\tag{122}$$

and

$$\Big[\prod_{(i_1,i_2)\in\mathcal{R}_2}\hat{a}_{i_1i_2}\Big]a_{k_1,k_2} = \begin{cases} N_{k_1,k_2}[\prod_{(i_1,i_2)\in\mathcal{R}_2\setminus(i_1,i_2)}\hat{a}_{i_1i_2}] & \text{if } (k_1,k_2)\in\mathcal{R}_2 \\ a_{k_1,k_2}[\prod_{(i_1,i_2)\in\mathcal{R}_2}\hat{a}_{i_1i_2}] & \text{if } (k_1,k_2)\notin\mathcal{R}_2 \end{cases} \tag{123}$$

Next,

$$\Big[\prod_{(i_3,i_4)\in\mathcal{L}_2}a_{i_3i_4}\Big]\hat{a}_{k_1,k_2} = \begin{cases} Z_{k_1,k_2}[\prod_{(i_3,i_4)\in\mathcal{L}_2\setminus(k_1,k_2)}a_{i_3i_4}] & \text{if } (k_1,k_2)\in\mathcal{L}_2 \\ \hat{a}_{k_1,k_2}[\prod_{(i_3,i_4)\in\mathcal{L}_2}a_{i_3i_4}] & \text{if } (k_1,k_2)\notin\mathcal{L}_2 \end{cases} \tag{124}$$

and since $Z_\alpha a_\alpha = a_\alpha$,

$$\Big[\prod_{(i_3,i_4)\in\mathcal{L}_2}a_{i_3i_4}\Big]N_{k_1,k_2} = \Big[\prod_{(i_3,i_4)\in\mathcal{L}_2}a_{i_3i_4}\Big]\hat{a}_{k_1,k_2}a_{k_1,k_2} = \begin{cases} [\prod_{(i_3,i_4)\in\mathcal{L}_2}a_{i_3i_4}] & \text{if } (k_1,k_2)\in\mathcal{L}_2 \\ N_{k_1,k_2}[\prod_{(i_3,i_4)\in\mathcal{L}_2}a_{i_3i_4}] & \text{if } (k_1,k_2)\notin\mathcal{L}_2 \end{cases} \tag{125}$$

so from Equations (122) and (125),

$$\Big[\prod_{(i_3,i_4)\in\mathcal{L}_2}a_{i_3i_4}\Big](a_{k_1,k_2}-N_{k_1,k_2}) = \begin{cases} -[\prod_{(i_3,i_4)\in\mathcal{L}_2}a_{i_3i_4}] & \text{if } (k_1,k_2)\in\mathcal{L}_2 \\ (a_{k_1,k_2}-N_{k_1,k_2})[\prod_{(i_3,i_4)\in\mathcal{L}_2}a_{i_3i_4}] & \text{if } (k_1,k_2)\notin\mathcal{L}_2 \end{cases} \tag{126}$$

Likewise:

$$\Big[\prod_{(i_1,i_2)\in\mathcal{R}_2}\hat{a}_{i_1i_2}\Big]N_{k_1,k_2} = \begin{cases} 0 & \text{if } (k_1,k_2)\in\mathcal{R}_2 \\ N_{k_1,k_2}[\prod_{(i_1,i_2)\in\mathcal{R}_2}\hat{a}_{i_1i_2}] & \text{if } (k_1,k_2)\notin\mathcal{R}_2 \end{cases} \tag{127}$$

so from Equations (123) and (126),

$$\Big[\prod_{(i_1,i_2)\in\mathcal{R}_2}\hat{a}_{i_1i_2}\Big](a_{k_1,k_2}-N_{k_1,k_2}) = \begin{cases} N_{k_1,k_2}[\prod_{(i_1,i_2)\in\mathcal{R}_2}\hat{a}_{i_1i_2}] & \text{if } (k_1,k_2)\in\mathcal{R}_2 \\ (a_{k_1,k_2}-N_{k_1,k_2})[\prod_{(i_1,i_2)\in\mathcal{R}_2}\hat{a}_{i_1i_2}] & \text{if } (k_1,k_2)\notin\mathcal{R}_2 \end{cases} \tag{128}$$

Combining,

$$\boxed{\begin{aligned} &\Big[\prod_{(i_1,i_2)\in\mathcal{R}_2}\hat{a}_{i_1i_2}\Big]\Big[\prod_{(i_3,i_4)\in\mathcal{L}_2}a_{i_3i_4}\Big](a_{k_1,k_2}-N_{k_1,k_2}) \\ &\qquad = \begin{cases} -\Big[\prod_{(i_1,i_2)\in\mathcal{R}_2}\hat{a}_{i_1i_2}\Big]\Big[\prod_{(i_3,i_4)\in\mathcal{L}_2}a_{i_3i_4}\Big] & \text{if } (k_1,k_2)\in\mathcal{L}_2 \\ N_{k_1,k_2}\Big[\prod_{(i_1,i_2)\in\mathcal{R}_2}\hat{a}_{i_1i_2}\Big]\Big[\prod_{(i_3,i_4)\in\mathcal{L}_2}a_{i_3i_4}\Big] & \text{if } (k_1,k_2)\in\mathcal{R}_2\setminus\mathcal{L}_2 \\ (a_{k_1,k_2}-N_{k_1,k_2})\Big[\prod_{(i_1,i_2)\in\mathcal{R}_2}\hat{a}_{i_1i_2}\Big]\Big[\prod_{(i_3,i_4)\in\mathcal{L}_2}a_{i_3i_4}\Big] & \text{if } (k_1,k_2)\in\overline{\mathcal{R}}_2\cap\overline{\mathcal{L}}_2. \end{cases}\end{aligned}} \tag{129}$$

Next we argue: If $(k_1,k_2)\in\overline{\mathcal{R}}_2\cap\overline{\mathcal{L}}_2$ as in the third line of the right hand side of Equation (129) above then the commutation was successful, and the factor of $a-N$ simply joins the infinite supply of such factors to the left. That leaves two cases in Equation (129). If $(k_1,k_2)\in\mathcal{L}_2$ (as in the first line of the right hand side of Equation (129) above) then $k_1\in L_2\wedge k_2\in L_2$ so in Equation (121) the first line applies and the term is zero; it doesn't contribute. That leaves one case: $(k_1,k_2)\in\mathcal{R}_2\setminus\mathcal{L}_2$, in which case $k_1\in R_2\wedge k_2\in R_2$. Then either the first line the right hand side

of in Equation (121) again applies and eliminates the present term, or else neither of its alternative conditions apply and $k_1 \in R_2 \backslash L_2 \wedge k_2 \in R_2 \cap L_2$; thus the condition for a surviving prefactor of N_{k_1,k_2} is:

$$(k_1, k_2) \in [R_2 \backslash L_2 \times L_2 \cap R_2] \cap (\mathcal{R}_2 \backslash \mathcal{L}_2) \tag{130}$$

... a condition excluded by the index allocation scheme, which implies $k_1 \notin R_2 \backslash L_2$. So, all surviving terms behave as in the third line of Equation (129), and the factor of $a - N$ to the right of the second rule firing simply joins the infinite supply of such factors to its left.

Intuitively, this means that hanging edges can be eliminated nonspecifically by an overactive syntax-checking process, rather than surgically in a way that depends on the details of each rule firing, because the assumed form of the graph rewrite rules does not recognize or respond to hanging edges.

Thus we find no change to the algebraic formula of Theorem 1 (i.e. Theorem 1) for the hanging-edge removal semantics:

Theorem 2 *For the hanging-edges removal semantics of Equations (23) and (24), or equivalently Equation (96), and assuming finiteness of rules, index allocation blocks, and number of rule firings, and assuming multiplicative normalization C_r, then*

$$\hat{W}_{G^{r_2\,in} \to G^{r_2\,out}} \hat{W}_{G^{r_1\,in} \to G^{r_1\,out}} \simeq \sum_{\substack{H \subseteq G^{r_1\,out} \simeq \tilde{H} \subseteq G^{r_2\,in} \\ \mid \text{ edge-maximal}}} \sum_{h: H \overset{1-1}{\hookrightarrow} \tilde{H}} \hat{W}_{G^{1;2\,in}(\tilde{H}) \underset{h}{\to} G^{1;2\,out}(H)} \tag{131}$$

where the compound labeled graphs $G^{1;2\,in}(\tilde{H})$ and $G^{1;2\,out}(H)$, and their label overlaps $K_{1;2}$, are defined by Equations (93) and (94) above. The coefficients in this expression are all nonnegative integers (as the same graph grammar rule could arise several times by different means). Rate factors ρ multiply with parameter substitution, as in Equation (90).

From this theorem we derived a series of corollaries in Section 3 above, which comprise key conclusions of this paper.

Appendix B Supplementary Material : Stochastic graph grammar examples

This section expands on the cortical microtubule graph grammar of the main text, and calculates more commutators.

SB.1 MT Stochastic graph grammar examples

A diagrammatic presentation of a microtubule (MT) network graph grammar, with subscripts for the rule-local arbitrary but consistent numbering of vertices in left- and right-hand side graphs of each rule, is below. Discrete parameters will include a four-valued categorical label $s \in$ {internal, grow_end,

(or $s \in \{\circ, \bullet, \blacksquare, \blacktriangle\}$) for status as interior segment, growth-capable end segment, retraction-capable end segment, or bundling junction segment respectively.

$$
\begin{aligned}
&\text{// Rule 1: Treadmilling growth}\\
&(\ \bullet_1)\langle\!\langle(x_1, u_1)\rangle\!\rangle \longrightarrow (\ \circ_1 \to \bullet_2)\langle\!\langle(x_1, u_1), (x_2, u_2)\rangle\!\rangle\\
&\quad\textbf{with}\ \hat{\rho}_{\text{grow}}([\text{Y}_g])\mathcal{N}(x_1 - x_2; Lu_1, \sigma)\mathcal{N}_{|u_2|=1}(u_2; u_1), \epsilon),\\
&\text{// Rule 2: Treadmilling retraction}\\
&(\ \blacksquare_1 \to \circ_2)\langle\!\langle(x_1, u_1), (x_2, u_2)\rangle\!\rangle \longrightarrow (\ \blacksquare_2)\langle\!\langle(x_2, u_2)\rangle\!\rangle\\
&\quad\textbf{with}\ \hat{\rho}_{\text{retract}}([\text{Y}_r])\\
&\text{// Rule 3: Collision-induced bundling or zippering}\\
&\begin{pmatrix} \circ_1 \longrightarrow \circ_2 \to \circ_3 \\ \\ \bullet_4 \end{pmatrix}\langle\!\langle(x_1, u_1), (x_2, u_2), (x_3, u_3), (x_4, u_4)\rangle\!\rangle\\
&\quad\longrightarrow \begin{pmatrix} \circ_1 \longrightarrow \blacktriangle_2 \to \circ_3 \\ \nearrow \\ \circ_4 \end{pmatrix}\langle\!\langle(x_1, u_1), (x_2, u_2), (x_3, u_3), (x_4, u_4)\rangle\!\rangle\\
&\quad\textbf{with}\ \hat{\rho}_{\text{bundle}}(|u_2 \cdot u_4|/|\cos\theta_{\text{crit}}|)\exp\left(-|x_2 - x_4|^2/2L^2\right)\\
&\text{// Rule 4: Katanin-induced severing}\\
&(\ \circ_1 \to \circ_2 \to \circ_3)\langle\!\langle(x_1, u_1), (x_2, u_2), (x_3, u_3)\rangle\!\rangle\\
&\quad\longrightarrow (\ \circ_1 \to \bullet_2 \quad \blacksquare_4 \to \circ_3)\langle\!\langle x_1, u_1), (x_2, u_2), (x_3, u_3), (x_4, u_4)\rangle\!\rangle\\
&\quad\textbf{with}\ \hat{\rho}_{\text{sever}}([\text{katanin}])\mathcal{N}(x; \mathbf{0}, \sigma_{\text{broad}})\delta_{\text{Dirac}}(|u| - 1))
\end{aligned}
\tag{132}
$$

Here Y_g is a diffusible MT growth factor such as tubulin itself, or a catalyst or regulator of tubulin polymerization and/or nucleation, such as (perhaps) XMAP215 [7] and Y_r plays the same role for catastrophe/retraction.

In working out the commutators we will drop the propensity functions ρ, but they just multiply the results, with appropriate variable identifications.

SB.2 Selected MT commutator calculations

The commutator calculations for the minimal MT graph grammar's Lie algebra can be outlined as follows.

$[\hat{W}_2, \hat{W}_1]$:

$\hat{W}_2 \cdot \hat{W}_1$: Shared same-label vertex sets run over by H and their mappings under h are: $\varnothing$; $\{(1 \mapsto 2)\}$.

$\hat{W}_1 \cdot \hat{W}_1$: Shared same-label vertex sets run over by H and their mappings under h are: $\varnothing$.

$H = \varnothing$ always cancels in the commutator.

More detailed work lets us calculate $[\hat{W}_2, \hat{W}_1] =$ by abusing notation slightly:

$$
\begin{aligned}
[\hat{W}_2, \hat{W}_1] &= [(\ \blacksquare_{1'} \to \circ_{2'}) \longrightarrow (\ \blacksquare_{2'})\ ,\ (\ \bullet_1) \longrightarrow (\ \circ_1 \to \bullet_2)]\\
&\simeq \quad (\ \blacksquare_{1'} \to \bullet_1) \longrightarrow (\ \blacksquare_1 \to \bullet_2)
\end{aligned}
\tag{133}
$$

which is just a renumbering of the same graph, which provided that the model-specific rules of MT representation are respected by the other grammar rules, should be equivalent to the identity

operator. The corresponding full $W = \hat{W} - D$ operator should therefore (by Corollary 1 to Theorem 1) be equivalent to the zero operator, using this model-specific extension of the equivalence relation in Equation (8).

$[\hat{W}_3, \hat{W}_1]$: Calculated in the main text.

$[\hat{W}_4, \hat{W}_1]$:
$\hat{W}_4 \cdot \hat{W}_1$: Shared same-label vertex sets run over by H and their mappings under h are: $\varnothing$; $\{(1 \mapsto 1')\}$; $\{(1 \mapsto 2')\}$; $\{(1 \mapsto 3')\}$;
$\hat{W}_1 \cdot \hat{W}_4$: Shared same-label vertex sets run over by H and their mappings under h are: $\varnothing$; $\{(2' \mapsto 1)\}$.
$H = \varnothing$ always cancels in the commutator.

$$\begin{aligned}
[\hat{W}_4, \hat{W}_1] = [(\bigcirc_{1'} \to \bigcirc_{2'} \to \bigcirc_{3'}) &\longrightarrow (\bigcirc_{1'} \to \bullet_{2'} \ \blacksquare_{4'} \to \bigcirc_{3'}) \ , \ (\bullet_1) \longrightarrow (\bigcirc_1 \to \bullet_2)] \\
\simeq \quad (\bigcirc_{1'} \to \bigcirc_{2'} \to \bullet_1) &\longrightarrow (\bigcirc_{1'} \to \bullet_{2'} \ \blacksquare_{4'} \to \bigcirc_1 \to \bullet_2) \\
- (\bigcirc_{1'} \to \bigcirc_{2'} \to \bigcirc_{3'}) &\longrightarrow (\bigcirc_{1'} \to \bigcirc_{2'} \to \bullet_2 \ \blacksquare_{4'} \to \bigcirc_{3'}) \\
&+ (\text{2 terms whose LHS rely on MT syntax violations - omitted})
\end{aligned} \tag{134}$$

The foregoing commutators can also be calculated directly by operator algebra, bypassing the general theorems, with the same results.

$[\hat{W}_4, \hat{W}_3]$:

$$\begin{aligned}
[\hat{W}_4, \hat{W}_3] = [(\bigcirc_{1'} \to \bigcirc_{2'} \to \bigcirc_{3'}) &\longrightarrow (\bigcirc_{1'} \to \bullet_{2'} \ \blacksquare_{4'} \to \bigcirc_{3'}) \ , \\
\begin{pmatrix} \bigcirc_1 \longrightarrow \bigcirc_2 \to \bigcirc_3 \\ \\ \bullet_4 \end{pmatrix} &\longrightarrow \begin{pmatrix} \bigcirc_1 \longrightarrow \blacktriangle_2 \to \bigcirc_3 \\ \nearrow \\ \bigcirc_4 \end{pmatrix}] \simeq \ldots
\end{aligned} \tag{135}$$

$\hat{W}_3 \cdot \hat{W}_4$: Shared same-label vertex sets run over by H and their mappings under h are: $\varnothing$; $\{(1' \mapsto 1)\}$; $\{(1' \mapsto 2)\}$; $\{(1' \mapsto 3)\}$; $\{(3' \mapsto 1)\}$; $\{(3' \mapsto 2)\}$; $\{(3' \mapsto 3)\}$; $\{(1' \mapsto i), (3' \mapsto j)\}$ where unordered sets $\{i, j\}$ are chosen without replacement from $\{1, 2, 3\}$ (6 possibilities); $\{(4' \mapsto 4)\}$ simultaneously with any of the foregoing 12 possibilities; Thus, $4 \times 6 = 24$ terms.
$\hat{W}_4 \cdot \hat{W}_3$: Shared same-label vertex sets run over by H and their mappings under h are: $\varnothing$; $\{(i \mapsto j)\}$ where i is chosen from $\{1, 3, 4\}$ and j is chosen from $\{1', 2', 3'\}$ ($3 \times 3 = 9$ possibilities); also $\{(i \mapsto k), (j \mapsto l)\}$ where unordered pairs $\{i, j\}$ are chosen without replacement from $\{1, 3, 4\}$ (3 possibilities) and ordered pairs (k, l) are chosen without replacement from $\{1', 2', 3'\}$ (6 possibilities, for a total of $3 \times 6 = 18$ possibilities); $\{(1 \mapsto i), (3 \mapsto j), (4 \mapsto k)\}$ where ordered sets $\{i, j, k\}$ are chosen without replacement from $\{1', 2', 3'\}$ (6 possibilities). Thus, there are $9 + 18 + 6 = 33$ terms.
$H = \varnothing$ always cancels in the commutator. Other cancellations are possible, since the scalar propensity functions multiply commutatively, leaving at most $24 + 33 = 57$ terms. As before, many of these terms will have no effect within a grammar that preserves inductively valid MT representation structures.

SB.3 ODE Commutators

Here we illustrate Proposition 1 by exhibiting products and commutators involving differential equation rule operators, first among themselves and then with conventional SPG (non-DE) graph

grammar rule operators.

SB.3.1 [ODE, ODE] commutators

In like manner to Section SB.2 above and as a check, we can compute the commutator of two differential equation rules that affect the same one-node graph with the same type parameter and a continuous vector variable $\boldsymbol{x}$. Other ODE rules can be reduced to this case by parameter replication across objects, and this case is easier to calculate. Similarly we will assume for simplicity $d\mu_r(x) = d\boldsymbol{x}$ and $d\mu_r(y) = d\boldsymbol{y}$ in Euclidean space.

If the two differential equations are $d\boldsymbol{x}/dt = \boldsymbol{v}_1(\boldsymbol{x})$ and $d\boldsymbol{x}/dt = \boldsymbol{v}_2(\boldsymbol{x})$, then there is an $H = \varnothing$ commutator miss term that cancels out and a $H =$ the one node term that contributes to the commutator. We assume that $\boldsymbol{v}_1, \boldsymbol{v}_2$ and their derivatives fall off at infinity sufficiently fast that we may drop all boundary terms upon integration by parts. Then an exercise in Dirac delta function integration shows that the commutator of these two graph grammar variants of differential operators, $[\mathcal{O}_{\mathrm{DE}\,2}, \mathcal{O}_{\mathrm{DE}\,1}]$, corresponds to another such operator $\mathcal{O}_{\mathrm{DE}\,[2,1]}$ with differential equation right hand side:

$$\boldsymbol{v}_{[2,1]}(\boldsymbol{x}) = (\boldsymbol{v}_1 \cdot \boldsymbol{\nabla}_{\boldsymbol{x}})\boldsymbol{v}_2(\boldsymbol{x}) - (\boldsymbol{v}_2 \cdot \boldsymbol{\nabla}_{\boldsymbol{x}})\boldsymbol{v}_1(\boldsymbol{x}) \; . \tag{136}$$

This result may be understood as follows. Under the Baker-Cambell-Hausdorff formula

$$e^{t\mathcal{O}_{\mathrm{DE}\,2}} e^{t\mathcal{O}_{\mathrm{DE}\,1}} \simeq e^{t(\mathcal{O}_{\mathrm{DE}\,2}+\mathcal{O}_{\mathrm{DE}\,2})+(t^2/2)[\mathcal{O}_{\mathrm{DE}\,2},\mathcal{O}_{\mathrm{DE}\,1}]+O(t^3)} \; , \tag{137}$$

we can adjust $e^{t(\mathcal{O}_{\mathrm{DE}\,2}+\mathcal{O}_{\mathrm{DE}\,2})}$ to equal $e^{t\mathcal{O}_{\mathrm{DE}\,2}} e^{t\mathcal{O}_{\mathrm{DE}\,1}}$ if we move the $\boldsymbol{v}_2$ contribution forwards in time evolution under $\boldsymbol{v}_1$, and also move the $\boldsymbol{v}_1$ contribution backwards in time evolution under $\boldsymbol{v}_2$. As a check, the same result arises by letting $e^{t\mathcal{O}_{\mathrm{DE}\,\chi}}$ act on probability densities $\rho(\boldsymbol{x})$ as $\mathcal{O}_{\mathrm{DE}\,\chi} = -\mathcal{D}_\chi = -\boldsymbol{v}_\chi(\boldsymbol{x}) \cdot \boldsymbol{\nabla}_{\boldsymbol{x}}$, and computing the commutator. The operator product $\mathcal{O}_{\mathrm{DE}\,2} \cdot \mathcal{O}_{\mathrm{DE}\,1}$ can be expressed as an $\mathcal{O}_{\mathrm{DE}}$ plus additional symmetric diffusion term [2] which cancels out in the commutator.

SB.3.2 [ODE, SPG] commutators

The operators we want to multiply are again

$$\hat{W}_{\mathrm{SPG}\,1} = \frac{1}{C_1(N_{\mathrm{max\ free}})} \int d\mu_1(X)\, \rho_1(X) \sum_{\langle i_1,\ldots i_k\rangle_{\neq}} \hat{a}_{i_1,\ldots i_k}(G^{(1)\,\mathrm{out}}) a_{i_1,\ldots i_k}(G^{(1)\,\mathrm{in}}) \tag{138}$$

and

$$W_{\mathrm{ODE}\,2} = \hat{W}_{\mathrm{ODE}\,2} = \int d\boldsymbol{x} d\boldsymbol{y}\, \rho_2(\boldsymbol{y},\boldsymbol{x}) \sum_{\langle i_1,\ldots i_k\rangle_{\neq}} \hat{a}_{i_1,\ldots i_k}(G^{(2)}(\boldsymbol{y})) a_{i_1,\ldots i_k}(G^{(2)}(\boldsymbol{x})) \;, \text{where} \tag{139a}$$

$$\rho_2(\boldsymbol{y},\boldsymbol{x}) = -\boldsymbol{\nabla}_{\boldsymbol{y}} \cdot (\boldsymbol{v}(\boldsymbol{y})\delta(\boldsymbol{y}-\boldsymbol{x})) = -\sum_a \boldsymbol{\nabla}_{y_a}(v_a(\boldsymbol{y}) \prod_b \delta(y_b - x_b)) \quad . \tag{139b}$$

For simplicity only, we will make the same assumptions as in Section SB.3.1 above, that the ODE rule operates on a one-node graph $G^{(2)}$, with parameter $\boldsymbol{x}$ going in to the rule and parameter $\boldsymbol{y}$ coming out of it, so that the subgraphs H are either $H = G^{(2)}$ or $H = \varnothing$. Since $H = \varnothing$ cancels out of the commutator we will just calculate the "main" $H = G^{(2)}$ contribution to the products. Also

$d\mu_r(x) = d\boldsymbol{x}$ and $d\mu_r(y) = d\boldsymbol{y}$ in Euclidean space. Since the ODE rule doesn't change $N_{\text{max free}}$, the normalization will be unchanged as well.

In $\hat{W}_{\text{SPG 1}}$ we can factor the independent parameter vector X into a parameter vector $\boldsymbol{z}_{h\|}$ of continuous-valued parameters which labels the node matched by h to the singleton node of $G^{(2)}$, and a parameter vector $X_{h\perp}$ which captures all other floating parameters of $G^{(1)}$. In the notation of Equation (14) of the main text, $\boldsymbol{z}_{h\|}$ will play the role of Z, the vector of matched label parameter values, which get matched by appropriate Dirac delta functions and substituted into both ρ functions, except that Z may also have additional discrete "user-defined type" information involved in matching but not in ODE dynamics. $\boldsymbol{z}_{h\|}$ will match either $\boldsymbol{x}$ or $\boldsymbol{y}$, depending on whether $\hat{W}_{\text{SPG 1}}$ comes before (acts to the right of) $W_{\text{ODE 2}}$ or after (to the left).

Now for any given match h we can split the integral over the independent parts of X, possibly at the expense of re-expressing $\rho_1(X)$:

$$\hat{W}_{\text{SPG (1)h}} = \frac{1}{C_1(N_{\text{max free}})} \int d\mu_{(1)h}(X_{h\perp}) \int d\boldsymbol{z}_{h\|}\ \rho_1(\boldsymbol{z}_{h\|}, X_{h\perp}) \sum_{\langle i_1,\dots i_k\rangle_{\neq}} \hat{a}_{i_1,\dots i_k}(G^{(1)\ \text{out}}) a_{i_1,\dots i_k}(G^{(1)\ \text{in}}) \tag{140}$$

Then using the notation of Theorem 1 to combine $G^{(1)}$ and $G^{(2)}$ into $G^{2;1\ \text{in}}$ and $G^{2;1\ \text{out}}$, and adding label substitution information to those labeled graphs as $G^{2;1\ \text{in}}(\tilde{H}, \boldsymbol{x}, \boldsymbol{z}_{h\perp})$ and $G^{2;1\ \text{out}}(H, \boldsymbol{y}, \boldsymbol{z}_{h\perp})$ respectively,

$$\begin{aligned}\hat{W}_{\text{SPG (1)}} W_{\text{ODE (2)}} = \frac{1}{C_1(N_{\text{max free}})} \sum_{h:H=G^{(2)}\to\tilde{H}\in G^{(1)}} \int d\mu_{(1)h}(X_{h\perp}) \int d\boldsymbol{z}_{h\|} \int d\boldsymbol{x} \int d\boldsymbol{y} \\ \rho_1(\boldsymbol{z}_{h\|} = \boldsymbol{y}, X_{h\perp}) \rho_2(\boldsymbol{y}, \boldsymbol{x}) \hat{W}_{G^{2;1\ \text{in}}(\tilde{H},\boldsymbol{x},\boldsymbol{z}_{h\perp}) \underset{h}{\to} G^{2;1\ \text{out}}(H,\boldsymbol{y},\boldsymbol{z}_{h\perp})} \quad ,\end{aligned} \tag{141}$$

where again

$$\rho_2(\boldsymbol{y}, \boldsymbol{x}) = -\boldsymbol{\nabla}_{\boldsymbol{y}} \cdot (\boldsymbol{v}(\boldsymbol{y}) \delta(\boldsymbol{y} - \boldsymbol{x})) = -\sum_a \boldsymbol{\nabla}_{y_a} (v_a(\boldsymbol{y}) \prod_b \delta(y_b - x_b)) \quad . \tag{142}$$

Similarly,

$$\begin{aligned}W_{\text{ODE (2)}} \hat{W}_{\text{SPG (1)}} = \frac{1}{C_1(N_{\text{max free}})} \sum_{h:H\in G^{(1)}\to\tilde{H}=G^{(2)}} \int d\mu_{(1)h}(X_{h\perp}) \int d\boldsymbol{z}_{h\|} \int d\boldsymbol{x} \int d\boldsymbol{y} \\ \rho_1(\boldsymbol{z}_{h\|} = \boldsymbol{x}, X_{h\perp}) \rho_2(\boldsymbol{y}, \boldsymbol{x}) \hat{W}_{G^{1;2\ \text{in}}(\tilde{H},\boldsymbol{x},\boldsymbol{z}_{h\perp}) \underset{h}{\to} G^{1;2\ \text{out}}(H,\boldsymbol{y},\boldsymbol{z}_{h\perp})} \quad .\end{aligned} \tag{143}$$

Finally, the commutator is the difference which factors if we abuse notation a bit and let h stand also for h^{-1}, since it is a 1-1 onto map:

$$\begin{aligned}[W_{\text{ODE (2)}}, \hat{W}_{\text{SPG (1)}}] = \frac{1}{C_1(N_{\text{max free}})} \sum_{h:H\to\tilde{H}} \int d\mu_{(1)h}(X_{h\perp}) \int d\boldsymbol{z}_{h\|} \int d\boldsymbol{x} \int d\boldsymbol{y} \quad \rho_2(\boldsymbol{y}, \boldsymbol{x}) \\ \times \Big\{ \rho_1(\boldsymbol{z}_{h\|} = \boldsymbol{x}, X_{h\perp}) \hat{W}_{G^{1;2\ \text{in}}(\tilde{H},\boldsymbol{x},\boldsymbol{z}_{h\perp}) \underset{h}{\to} G^{1;2\ \text{out}}(H,\boldsymbol{y},\boldsymbol{z}_{h\perp})} \\ - \rho_1(\boldsymbol{z}_{h\|} = \boldsymbol{y}, X_{h\perp}) \hat{W}_{G^{2;1\ \text{in}}(\tilde{H},\boldsymbol{x},\boldsymbol{z}_{h\perp}) \underset{h}{\to} G^{2;1\ \text{out}}(H,\boldsymbol{y},\boldsymbol{z}_{h\perp})} \Big\} \quad ,\end{aligned} \tag{144}$$

with the same ODE-specifying ρ_1 as before, just supplied with different initial conditions $\boldsymbol{x}$ or $\boldsymbol{y}$ as indicated.

Appendix C Supplementary Material : Operators and commutators of pure chemical reaction operators

If none of the graphs involved has any edges, then each rule transforms a collection of nodes, partitioned into indistinguishable subsets by their labels, into another such set – and this is equivalent to a pure stochastic chemical reaction network. The algebra of elementary creation/annihilation operators is the Heisenberg algebra $[a, \hat{a}] = I$ for each chemical species i. What is the algebra of the reaction rules? Each reaction rule or channel has off-diagonal operator [8, 9, 10]:

$$\hat{W}_r = \hat{W}_{\{m_i^{(r)}\} \to \{n_i^{(r)}\}} = k^{(r)} \prod_i (\hat{a}_i)^{n_i^{(r)}} (a_i)^{m_i^{(r)}} \tag{145}$$

so a product of such operators is

$$\hat{W}_{r_2} \hat{W}_{r_1} = k^{(r_2)} k^{(r_1)} \prod_i (\hat{a}_i)^{n_i^{(r_2)}} (a_i)^{m_i^{(r_2)}} (\hat{a}_i)^{n_i^{(r_1)}} (a_i)^{m_i^{(r_1)}} \tag{146}$$

For in-principle unbounded numbers n of identical molecules of one molecular species, $n_{\max} = +\infty$, the law of mass action is encoded in the Heisenberg algebra

$$[a, \hat{a}] = a\hat{a} - \hat{a}a = I \tag{147}$$

since for $n > 0$ both $\hat{a}a$ and $a\hat{a}$ leave any state vector direction $|n\rangle$ unchanged (so the operator is diagonal in the number basis) but the former has a relative propensity of $n+1$ vs. n for the latter [8, 9]; these diagonal entries subtract to give the identity operator I. (And if $n = 0$ then the first term is I and the second term is 0.) The cases in which $n_{\max}$ is an arbitrary finite integer are presented in [1]. In the graph grammar semantics we use $n_{\max} = 1$, which can be taken to be more "fundamental". But here, $n_{\max} = +\infty$.

The middle two terms $(a_i)^{m_i^{(r_2)}} (\hat{a}_i)^{n_i^{(r_1)}}$ of Equation (146) can be put into canonical form by mapping the Heisenberg algebra into generating functions, $a \to \partial_x, \hat{a} \to x \times \ldots$:

$$\begin{aligned} a^m \hat{a}^n \to & [(\partial_x)^m x^n] \circ f(x) = (\partial_x)^m (x^n f(x)) \\ = & \sum_{l=0}^{\min(m,n)} \binom{m}{l} ({\partial_x}^l x^n)({\partial_x}^{m-l} f(x)) = \sum_{l=0}^{\min(m,n)} \binom{m}{l} (n)_l x^{n-l} ({\partial_x}^{m-l} f(x)) \\ \leftarrow & \sum_{l=0}^{\min(m,n)} \frac{(m)_l (n)_l}{l!} \hat{a}^{n-l} a^{m-l} \end{aligned} \tag{148}$$

where $(n)_l \equiv n!/(n-l)!$ for $l \leqslant n$. If we define also $n_l \equiv 0$ for $l > n$ then we can increase or remove the upper limit, e.g. replace min by max

Then

$$\hat{W}_{r_2}\hat{W}_{r_1} = k^{(r_2)}k^{(r_1)}\prod_i\Big[\sum_{l_i=0}^{\min(m_i^{(r_2)},n_i^{(r_1)})}\frac{(m_i^{(r_2)})_l(n_i^{(r_1)})_l}{l_i!}(\hat{a}_i)^{n_i^{(r_1)}+n_i^{(r_2)}-l_i}(a_i)^{m_i^{(r_1)}+m_i^{(r_2)}-l_i}\Big]$$
$$= k^{(r_2)}k^{(r_1)}\sum_{\{l_i=0\ldots\min(m_i^{(r_2)},n_i^{(r_1)})\}}\Big(\prod_i\frac{(m_i^{(r_2)})_l(n_i^{(r_1)})_l}{l_i!}\Big)\Big[\prod_i(\hat{a}_i)^{n_i^{(r_1)}+n_i^{(r_2)}-l_i}(a_i)^{m_i^{(r_1)}+m_i^{(r_2)}-l_i}\Big] \tag{149}$$

i.e.

$$\boxed{\begin{aligned}\hat{W}_{\{m_i^{(r_2)}\}\to\{n_i^{(r_2)}\}}\hat{W}_{\{m_i^{(r_1)}\}\to\{n_i^{(r_1)}\}} = k^{(r_2)}k^{(r_1)}&\sum_{\{l_i=0\ldots\min(m_i^{(r_2)},n_i^{(r_1)})\}}\Big(\prod_i\frac{(m_i^{(r_2)})_l(n_i^{(r_1)})_l}{l_i!}\Big)\\ &\times\hat{W}_{\{(m_i^{(r_1)}+m_i^{(r_2)}-l_i)\}\to\{(n_i^{(r_1)}+n_i^{(r_2)}-l_i)\}}\end{aligned}} \tag{150}$$

Likewise

$$\boxed{\begin{aligned}&[\hat{W}_{\{m_i^{(r_2)}\}\to\{n_i^{(r_2)}\}},\hat{W}_{\{m_i^{(r_1)}\}\to\{n_i^{(r_1)}\}}]\\ &\quad= k^{(r_2)}k^{(r_1)}\sum_{\{l_i=0\ldots\min(m_i^{(r_2)},n_i^{(r_1)})\}\wedge\mathbf{l}\neq\mathbf{0}}\Bigg[\Big(\prod_i\frac{(m_i^{(r_2)})_l(n_i^{(r_1)})_l}{l_i!}\Big)-\Big(\prod_i\frac{(m_i^{(r_1)})_l(n_i^{(r_2)})_l}{l_i!}\Big)\Bigg]\\ &\quad\times\hat{W}_{\{(m_i^{(r_1)}+m_i^{(r_2)}-l_i)\}\to\{(n_i^{(r_1)}+n_i^{(r_2)}-l_i)\}}\end{aligned}} \tag{151}$$

where $\mathbf{l}\neq\mathbf{0}$ is the particle analog of Corollaries 4 or 8 regarding the cancellation of $H=\varnothing$ from a graph grammar commutator.

Related calculations have been done [11] for reaction-diffusion systems on a grid, with different notation and a specialization to the relevant case of finding the commutator between the sum of all reaction operators and the sum of all discrete diffusion operators treated as uni-uni (one molecule in, one out) reactions.

References

References, using the numbering of the main paper where possible:

[1] E. Mjolsness and G. Yosiphon, "Stochastic Process Semantics for Dynamical Grammars", Annals of Mathematics and Artificial Intelligence, 47(3-4) August 2006.

[2] E. Mjolsness, "Towards Measurable Types for Dynamical Process Modeling Languages". Proceedings of the 26th Conference on Mathematical Foundations of Programming Semantics (MFPS 2010). Electronic Notes in Theoretical Computer Science (ENTCS), Elsevier, vol. 265, pp. 123-144, 6 Sept. 2010.

[3] E. Mjolsness, “Prospects for Declarative Mathematical Modeling of Complex Biological Systems”. Bulletin of Mathematical Biology, Vol. 81, Issue 8, pp 3385-3420, August 2019.

[4] R. P. Feynman, “Quantum mechanical computers” Foundations of Physics, Volume 16, Issue 6, pp 507-531. June 1986. See Hamiltonian on p. 517.

[5] E. Mjolsness, D. H. Sharp, and J. Reinitz, “A Connectionist Model of Development”. Journal of Theoretical Biology, vol 152 no 4, pp. 429-454, 1991.

[6] E. Mjolsness, “Symbolic Neural Networks Derived from Stochastic Grammar Domain Models”, in Connectionist Symbolic Integration, eds. R. Sun and F. Alexandre, Lawrence Erlbaum Associates, 1997.

[7] O. Hamant, D. Inoue, D. Bouchez, J. Dumais, and E. Mjolsness, “Are microtubules tension sensors?” Nature Communications, v10 article no. 2360, 29 May 2019.

[8] M. Doi, Journal of Physics A: Mathematical and General 9, 1465 (1976).

[9] M. Doi, Journal of Physics A: Mathematical and General 9, 1479 (1976).

[10] D. C. Mattis and M. L. Glasser, Rev. Mod. Phys. 70, 979 (1998).

[11] Hellander, Lawson, Drawert, Petzold. J. Comp. Physics, v 266, 1 June 2014.
